# A systematic review and meta-analyses of the temporal stability and convergent validity of risk preference measures

**DOI:** 10.1038/s41562-024-02085-2

**Published:** 2025-01-27

**Authors:** Alexandra Bagaïni, Yunrui Liu, Madlaina Kapoor, Gayoung Son, Paul-Christian Bürkner, Loreen Tisdall, Rui Mata

**Affiliations:** 1 Faculty of Psychology, University of Basel, Basel, Switzerland; 2 Department of Psychology, University of Bern, Bern, Switzerland; 3 Department of Statistics, TU Dortmund University, Dortmund, Germany

**Keywords:** Human behaviour, Economics

## Abstract

Understanding whether risk preference represents a stable, coherent trait is central to efforts aimed at explaining, predicting and preventing risk-related behaviours. We help characterize the nature of the construct by adopting a systematic review and individual participant data meta-analytic approach to summarize the temporal stability of 358 risk preference measures (33 panels, 57 samples, 579,114 respondents). Our findings reveal noteworthy heterogeneity across and within measure categories (propensity, frequency and behaviour), domains (for example, investment, occupational and alcohol consumption) and sample characteristics (for example, age). Specifically, while self-reported propensity and frequency measures of risk preference show a higher degree of stability than behavioural measures, these patterns are moderated by domain and age. Crucially, an analysis of convergent validity reveals a low agreement across measures, questioning the idea that they capture the same underlying phenomena. Our results raise concerns about the coherence and measurement of the risk preference construct.

## Abstract (web summary)

Bagaïni et al. use an individual participant data meta-analytic approach to summarize the temporal stability of 358 risk preference measures, finding evidence of low agreement across measures.

## Main

Risk permeates all domains and stages of life. Risk preference—an umbrella term reflecting an individual’s appetite for risk^1,2^—is related to consequential personal decisions (for example, the timing of marriage and parenthood)^3^ and financial decisions^4^, and may be used as an indicator to match individuals with products, services and suitable careers^5–8^. Because of its broad relevance for shaping individuals’ health, wealth and happiness, risk preference is central to many theories and applications in the behavioural sciences^9,10^.

Despite the construct’s importance, its central characteristics continue to be discussed, including whether risk preference represents a stable, coherent trait or rather a contextual and/or domain-specific disposition^1,11,12^. One crucial source of the confusion surrounding the nature of risk preference arises from its various operationalizations. Specifically, risk-preference assessment spans three measurement traditions that can be classified into broad categories of measures: propensity, frequency and behavioural measures (Table 1). These categories differ in several relevant ways. First, they fundamentally cover different aspects of risk: propensity measures aim to capture individuals’ attitudes towards risk, whereas frequency and behavioural measures aim to capture actual risky behaviour. Only behavioural measures typically eliminate differences in individuals’ opportunity to engage in risk by providing a standardized task to all respondents. Second, there are pragmatic or disciplinary differences in how measures from these categories were developed and applied. For example, behavioural measures have been the workhorse of risk research in economics, with its interest in capturing risk attitudes in the financial domain using incentivized measures. In turn, propensity and frequency measures have been adopted widely in psychology, covering a broader set of domains, including health, social and recreational risks. Considerable heterogeneity has been noted in the patterns and characteristics of measures, with only some showing desirable psychometric characteristics, such as reliability or predictive validity^13–16^. Crucially, past work suggests disagreement between different measures^1,13,17^. Resolving whether risk preference shares two central characteristics of a trait—namely, stability and coherence—is therefore impossible without acknowledging the central role of measurement. Obstructing clarity, however, is the piecemeal approach dominating past research; the adoption of single or few measures in any given study makes it difficult to obtain an overview across measures. Our work aims to help resolve this issue by taking a meta-analytic approach to investigate both the temporal stability and the convergent validity of extant measures of risk preference.

**Table 1:** Descriptions and examples of different categories of risk preference measures

Category	Description	Example
Propensity	Self-report measures; individuals indicate on an ordinal scale to what extent they identify as someone who likes or is willing to take risks in general or in specific domains.	Are you generally a person who is willing to take risks or do you try to avoid taking risks?^63^
Frequency	Self-report measures; individuals indicate on a scale or in an open field to what extent or how often they partake in activities in specific life domains.	How many times in the last seven days have you had an alcoholic drink?^13^
Behavioural	Behavioural measures; individuals are asked to decide between two or more options offering different (hypothetical or real) monetary gains and/or losses with varying probability. An index of risk preference is typically derived on the basis of a combination of choices or actions.	Mean number of pumps in a simulated balloon-pumping task^64^; percentage of risky choices in a lottery task^65^

Our first focus is quantifying the temporal stability of risk preference measures. This goal aligns with the key objective of discerning the sources of stability and change in human psychology and behaviour^18^, and mirrors existing research into other traits^19–22^. Although some studies in economics and psychology have probed the temporal stability of risk preference^2,12,23^, we note three gaps in existing research on measurement comparison. First, previous work found higher stability for propensity and frequency measures than for behavioural measures^2,13^ without fully considering the role of domain (for example, health or financial)^2^, causing an oversimplified picture of the stability of measures. Second, there is little consideration of how the stability of different psychological constructs varies across the lifespan^19,22^. Early life and young adulthood, marked by considerable biological, cognitive and social changes, usually show lower rank-order stability^24^, but past syntheses of the stability of risk preference did not account for age differences^2,23^. Third, previous research has not employed theoretically grounded models to analyse temporal stability patterns across different categories of measures, domains or populations, hindering comparison with other constructs (such as major personality traits) studied using formal models^19^. Understanding the lifespan trajectories of risk preference and their variation across domains is an important step to advance transactional theories of personality development^25^.

Our second focus is quantifying the convergent validity of risk preference measures. The issue of convergence is central to the goal of mapping theoretical constructs to specific measures, and many efforts in the behavioural sciences aim to empirically estimate these links^13,17,26^. It is also of practical importance because many studies investigating predictors or correlates of risk preference (for example, neuroimaging and genome-wide association studies^27–29^) often use only a single or limited set of measures to capture risk preference. To the extent that different measures disagree, these should not be used interchangeably and should be carefully selected to match the construct of interest. Previous work on risk preference reports a relatively low convergence between measures, although propensity and frequency measures may exhibit moderate convergent validity among themselves, whereas behavioural measures show comparatively low convergent validity, in terms of both observable behaviour and computational parameters^13,30^. We note three gaps in extant work on the convergent validity of risk preference measures. First, studies typically employ only a few different measures, limiting the extent to which an assessment of convergence between many measures can be performed in a single study. Second, the adoption of few measures in single studies often means that the moderating influence of measure (for example, category or domain) or respondent characteristics (for example, age) on convergence cannot be ascertained. Third, studies have been unable to assess the extent to which low convergent validity is a direct result of poor reliability of specific measures^31,32^.

This study tackles these outstanding gaps by examining the temporal stability and convergent validity of risk preference measures and adopting an individual participant data meta-analysis^33^. We conducted a systematic review to identify longitudinal datasets comprising different measures of risk preference, including propensity, frequency and behavioural measures. The curated database represents a dataset capturing 358 different measures of risk preference from 33 longitudinal panels, split into 57 different samples from 579,114 respondents. We also conducted a categorization of measures (for example, category and domain) and associated respondents (for example, age and gender). Equipped with these data, we conducted analyses for an overview of the temporal stability and convergent validity of risk preference measures.

First, to examine temporal stability, we performed a variance decomposition analysis providing a picture of the amount of variance that can be accounted for in temporal stability by measure-, respondent- and panel-related predictors. We further adopted a formal modelling approach using the Meta-analytic Stability and Change (MASC) model^19^ to capture the temporal stability of risk preference measures while distinguishing between domains (for example, investment, gambling, smoking and ethical). The MASC model distinguishes systematic variance from measurement error while capturing the potentially nonlinear nature of test–retest correlations over time and without strong assumptions about the functional form of its stability, with its parameters allowing for a wide range of functional forms. We further employed MASC to re-analyse longitudinal panel data for other pertinent psychological constructs, including personality and affect, providing a direct comparison between our results and those for other major psychological constructs.

Second, to examine convergent validity, we performed variance decomposition analysis to quantify to what extent measure-, respondent- and panel-related predictors account for the heterogeneity observed between intercorrelations. It has been suggested that the reliability of individual measures creates boundary conditions for their convergence^31^; thus, we consider measure reliability as a measure-related predictor in these analyses. We further report meta-analytic syntheses of the empirical relation across measures between and within category and domain pairs. We hope that by clarifying the two central characteristics of measures of risk preference—temporal stability and convergent validity— we will contribute to improving its measurement, describing its life-course patterns and, ultimately, increasing its utility as a construct in the behavioural sciences.

## Results

### Overview of the longitudinal data

**Fig. 1:**
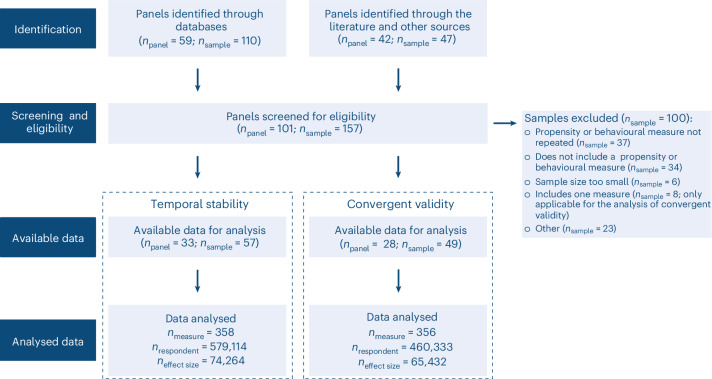
Systematic search for longitudinal samples. Flow chart of systematic search.

**Fig. 2:**
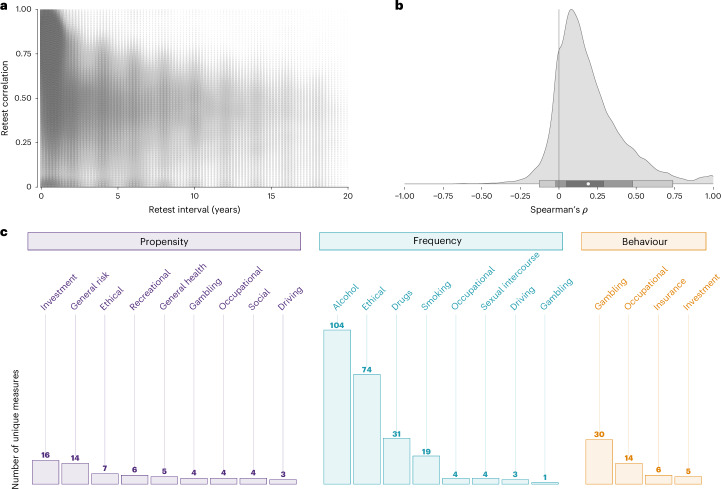
Overview of correlations and categorization of measures by domain. **a**, Two-dimensional density plot of test–retest correlations for all risk preference measures as a function of retest interval (number of correlations, *k* = 74,264). **b**, Distribution of all intercorrelations between risk preference measures (*k* = 65,432). The white dot represents the mean, and the shaded areas represent bootstrapped 95%, 80% and 50% confidence intervals. **c**, Number of unique measures split by category (propensity, frequency and behaviour) and domain.

Figure 1 shows the systematic approach we adopted to identify longitudinal samples suitable for estimating the temporal stability and convergent validity of risk preference measures. We distinguish between panels and samples because if panels included data from several countries, we treated these as separate samples to avoid confounding within- and cross-country differences. As per our inclusion criteria, all samples contained at least one propensity or behavioural measure. From the initially identified pool of 101 panels (157 samples), we included 33 panels (57 samples) that allowed computing test–retest information for at least one measure of risk preference, and 28 panels (49 samples) that allowed computing intercorrelations between two or more measures of risk preference. Finally, for each risk preference measure, sample, age group and gender, we calculated test–retest correlations between all measurement wave combinations for temporal stability analyses, and all possible intercorrelations between measures for convergent validity analyses. This process yielded 74,264 test–retest correlation coefficients for temporal stability and 65,432 intercorrelations for convergent validity analyses. As Fig. 2a shows, the test–retest correlations span a considerable range, with most data being available for short(er) retest intervals. Concerning intercorrelations between measures, Fig. 2b shows a wide range of correlations, with a mode in the small but positive range.

The dataset covers 358 different measures of risk preference spanning three measure categories (that is, propensity, frequency and behaviour). To achieve a fine-grained classification of measures lacking in the risk preference literature, we conducted a categorization of all measures, which yielded 14 measurement domains (for example, general health, financial, recreational and driving). Crucially, this categorization clarifies important differences across, as well as gaps between, the domains investigated in each category. As shown in Fig. 2c, although propensity measures capture most domains detected in our data (9 of 14), frequency measures capture a large but different subset of these (8 of 14). Behavioural measures, in contrast, capture only a small minority of finance-related domains, such as investment and gambling (4 of 14). Furthermore, we observed considerable heterogeneity in their composition: although the propensity and frequency categories include mostly one-item measures, the behavioural category includes predominantly multi-item (that is, trials) measures (Supplementary Fig. 1). This imbalance is ultimately due to the different traditions spanning the psychology, economics and public health literature that have investigated risk preference using different measurement strategies. Next, we provide an in-depth comparison of the measures’ temporal stability.

### Temporal stability

To obtain an overview of the temporal stability data, we visualized the number of measures by category and retest interval as well as a breakdown of the test–retest correlations by measure category (propensity, frequency and behaviour; Supplementary Fig. 2a). We noted substantial differences in the amount of data for the three categories, with most measures being classified as propensity or frequency measures, and only a minority as behavioural measures. The underrepresentation and overall shorter test–retest intervals for behavioural measures observed in our sample are products of there being overall fewer samples that have (repeatedly) included such measures in their assessment batteries, probably due to the additional burden of deploying behavioural measures that typically require extensive instructions, multiple choices and, potentially, incentivization. Supplementary Fig. 2b provides an impression of the distributions of retest correlations across time and measure categories, indicating considerable heterogeneity between measures, which we explore quantitatively in detail below.

#### Variance decomposition of test–retest correlations

Our main question concerns the relative contributions of measure, respondent and panel characteristics in accounting for patterns of temporal stability in different measures of risk preference. For this purpose, we adopted a Shapley decomposition approach, which estimates the average marginal contribution of different predictors to the variance in an outcome of interest^34^—here the test–retest correlations. We were particularly interested in the role of specific measure- and respondent-related predictors that have been either hypothesized or shown to account for some variance in temporal stability in past work on risk preference^13,35^ or other psychological constructs^19^. For measure-related predictors, we focused on category (that is, propensity, frequency or behaviour), domain (for example, general health or recreational), scale type (for example, ordinal or open-ended), the number of items per measure and the length of the test–retest interval (for example, six months, one year or five years). For respondent-related predictors, we considered age group, gender and the number of respondents. Finally, we included panel as a predictor to capture the role of unobserved panel characteristics (for example, the quality of data collection or data entry) that can influence test–retest reliability.

**Fig. 3:**
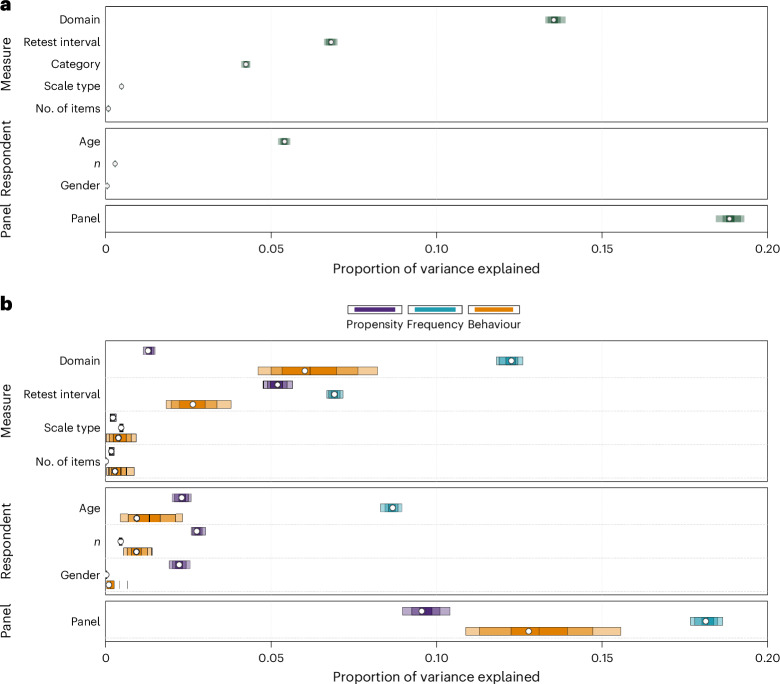
Variance decomposition of temporal stability. **a**, Relative contributions of measure-, respondent- and panel-related predictors to the adjusted *R*^2^ in regression models predicting test–retest correlations of all risk preference measures (*k* = 74,264). **b**, Relative contributions of measure, respondent and panel predictors to the adjusted *R*^2^ in regression models predicting test–retest correlations of propensity (*k* = 24,054), frequency (*k* = 48,536) and behavioural (*k* = 1,674) measures. In both panels, the dots represent the mean estimates, and the shaded uncertainty bands represent the 95%, 80% and 50% confidence intervals.

We conducted an omnibus analysis to assess to what extent measure, respondent and panel predictors explained differences across all test–retest correlations. Altogether, a model considering all predictors captures 49.8% of the observed variance. Figure 3a shows that a large portion of the variance could be explained by measure-related predictors, including domain (13.5%), category (4.2%) and retest interval (6.8%), but not much by scale type (0.5%) or number of items (<0.1%). We also found that some of the variance could be explained by respondent-related predictors, particularly age (5.4%). Finally, panel captured a large portion of the variance (18.8%), suggesting that a number of (unobserved) panel characteristics also contribute to systematic differences in the observed temporal stability of measures.

Given our focus on comparing measure categories, we further explored the differences between the contributions of these predictors to propensity, frequency and behavioural measures separately. These category-specific models explained 23.7%, 46.9% and 24.1% of the total variance for propensity, frequency and behavioural measures, respectively. The results are depicted in Fig. 3b. Four insights can be drawn from the comparison between measure categories. First, domain explained a noteworthy percentage of variance for frequency (12.3%) relative to propensity (1.3%) and behavioural (6.0%) measures. This suggests considerable heterogeneity within some categories as a function of domain (particularly for frequency measures), which we explore by analysing temporal trajectories by domain below. Second, retest interval contributed to more explanatory power for propensity (5.2%) and frequency (6.9%) measures than for behavioural measures (2.6%), suggesting that temporal patterns are less pronounced for the latter. Third, concerning respondent-related predictors, we found that age explained a considerable percentage of variance in the test–retest correlations, but particularly for frequency (8.7%) relative to propensity (2.3%) and behavioural (0.9%) measures. These results suggest some specificity regarding the effects of age by measure category. Fourth, as in the omnibus analysis, a number of (unobserved) panel characteristics seem to contribute to systematic differences between panels, but this effect is most pronounced for frequency measures.

#### Meta-analyses of temporal stability

We used the MASC model^19^ to capture the trajectory of test–retest correlations across measures of risk preference and compare these to other psychological constructs. MASC uses three parameters to represent different properties of temporal trajectories: reliability (the proportion of between-person variance excluding random error), change (the proportion of variance that is subject to changing factors) and stability of change (the rate at which change occurs over time).

**Fig. 4:**
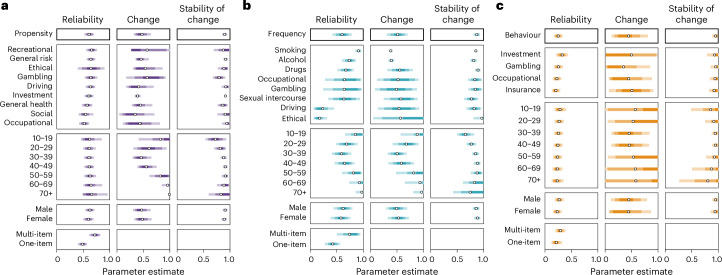
MASC model results for parameter estimates. **a**–**c**, Parameter estimates for propensity (*k* = 3,794) (**a**), frequency (*k* = 3,963) (**b**) and behavioural measures (*k* = 708) (**c**) of risk preference. The circles represent the mean estimates, and the shaded uncertainty bands represent the 50%, 80% and 95% HDIs.

Figure 4 shows the distributions of predictions for each of the model parameters, distinguishing further between domains (for example, recreational, general health, smoking and investment), respondent groups (age groups and gender) and number of items. We found a ranking in overlapping reliability estimates for the three measure categories, with the highest reliability found for propensity measures (mean, 0.61; 95% highest density interval (HDI), (0.52, 0.70)), followed by frequency measures (mean, 0.60; 95% HDI, (0.42, 0.78)) and behavioural measures (mean, 0.25; 95% HDI, (0.17, 0.34)). Crucially, relative to propensity and behavioural measures, the reliability of frequency measures varies widely by domain, with a wide range evident between the highest reliability for smoking (mean, 0.91; 95% HDI, (0.85, 0.96)) and the lowest for the ethical domain (mean, 0.18; 95% HDI, (0.06, 0.31)). In comparison, the ranges found for propensity measures, spanning from recreational (mean, 0.66; 95% HDI, (0.55, 0.76)) to occupational (mean, 0.52; 95% HDI, (0.42, 0.61)), and behavioural measures, spanning from investment (mean, 0.33; 95% HDI, (0.21, 0.44)) to insurance (mean, 0.21; 95% HDI, (0.13, 0.29)), are considerably smaller. Concerning the patterns of change and associated stability, the different measure categories and domains appear comparable, indicating some change but also long-term stability; this mimics patterns found in the temporal stability literature^18,19^.

**Fig. 5:**
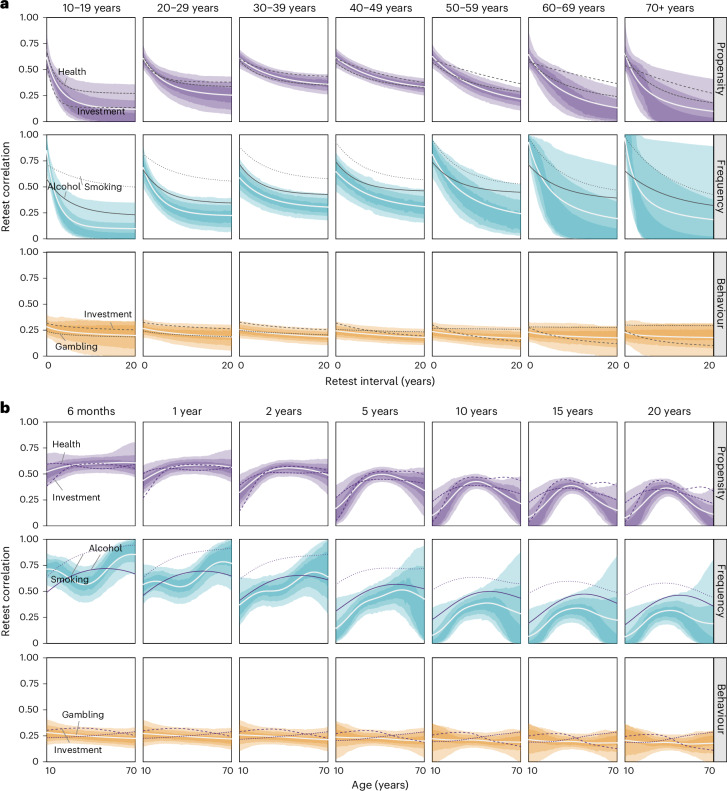
MASC model results for retest correlations. **a**,**b**, Predictions of retest trajectories given MASC parameters as a function of retest interval (**a**) and age (**b**) across all domains. The white line represents the mean, and the shaded uncertainty bands represent the 50%, 80% and 95% HDIs. The individual, annotated lines show the mean estimates for a selection of two domains per category.

Figure 5a shows the corresponding trajectories for predicted test–retest correlations as a function of retest interval (faceted for different age groups), particularly helpful for comparison with similar trajectories found for other psychological constructs^19^. Overall, we note that test–retest correlations are predicted to decrease substantially with longer retest intervals, yet this pattern is more pronounced for propensity and frequency measures than for behavioural measures. Although the rate of change varies with age (Fig. 4), this pattern applies across the lifespan.

Focusing on age effects, Fig. 5b shows the corresponding trajectories for predicted test–retest correlations as a function of age (faceted by retest interval). Consistent with past work using propensity measures of risk preference^35^ and major personality traits^22^, we note an inverse-U-shaped association between retest correlations and age, indicating that the temporal stability of propensity measures peaks in middle age. Notably, this pattern is observed for most domains captured by propensity measures (Supplementary Figs. 8–10). The overall pattern observed for frequency measures also approximates an inverse-U-shaped association, albeit with more heterogeneity between domains within this category. In particular, we found a clear inverse-U shape with age for alcohol consumption, drug consumption and smoking (Supplementary Figs. 11 and 12). For behavioural measures, we did not observe noticeable associations between temporal stability and age; this is reflected across the individual domains (Supplementary Fig. 13).

We did not identify any substantial differences concerning gender. This suggests that males and females show comparable stability trajectories across measures.

Finally, as expected, the results suggested that multi-item measures are considerably more reliable than single-item ones, suggesting this is an important factor concerning the heterogeneity in the temporal stability of risk preference measures.

We were also interested in assessing where risk preference stands relative to other constructs by comparing its temporal stability to that of personality, life satisfaction, self-esteem and affect using data from a previous review^19^ of self-report measures of these constructs (Supplementary Fig. 14). Our results suggest comparable, but somewhat lower, average stability of risk preference as captured by propensity and frequency measures relative to major personality constructs (for example, the Big Five and self-esteem). The largest difference is observed for behavioural measures, with considerably lower reliability than all other constructs considered, including affect (Supplementary Fig. 15).

The results on temporal stability support the notion that different risk preference measures show markedly different temporal stability signatures. Next, we explore further differences between measures by evaluating their intercorrelations.

### Convergent validity

#### Variance decomposition of correlations between measures

**Fig. 6:**
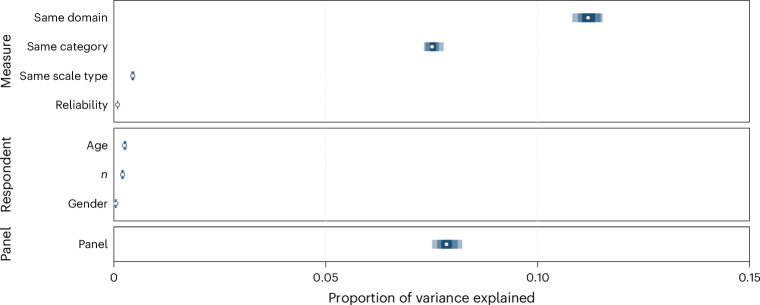
Variance decomposition of convergence between measures. Relative contributions of measure-, respondent- and panel-related predictors to the adjusted *R*^2^ in regression models predicting intercorrelations between measures of risk preference (*k* = 65,432). The dots represent the mean estimates, and the shaded uncertainty bands represent the 95%, 80% and 50% HDIs.

To estimate what proportion of variance in intercorrelations between risk preference measures could be explained by measure-related, respondent-related and panel predictor variables, we used the same approach as for the test–retest correlations (for details, see Methods). The variance decomposition analysis suggests that a model considering all predictors captures 27.6% of the variance in intercorrelations. More substantively, as shown in Fig. 6, the variance decomposition analysis suggests that category and domain play a considerable role: more than half of the explained variance was accounted for by whether or not the pair of measures matched in terms of category (7.5%) and domain (11.2%). We also found that measure reliability accounted for less than 1% of the variance, indicating little support for poor reliability of risk preference measures being the main driver of their (lack of) convergence. Finally, respondent-related effects offer little to no contribution, while panel characteristics seem to account for some amount of variance, suggesting that unobserved panel characteristics capture relevant, systematic variance in the correlation between measures. In sum, the variance decomposition analysis suggests that measure characteristics, specifically, category and domain, capture important aspects of measure convergence. Next, we provide a more detailed overview of the role of these factors by providing a meta-analytic correlation matrix across pairs of measures that distinguishes between category and domain.

#### Meta-analyses of convergent validity

**Fig. 7:**
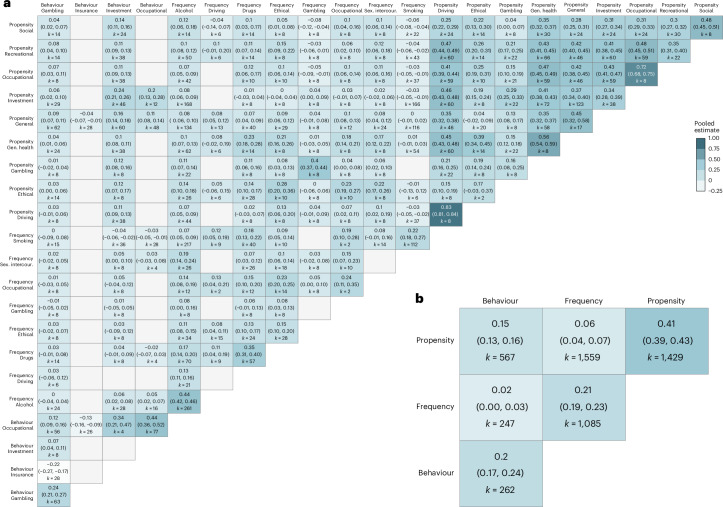
Meta-analytic correlation matrices. **a**,**b**, The matrices depict the results of the meta-analyses of intercorrelations between measures of risk preference (*k* = 5,149), with each cell representing the meta-analytic result for the specific measurement pair of measure domains (**a**) or measure categories (**b**). Empty cells (grey) are due to the lack of data availability to estimate the respective correlation. In each cell, the top number represents the correlation, while the values in parentheses show the 95% HDI.

We conducted separate meta-analyses at different levels of aggregation to map out the convergent validity of risk preference measures across categories and domains. A meta-analysis across all available intercorrelations suggests an average meta-analytic intercorrelation of 0.17 (95% HDI, (0.14, 0.19)). However, this value hides considerable heterogeneity. Figure 7a shows that across pairs of categories and domains, we observe a large range of intercorrelations, from around −0.2 to circa 0.8. The meta-analytic correlation matrix also shows evidence of overall higher average correlations along the diagonal, signalling that matching both category and domain leads to typically higher intercorrelations than matching only across domains or categories. Importantly, as can be seen in Fig. 7b, when considering aggregation at the category level, there is a clear ranking of the average intercorrelations within each category, with this being the highest for propensity (mean, 0.41; 95% HDI, (0.39, 0.43)), followed by frequency (mean, 0.21; 95% HDI, (0.19, 0.23)) and behavioural measures (mean, 0.20; 95% HDI, (0.17, 0.24)). Finally, and more importantly, there is evidence of little convergence between categories, with cross-category meta-analytic correlations being around or smaller than 0.1. As a robustness check, we conducted additional meta-analyses where all behavioural measures fall within same (financial) domain and obtained comparable results (Supplementary Information).

When the results on both temporal stability and convergent validity are considered jointly, different risk preference measures can show very different psychometric signatures, including patterns of temporal stability and convergent validity. This supports the notion that measurement issues challenge clarity concerning the nature of the construct.

## Discussion

Approaching the ongoing debate about whether risk preference represents a stable and coherent trait from a measurement perspective, we curated a collection of previously underutilized longitudinal samples, yielding data for 358 measures of risk preference covering three broad categories—propensity, frequency and behavioural—and covering various life domains. In analysing this resource, we provide a meta-analytic synthesis of the trajectories of temporal stability across measure categories while accounting for various measure (for example, domain and item number) and respondent (for example, age) characteristics. We were also able to contrast the temporal stability of different measure categories to those of prominent self-report measures of other psychological constructs such as personality and affect. Finally, we estimated the convergent validity across measures of risk preference.

Our temporal stability results revealed variations in reliability across the three measure categories. Propensity and frequency measures showed the highest temporal stability, with values similar to but somewhat lower than those for other major personality traits as captured through self-report^19^. In comparison, behavioural measures of risk preference showed considerably lower stability, with reliability below that of the other categories (propensity and frequency), personality traits and affect. Concerning the role of age, test–retest correlations for propensity measures showed an age-related (inverted-U) trend similar to those found for major personality traits^19,22,24^. In turn, age patterns for frequency measures varied considerably across domains, indicating distinct pathways for age-specific versus lifelong trajectories of different behaviours^36,37^; some domains, like smoking and alcohol consumption, resembled the patterns found for propensity measures, while others, like driving and ethical behaviour, showed overall lower stability and more pronounced changes in young adulthood and midlife. Unlike propensity and frequency measures, behavioural measures did not capture any lifespan trends or show large domain-specific differences across the domains considered, which were mostly of a financial nature (for example, investment, gambling and insurance). These results suggest that different measurement traditions are characterized by distinct temporal, domain and age-related trajectories, emphasizing the important role of measurement in establishing the empirical patterns associated with the risk preference construct.

Our convergent validity analyses showed low overall convergence between risk preference measures, revealing considerable heterogeneity among measure categories. Propensity measures demonstrated the highest convergence, while frequency and behavioural measures exhibited lower convergence, aligning with results from past studies^13,17^. Notably, this was the case even though propensity measures encompassed a broader range of domains (for example, health, occupational and gambling), particularly compared with behavioural measures, which focused primarily on financial domains (for example, investment, gambling and insurance). Similar to the temporal stability analyses, the convergent validity results underscore the important role of measurement tradition and raise questions about the coherence of the risk preference construct when captured by distinct measure categories.

We discuss three main implications of our findings for current theorizing and research on risk preference. Foremost, our results show that we must invest new energy into developing measurement frameworks to explain the observed convergence and divergence across measures. One explanation may be that different measures capture fundamentally different aspects of risk^38^. Whereas propensity measures aim to capture individuals’ attitudes towards risk, frequency and behavioural measures aim to capture actual risky behaviour. From this viewpoint, the gap between propensity and other measures could be considered a special case of the classic intention–behaviour gap. However, the observed differences between frequency and behavioural measures indicate that more is at play. Indeed, there are other ways in which these measure categories differ. One involves the modality of assessment, as both propensity and frequency measures rely on self-report. From this perspective, the higher alignment between these two categories and, more generally, personality traits measured with the use of self-reports is less surprising. However, the gaps between frequency and propensity measures must also be explained. One source of differences may stem from frequency measures capturing not only individuals’ appetite for risk but also other factors, such as the opportunity to engage in these risks (for example, car ownership increases the opportunity for risky driving) or processes that go beyond normal variation in preferences and include pathological behaviour and addiction (for example, antisocial behaviour and alcoholism). Regarding the overall lower reliability and convergence of behavioural measures, behavioural measures of risk preference are typically conducted in lab settings using incentive-compatible tasks, which may create ‘strong’ contexts (that is, highly structured situations) that overpower individuals’ tendencies^31^. Further limitations include the possibility of contamination by factors not directly related to risk preference (for example, numeracy and risk literacy) and the need for numerous trials to reliably estimate latent traits, which is more easily accomplished by integrating behavioural episodes from memory as done in propensity and frequency measures^39^. More generally, the level of granularity varies substantially between measures; propensity measures cover broader domains (for example, ‘health’) and time frames (for example, ‘in general’), frequency measures are more concrete (for example, ‘number of cigarettes’) and time-constrained (for example, ‘in the last 30 days’), while behavioural measures are yet more specific. This discrepancy can reduce reliability, as individuals interpret questions differently or provide varied answers based on different cues on any given occasion^40,41^. Understanding how these various factors contribute to measurement gaps is not merely of methodological relevance but central for achieving conceptual clarity^42^. While it may be too soon to make a final assessment about the theoretical status of the risk preference construct, our results suggest that it will be crucial to integrate conceptual aspects of risk preference into a more coherent set of measurement strategies similar to work in other areas of human personality^17,43,44^.

The second implication is that, from a developmental theory perspective, our results emphasize the need to connect the temporal stability of risk preference with lifespan changes in various contexts and, importantly, domains. Many extant theories make valuable contributions to explaining the complex nature of stability and change in personality traits^25^ and behaviours, such as antisocial^36^ or health behaviours^37^. In particular, transactional models^25,45^, focusing on the interplay between individual characteristics and environmental factors in determining phenotypic change across the lifespan, could be helpful in reconciling the idea of stable individual risk preferences with differential patterns across domains that are shaped by changing affordances and goals^46^ as well as individuals’ life experiences^47^.

Third, our results suggest that researchers should prioritize measure validation and development in future work on risk preference. Regarding validation, we should strive for more comprehensive comparisons of existing measures by conducting more primary studies into un(der)explored measure categories, domains and their combinations^48^, by targeting specific domains using multiple measures from several categories (such as risky driving)^49^. Regarding measure development, recent technological development suggests that new forms of measurement could anchor risk preference measures in more real-world experience—for example, through the use of virtual reality^50^ and other advances in computational methods for personality assessment^51,52^.

We note three limitations as well as future extensions of our work. First, our dataset has limitations as it captures a large but not exhaustive set of measures and data on risk preference. For example, focusing on temporal stability led us to focus on longitudinal designs, but this is not strictly necessary for convergent validity analyses, which could be expanded by including cross-sectional data not available to us due to our inclusion criteria. Similarly, we meticulously coded and analysed measure (for example, category, domain, test–retest interval and item number) and respondent characteristics (for example, age and gender). Yet, other factors (for example, intelligence and socio-economic status) could also be relevant^53^. Future work may pursue more comprehensive efforts by leveraging coordinated analyses across multiple teams to enhance the mapping of risk preference across larger sets of measures and data sources.

Second, our workflow involved several analytical choices, including the categorization of measures into domains, the preprocessing of covariates and the selection of model priors, that have the potential to impact some of our conclusions. However, we aimed to reduce or estimate the impact of these choices by making principled decisions informed by past work, conducting multiverse analyses to assess result robustness whenever possible, and making all scripts publicly available to foster scrutiny and allow future collaborative research on risk preference.

Third, and crucially, although temporal stability and convergent validity are fundamental properties of measures, another important (albeit not entirely orthogonal) property is their predictive validity. Past studies provide support for the predictive validity of some self-report measures^2,16^, but there is overall a dearth of such studies in the risk preference literature. We envision that future many-labs prediction studies as well as individual participant data meta-analyses could support such efforts. Future work should particularly aim to include the prediction of more objective measures spanning different domains, such as health (for example, hospital visits), investment (for example, stock portfolios) or ethics (for example, arrest records), to establish a ground truth for the predictive value of different risk preference measures across real-life outcomes.

To conclude, our results suggest that despite considerable advances in the measurement of risk preference, existing measurement strategies do not paint a coherent picture of individuals’ risk preferences and lifespan trajectories. Future work should consider these results to develop better theories of lifespan development and realize the promise of risk preference as a construct to help understand, predict and intervene on important life outcomes, ultimately contributing to individuals’ health, wealth and happiness.

## Methods

### Identification of samples

Our analysis protocol was not preregistered, but we adopted a systematic method to find longitudinal data that include measures of risk preference (Fig. 1). We started by identifying longitudinal panels by (1) performing searches on general-purpose search engines, survey listings and data repositories (that is, Google Database, Gateway to Global Aging Data, Gesis, IZA, ICPSR, CNEF and UK Data service) using relevant terms (for example, ‘risk preference’, ‘risk aversion’, ‘risk attitude’, ‘take risks’, ‘survey’, ‘panel’ and ‘longitudinal’; see Supplementary Table 1 for a list of our search terms), (2) consulting past literature for references to longitudinal panels or studies that have estimated the temporal stability of psychological constructs^2,19,23,54,55^, and (3) submitting informal requests to colleagues for suggestions concerning panels or specific studies. This search led to identifying 101 longitudinal panels (157 samples; Supplementary Table 2). It is important to note that we differentiate between panels and samples, such that samples have their origin in a panel. For example, if a panel (for example, SHARE) included data from multiple countries (for example, SHARE-Switzerland, SHARE-Germany and SHARE-Belgium), we treated the latter as distinct samples to prevent confusion between differences within and across countries. To determine the relevance of each of the 157 samples for our analyses, we adopted a set of screening criteria (Supplementary Table 3). In brief, we included a sample in our analyses if it (1) was publicly available, (2) included data on at least one consistently formatted propensity or behavioural measure of risk preference with responses from the same respondents across at least two time points, and (3) included data on the gender and age of the respondents. This procedure led to the creation of a dataset comprising 33 longitudinal panels containing 57 samples (Supplementary Table 4). For each sample, we included data that were available as of May 2023. We did not conduct an assessment of the risk of bias or quality of the included samples due to the lack of standard and established tools for evaluating open datasets of observational research^56^.

### Categorization of measures

To further characterize the newly curated dataset, we conducted a categorization of each risk preference measure. The following measure characteristics are particularly relevant to our analysis: measure category (propensity, frequency or behaviour), domain (for example, investment, general health, social or recreational), scale type (for example, open or closed questions) and the number of items per measure. Supplementary Table 5 presents descriptions of risk preference measures that are representative of the variety of measures included in the samples used for our analyses. With regard to the domains captured by different risk preference measures, we included measures covering as many domains as possible—that is, we did not exclude measures in prespecified domains. Furthermore, we adopted a bottom-up, data-driven approach to distinguish between domains. We felt that this approach was best suited for our purpose, as this allowed us to (1) scope extant work and systematically identify the domains most commonly assessed in the risk preference literature, and (2) provide an assessment of temporal stability and convergent validity while systematically investigating the role of domain at a high level of granularity. Overall, we identified 14 domains: alcohol, driving, drugs, ethical, gambling, general health, general risk, insurance, investment, occupational, recreational, sexual intercourse, smoking and social. Our labelling scheme has considerable overlap with terminology commonly used to group contexts or situations within which risk-taking can occur, although it makes fine-grained distinctions within domains, such as distinguishing between smoking or alcohol consumption and a more general health domain. We provide additional detail concerning an assessment of measure characteristics in the Supplementary Information.

### Temporal stability

In what follows, we give an overview of the steps involved in computing test–retest correlations, conducting variance decomposition of test–retest correlations and modelling temporal stability using the MASC model^19^. We provide additional information concerning each step in the Supplementary Information.

#### Computing correlations

To compute test–retest correlations, we followed past approaches^19,21^. For each panel, we included the data from all the respondents, regardless of whether or not they provided responses on all measurement waves. Within each sample and for each risk preference measure, we calculated test–retest correlation coefficients for each possible wave combination. For example, for a sample with Waves 1, 2 and 3, we calculated three sets of test–retest correlations: between Waves 1 and 2, between Waves 2 and 3, and between Waves 1 and 3. More importantly, we computed test–retest correlations separately for females and males as well as for respondents of different age groups (defined by binning age at the time of the first data collection point into ten-year bins).

Robustness checks^21^ suggested high correlations between test–retest correlations computed using different metrics and using (non-)transformed data (Supplementary Figs. 3 and 4). Consequently, we report the results using Pearson’s *r* correlation coefficients for non-transformed data. To obtain reasonable estimates, test–retest correlations calculated from fewer than 30 responses were excluded from the main analyses. Furthermore, we restricted the dataset to correlations with a retest interval of up to 20 years. This resulted in a set of 74,264 test–retest correlations.

#### Variance decomposition

To estimate the proportion of variance in the 74,264 test–retest correlations that could be explained by measure-related, respondent-related and panel predictor variables, we used Shapley decomposition^34^. First, we obtained the adjusted *R*^2^ value from each of the 2^9^ subsets of linear regression models (2^8^ regression models for the category-specific variance decomposition). Second, we estimated the variance explained by each predictor by calculating the weighted average change in adjusted *R*^2^ resulting from its inclusion in the model. Third, using 100 resampled datasets, we generated 100 bootstrapped estimates for each prediction, from which we computed bootstrapped confidence intervals^57^.

#### MASC model

##### Model description

The MASC model is a nonlinear model proposed to capture the trajectory of test–retest correlations over time^19^. In this model, the test–retest correlation *r*_*t*2−*t*1_ at a specific time interval is a function of the proportion of reliable between-person variance, rel; the proportion of this reliable variance explained by changing factors, change; and the stability of these changing factors over time (per year), stabch. This is formalized as *r*_*t*2−*t*1_ = rel × (change × (stabch^time^ − 1) + 1).

Supplementary Fig. 5a describes the model, and Supplementary Fig. 5b illustrates how different model parameterizations alter the shape of the curve.

##### Aggregation of test–retest correlations

To minimize potential convergence issues that arise from meta-analysing 74,264 test–retest correlations using MASC, we aggregated the test–retest correlations. We obtained these aggregates by first grouping the test–retest correlations by sample, measure category, domain, item number and retest interval, as well as respondent gender and age group. We then calculated the average test–retest correlation for each of these groupings, using inverse-variance weighting and accounting for the dependency between these correlations. This resulted in 8,465 aggregated correlations.

##### Bayesian model specification

We set up the MASC model such that for each parameter (that is, rel, change and stabch) we accounted for the effects of domain, linear age, quadratic age and gender, as well as the interaction between linear and quadratic age and domain. We also included item number as a fixed predictor and sample as a random factor for the rel parameter. Importantly, to obtain meta-analytic estimates, we additionally specified the (aggregate) standard errors of each correlation. Lastly, to best capture domain-specific effects within each category, we fitted the model separately for each measure category using their respective aggregated retest correlations and aggregated standard errors.

To estimate the parameters of this nonlinear hierarchical model, we used a Bayesian approach to account for the large differences between sample sizes and retest intervals encountered in this set of data sources. We specified weakly informative priors on the model parameters and hierarchical standard deviations to include values reported previously in the literature^2,13,19^.

The analyses were conducted in the R statistical environment version 4.4.1 (ref. ^58^) using the brms package version 2.22.0 (ref. ^59–61^), which provides a high-level interface to fit hierarchical models in Stan^62^.

##### Construct comparison

To compare the temporal stability and reliability of risk preference to that of other psychological constructs (for example, personality), we re-analysed the set of correlations included in a previous review^19^ using a Bayesian estimation procedure and a set of MASC model specifications to maximize comparability to the analyses conducted for risk preference.

### Convergent validity

In what follows, we give an overview of the main steps involved in computing intercorrelations between measures, variance decomposition of intercorrelations and the meta-analyses of convergent validity. We provide additional information concerning each step in the Supplementary Information.

#### Computing correlations

For the assessment of the convergence of risk preference measures, we started with the set of samples used to assess the temporal stability of risk preference but selected only those samples that included two or more measures of risk preference within at least one wave, and for which the same set of respondents had provided answers. As a result, we conducted our convergent validity analyses on 49 samples from 28 panels (Fig. 1), retaining the same three measure categories and 14 domains used in the temporal stability analyses. First, for each sample, we computed the correlations between every possible pair of measures within the same data collection point. We computed these correlations separately for females and males as well as respondents of different ages. We excluded intercorrelations computed from the responses of fewer than 30 respondents. This resulted in a dataset of 65,432 intercorrelations. Robustness checks^21^ suggested high correlations between intercorrelations computed using different metrics and using (non-)transformed data (Supplementary Figs. 6 and 7). Here we report results using Spearman’s *ρ* correlation coefficients for non-transformed data, which were based on a minimum of 30 responses.

To avoid model convergence issues when running the meta-analysis, we grouped the intercorrelations (for example, by type of pair, age, gender or panel) and then aggregated the intercorrelations within these groupings, resulting in 5,149 aggregated intercorrelations.

#### Variance decomposition

We first obtained an overview of the convergent validity data by visualizing the distributions of intercorrelations of measures separately for different measure pairs (Supplementary Fig. 17). The resulting pattern speaks to the large heterogeneity in correlations between measures as well as possible differences between and within measure categories. Similar to our approach for test–retest correlations, we used variance decomposition to provide a quantitative summary of intercorrelations as a function of several measure and respondent-related characteristics, as well as panel. Specifically, concerning measure characteristics, we included dummy-coded predictors to code for the matching (for example, propensity–propensity) or mismatching category (for example, propensity–frequency), domain and scale type. Furthermore, using the results from the temporal stability analyses above, we computed the average reliability of each pair of measures and included this in our predictors to assess the extent to which measures’ reliability contributes to their convergence. We obtained the adjusted *R*^2^ value from each of the (2^8^) models, estimating the variance explained by each predictor by calculating the weighted average change in adjusted *R*^2^ resulting from its inclusion in the model, and using a bootstrapping procedure to compute confidence intervals.

#### Meta-analysis

To obtain the overall meta-analytic estimate of the convergence of risk preference measures, we first fitted a Bayesian hierarchical intercept-only model. Second, to obtain meta-analytic estimates for the convergence between specific pairs of measure categories and domains, we fitted Bayesian hierarchical (robust) regression models that included a predictor coding for the different types of measure pairs.

### Multiverse analyses

We conducted a series of multiverse analyses with alternative datasets resulting from different data preprocessing and various alternative analytic choices. We found overall qualitatively similar patterns of results across the multiverse of choices considered. We provide additional details concerning these analyses and results in the Supplementary Information.

### Reporting summary

Further information on research design is available in the Nature Portfolio Reporting Summary linked to this article.

## Supplementary information

Supplementary Information Supplementary Figs. 1–17, Tables 1–5 and text.

Reporting Summary

## Data Availability

We analysed data from existing studies and panels. All the data are made publicly available through the original data sources and need to be accessed by following the providers’ data access policies (see Reporting Summary for the URLs). We also provide a detailed overview of the data and analysis in a companion website (https://cdsbasel.github.io/temprisk/), and a minimum dataset with the estimated test–retest correlations and intercorrelations from the primary data sources is available in an online repository (https://osf.io/5kzgd/).

## Code availability

We have made the data processing and analysis scripts publicly available in an online repository (https://osf.io/5kzgd/).
