# Supplementary material for: A systematic review and meta-analyses of the temporal stability and convergent validity of risk preference measures
Source: Nat Hum Behav. 2025 Jan 27;9(4):700–12. doi: 10.1038/s41562-024-02085-2 (PMC12018263; doi:10.1038/s41562-024-02085-2)
Supplement: Supplementary file 1 — Supplementary Figs. 1–17, Tables 1–5 and text. [file 41562_2024_2085_MOESM1_ESM.pdf]

nature human behaviour

Supplementary information https://doi.org/10.1038/s41562-024-02085-2

# A systematic review and meta-analyses of the temporal stability and convergent validity of risk preference measures

In the format provided by the authors and unedited

# Supplementary Information

## Contents

Identification of Samples . . . 3
Categorisation of Measures . . . 3
Data Pre-Processing . . . 4
Dataset Information . . . 4
Risk Preference Measures . . . 4
Variable recoding based on question dependency. . . . 4
Reverse coding. . . . 5
Composite measures. . . . 5
Harmonising variable names . . . 5
Sample Demographics . . . 5
Data Processing . . . 6
Temporal Stability . . . 6
Computing test–retest correlations . . . 6
Aggregating test–retest correlations . . . 8
Convergent Validity . . . 9
Computing intercorrelations . . . 9
Aggregating intercorrelations . . . 9
Analysis . . . 10
Temporal Stability . . . 10
Variance decomposition . . . 10
Meta-Analytic Stability and Change model (MASC) . . . 11
Re-analysis of the Anusic & Schimmack [10] dataset . . . 14
Convergent Validity . . . 16
Variance decomposition . . . 16

Meta-analyses . . . . . . . . . . . . . . . . . . . . . . . . . . . . . 17
Multiverse Analyses . . . . . . . . . . . . . . . . . . . . . . . . . . . . . . . 19

## Identification of Samples

To find as many longitudinal panels and associated samples with risk preference measures, we devised a list of search terms related to risk (e.g., risk*, gambl*, smok*, gambl*; Table 1). This list reflects the definition of risk from the economics and psychology literatures and covers many different areas of life. It was developed by consulting the questionnaires of multi-measure studies [1–6] as well as previously identified longitudinal samples (e.g., SOEP, USOC). As presented in the main paper, this search led us to identify a large number of panels (101) and associated samples (157) (Table 2), which we checked for possible inclusion in our study. We excluded sample or measures from our study using a clear set of inclusion/exclusion criteria (Table 3). Each sample was documented differently, thus, whenever available, we used the computerised (online) variable search engine to search for the risk-related terms, otherwise, we manually searched the codebooks and/or questionnaires available. Our systematic approach to search and screening resulted in the inclusion of 57 unique samples from 33 panels (Table 4).

## Categorisation of Measures

We conducted extensive coding and categorisation of each risk preference measure that met our inclusion criteria. Specifically, we coded the following information: the name of the panel it originated from, the measure category (i.e., propensity, frequency, or behaviour), the domain (e.g., recreational, smoking), the type of scale used (i.e., ordinal, discrete, composite or open ended) and, if ordinal or discrete (with a clear range of possible response values), the number of options or points in the scale. In addition, we included information that was specific to each category of risk preference measure. Specifically, for frequency measures, we specified the number of days over which a certain behaviour had to be reported (e.g., Over the last week/month/year how many times were you intoxicated?). For behavioural measures, we recorded whether the decision was incentivised or hypothetical [cf., 7]. Please note that we do not include these category-specific characteristics in our analyses because they are not instrumental to the comparison

between categories. Nevertheless, we provide this categorisation for completeness and future possible uses of these data that control for or examine the role of such characteristics. Overall, we identified 358 unique measures stemming from 57 longitudinal samples. We provide a detailed definition, coding and description of each type of measure in Table 5, as well as a complete list of the risk preference measures in the main code book available in the online repository.

## Data Pre-Processing

Prior to computing test–retest correlations, we pre-processed the data from each sample to create homogeneous datasets with regards to the dataset information, risk preference measures, and sample demographics. We provide details concerning each step below.

### *Dataset Information*

From each dataset, we extracted the wave identifiers and data collection dates (i.e., day-month-year). If these dates were missing, we determined for each wave a standard date by referring to the sample's data collection timeline and choosing the half-way point (e.g., if data collection took place between January and June of 2020, the 15th of March 2020 was selected as the date). In the case that only the year could be retrieved, we set June 15th as the default day. If the data collection date was missing for certain respondents within the wave of a panel, this date was filled by the mean of the available dates.

### *Risk Preference Measures*

**Variable recoding based on question dependency.** Depending on the design of the questionnaires/interviews, for some samples, respondents were not asked certain questions because of their response to previous (filter) questions. This was particularly the case for frequency measures. For instance, if an individual answered the question *"Are you currently a smoker?"* with *"No"*, the follow-up question *"How many cigarettes a day do you smoke?"* would not be asked and would automatically receive a "missing" or "not applicable" code. By ignoring dependencies between questions, valuable information on the

consistency of an individual's behaviour is missed, as instances of when behaviours might be interrupted and taken up again (e.g., quitting/taking up smoking) are unaccounted for. To deal with this, for each sample, we took into account responses to filter-type questions and replaced invalid/missing codes in subsequent related questions by an appropriate response. In the case of the above example, for all the participants who answered *"No"*, we replaced the invalid or missing code for the number of cigarettes smoked in a day with a *"0"* or *"None"*. To make such replacements possible, we only included measures in our analyses that had scales that offered the possibility of a 0 value or Never/None answer (Table 3).

**Reverse coding.** Whenever appropriate, we reversed the scales of measures such that higher values corresponded to greater risk-taking.

**Composite measures.** We define a composite measure as a measure which represents an index of risk taking that is calculated by combining two or more individual risk preference measures. This was particularly the case for behavioural measures. If a composite measure was not available in the raw dataset of the sample, we aggregated the set of available single responses using similar methods as that of studies with comparable tasks (e.g., proportion of risky choices). We provide a description of how these have been calculated for specific measures in the risk preference measure code book.

**Harmonising variable names.** We standardised the names of the measures such that the same risk preference measure (or highly similarly worded measure with the same response format and scale) included in different samples shared the same variable name.

### *Sample Demographics*

We recorded the age and gender of each respondent. Age was calculated at the time of each data collection point. If the respondent's birth year was available in the dataset, we used that to calculate their age, if not, we used the value of the pre-computed age in the dataset. Further, if only age group or age range information was available (e.g., 20-30), we defined age as the midpoint value (e.g., 25). Only data from respondents between the ages of 10 and 90 years were included in the analyses. We coded gender as a binary variable (0

= male and 1 = female). For data quality purposes, we did not include in our analyses the responses of respondents whose year of birth, age (i.e., if the age difference and time difference between first and last wave of participation differed by more than 2 years) or gender was inconsistently reported across waves. Additionally, if either the year of birth, age or gender was missing and could not be retrieved or estimated based on previous waves, the respondent was excluded from the analyses.

## Data Processing

### *Temporal Stability*

**Computing test–retest correlations.** To address our main research objectives, for each panel and risk preference measure, we calculated for all possible wave combinations test–retest correlations (Figure 2). Correlations were calculated separately for females and males of different age groups. We computed separate sets of test–retest correlations for different age group configurations: 5, 10 and 20-year age bins. Akin to Enkavi *et al.* [3], we estimated test–retest correlations using three different metrics: Pearson's r, Spearman's rho and intra-class correlations (ICC(2,1)). The correlation between these different metrics ranged between 0.68 and 0.99 (Figure 3). Further, the response distributions of some measures were highly skewed, thus we additionally computed test–retest correlations using log-transformed data. As shown in Figure 4, these were highly correlated with the test–retest correlations computed using the non-transformed data (r = 0.92 - 0.99). As a consequence, we report our main results using the Pearson's r correlation coefficient for the non-transformed data. Furthermore, when computing the test–retest correlations we obtained negative estimates (3.93% of the dataset used for analysis); for ease of interpretation, we replaced these values with zeroes prior to any analysis or aggregation procedures [cf., 3].

***Additional metrics.*** In addition to these correlation metrics, for each test–retest correlation coefficient we recorded the following (variables with an asterisk were included in our main analyses, the rest were included for data quality assessment and data

exploration):

- Respondent information: sample size, maximum age, minimum age, mean age*, median age, standard deviation of age, proportion of female respondents*, proportion of sample lost between the first and second data collection point (i.e., attrition rate)
- Retest interval: minimum, maximum, mean*, median and standard deviation of the number of years between the first and second data collection point
- Response properties: the coefficient of variation and skewness of the responses at both time points

When calculating the time interval between the first and second data collection point, we noted that for panels that collected data for different surveys simultaneously (e.g. American Life Panel), not all respondents completed the surveys in the same order; some respondents would complete a more recent survey prior to an older survey (based on the mean data collection date), resulting in a negative retest interval. Therefore, for a very small number of correlations (0.17%) the minimum retest interval was negative. However, in our analyses we use the mean time difference between waves (or surveys), which minimises this issue. One exception to this concerns the German Socioeconomic Panel, which in 2020 launched a COVID-specific survey in which data collection overlapped with the 2020 core survey. We could not adequately order this pair of waves (i.e., 2020-core and 2020-covid) as we systematically had correlations that either had a negative mean or median retest interval. Therefore, we excluded correlations that from this specific pair of waves.

***Sample size.*** Simulation studies have shown that large sample sizes may be needed to compute stable correlation coefficients [8]. On the companion website we show how the number of correlation coefficients in the dataset varies for different age groups based on different minimum sample size thresholds. For some age groups a substantial number of coefficients are lost as the threshold increases. To avoid losing valuable

information for certain age groups, we retained the set of test–retest correlations that had a sample size of at least 30 with age groups organised in 10-year bins. In line with the multiverse approach [9], the companion website provides an overview of the outcome of our analysis obtained using the different minimum sample size thresholds, age bins, and other processing steps.

**Aggregating test–retest correlations.** Given the high number of test–retest correlations in our dataset (N = 74,264 correlations), it was too complex and computationally intensive to use such a dataset to estimate the Meta-Analytic Stability and Change model (MASC; Anusic & Schimmack [10]) and adequately capture the trajectories of the correlations over time without encountering severe model convergence issues. Therefore, prior to fitting the MASC model, we aggregated the correlations. However, prior to aggregating the correlations, given that MASC model predictions are bounded between 0 and 1, we set any negative retest correlation (3.93%) to zero and thereafter we followed the process depicted in Figure 16:

1. First, we transformed each Pearson's r correlation coefficient into Fisher's z, and calculated the corresponding sampling variance.

2. Second, we grouped the test–retest correlations by panel, measure category, measure domain, item number, 3-month retest interval, gender, and age group.

3. Third, for each grouping we computed a synthesised estimate by aggregating test–retest correlation coefficients while accounting for the dependency between them as these were computed from the same set or subset of respondents [11]. For this purpose, we used inverse-variance weighting and set the correlation of the sampling errors within subsets to .5.

4. Lastly, these aggregated correlations and their standard errors were back transformed to Pearson's r.

This process resulted in 8,465 aggregated test–retest correlations being calculated.

## *Convergent Validity*

**Computing intercorrelations.** Samples which contained only one measure of risk preference were excluded from these analyses (n = 8). For each of the remaining samples and waves, we calculated correlations between the responses of every possible pair of measures, for every wave at the same time point. Similar to the test–retest correlations, intercorrelations were calculated separately for females and males of different age groups. Specifically, we computed separate sets of correlations for different age group configurations: 5, 10 and 20-year age bins. We estimated intercorrelations using three different metrics, Pearson's r, Spearman's rho and intraclass correlations (ICC(2,1)), and examined intercorrelations being computed using non-transformed or log-transformed data. As shown in Figure 6, intercorrelations computed using different metrics were highly correlated (r = 0.84 - 0.92), as were the intercorrelations for (non)transformed data (r = 0.95 - 0.96) (Figure 7).

***Additional metrics.*** For each intercorrelation coefficient we additionally recorded the following (variables with an asterisk were included in our main analyses, the rest were included for data quality assessment and data exploration):

- Response information: sample size, maximum age, minimum age, mean age*, median age, standard deviation of age, proportion of female respondents*
- Response properties: the coefficient of variation and skewness of the responses of both measures

**Aggregating intercorrelations.** In an effort to reduce computational costs and the potential occurrence of divergent transitions when conducting the Bayesian meta-analysis, we aggregated the intercorrelations.

We followed a similar approach as for the retest correlations (Figure 16), we first converted each correlation coefficient into Fisher's z, and calculated the corresponding sampling variance. We then split the set of intercorrelations by sample, gender, age group,

and category-domain measure pairs. For each subset we computed a synthesised estimate by aggregating the Fisher's z values using inverse-variance weighting and accounting for the dependency between them as these were computed from the same set or subset of respondents [11]. To average these correlations we used inverse-variance weighting and set the correlation of the sampling errors within subsets to .5. This process resulted in 5'149 aggregated inter-correlations being calculated.

We conducted additional analyses in which we tested the effects of this correlation on our results by setting the correlation to 0.1 and 0.9.

## Analysis

### *Temporal Stability*

**Variance decomposition.** To gain a better understanding of the heterogeneity observed between test–retest correlations, we conducted a variance decomposition analysis by computing the Shapley values for the following predictors:

Panel characteristics

- Panel: Name of the panel

Measure characteristics

- Category: type of measure (i.e., propensity, frequency, behaviour)
- Domain: life domain the measure focuses on (e.g., smoking, driving, social, ethical)
- Scale type: type of response scale (i.e., open-ended/composite index, ordinal/discrete scales)
- Number of items: number of items included in a measure
- Retest Interval: number of years between T1 and T2 data collection

Respondent characteristics

- Age: age group the respondents belong in (10 year bins, e.g., 20-29, 30-39)

- Gender: gender of the respondents (i.e., female, male)
- Number of responses: sample size for each correlation

Shapley values were computed by first estimating a linear regression for each possible combination of predictors (i.e., $2^9$ models for the omnibus analysis, and $2^8$ models for the category-specific analyses) and extracting the adjusted $R^2$ value. Then, for each predictor, we computed the weighted average of the change in adjusted $R^2$ resulting from the inclusion of that predictor in the models.

To obtain bootstrapped confidence intervals, we sampled the dataset of correlations 100 times, and estimated for each predictor a set of 100 Shapley values. To visualise these results, we ranked these values to determine the 50%, 80% and 95% confidence intervals.

**Meta-Analytic Stability and Change model (MASC).**

***Model specification.*** To assess the trajectory of test–retest correlations of risk preference over time we used the MASC model developed by Anusic & Schimmack [10] (Figure 5). Specifically, we were interested in quantifying the effects of gender, linear age, quadratic age, and domain, as well as the interactions between linear and quadratic age with domain on each of the MASC model parameters (i.e, *rel*, *change* and *stabch*). Akin to Anusic and Schimmack [10], we also accounted for the effect of number of items per measure on the *rel* parameter.

In the model, domain was a sum contrast coded factor, gender was the proportion of female respondents (*FemaleProp*) centred at 0.5 (i.e., -0.5 = males and 0.5 = females), item number was a sum contrast coded factor (i.e., -0.5 = "one-item" and 0.5 = "multi-item"), and age (*Age*) corresponded to the mean age of the respondents centred at 40 years and transformed into decades. Quadratic age (*Age2*) was the square value of the *Age* predictor. Lastly, retest interval was coded as the number of decades between waves.

The samples differed from each other on multiple dimensions (e.g, country, mode of data collection), hence, to account for such differences when estimating the MASC model parameters, we included *sample* as a random factor. We limited the (correlated) random

effects structure to the *rel* parameter by adding a varying intercept and varying slopes for the effects of linear age, quadratic age and gender[1]. We did not include a random effects structure for the estimation of the *change* and *stabch* parameters, because to appropriately estimate these parameters samples should have data for a long enough period such that the test–retest correlations assymptote [10]. In the current dataset, the number of test–retest correlations per sample varied substantially, and less than the majority of the samples ($\sim$ 40%) contained retest correlations beyond an interval of 10 years.

The values of *rel*, *change* and *stabch* are bounded between 0 and 1. The *rel* and *change* parameters both represent proportions (i.e., the proportion of reliable between-person variance and the proportion of reliable variance attributable to changing factors, respectively). For the *stabch* parameter (i.e., the rate of change) we need to take into account that over the years changes in individuals' lives accumulate and gradually affect their behaviour to different extents, resulting in decreasing (i.e., $0 <$ rate of change $\leq$ 1) rather than increasing (i.e., rate of change $> 1$) correlations across the years [10]. Therefore, to ensure that these parameters remained within their valid intervals, we modelled them on the logit scale (i.e., *logitrel*, *logitchange* and *logitstabch*), and subsequently back-transformed them via the inverse logit function [12]. Such as to obtain meta-analytic estimates of each parameter, we additionally specified in the model the corresponding standard errors of the (aggregated) retest correlations.

We used Bayesian inference to estimate the meta-analytic model and specified weakly informative priors for the model parameters and hierarchical standard deviations so as to include estimates reported in previous literature (e.g., [5, 10, 13]). The Bayesian hierarchical non-linear model described below was estimated using the probabilistic programming language Stan [14, 15] via the R package *brms* [12, 16, 17]. The companion

[1] We did not include a varying slope for the effect of domain as not every sample had data on each level of domain. Additionally, we did not include a varying slope for item number because most samples solely included single-item measures.

website reports the summary output of the model, sample-specific model predictions, and MCMC diagnostic plots.

$$
\begin{aligned}
y_i &\sim StudentT(\nu, \theta_i, \sqrt{se_i^2 + \sigma^2}) \\
\theta_i &= rel_i \times (change \times (stabch^{\text{time}} - 1) + 1) \\
rel_i &= logit^{-1}(logitrel_i) \\
change &= logit^{-1}(logitchange) \\
stabch &= logit^{-1}(logitstabch) \\
\sigma &\sim Cauchy_{+}(0, 1) \\
\nu &\sim Gamma(2, 0.1)
\end{aligned}
$$

**logitrel$_i$ parameter**

$$
\begin{aligned}
logitrel_i = (\beta_{logitrel_0} + \beta_{logitrel_{0,sample[i]}}) + (\beta_{logitrel_1} + \beta_{logitrel_{1,sample[i]}}))Age + \\
(\beta_{logitrel_2} + \beta_{logitrel_{2,sample[i]}})Age2 + (\beta_{logitrel_3} + \beta_{logitrel_{3,sample[i]}})FemaleProp + \\
\beta_{logitrel_4} Domain + \beta_{logitrel_5}(Age \times Domain) + \beta_{logitrel_6}(Age2 \times Domain) + \beta_{logitrel_7}(Item)
\end{aligned}
$$

$$
\beta_{logitrel_0},\ \beta_{logitrel_1},\ \beta_{logitrel_2},\ \beta_{logitrel_3},\ \beta_{logitrel_4},\ \beta_{logitrel_5},\ \beta_{logitrel_6},\ \beta_{logitrel_7} \sim Normal(0, 1)
$$

$$
\begin{bmatrix} \beta_{logitrel_{0,sample}} \\ \beta_{logitrel_{1,sample}} \\ \beta_{logitrel_{2,sample}} \\ \beta_{logitrel_{3,sample}} \end{bmatrix} \sim MVNormal\left( \begin{bmatrix} 0 \\ 0 \\ 0 \\ 0 \end{bmatrix}, \quad Cov \right)
$$

$$
Cov = \begin{pmatrix}
\sigma^2_{\beta_{logitrel_{0,sample}}} & \sigma_{\beta_{logitrel_{0,sample}}} \sigma_{\beta_{logitrel_{1,sample}}} \rho_{0,1} & \sigma_{\beta_{logitrel_{0,sample}}} \sigma_{\beta_{logitrel_{2,sample}}} \rho_{0,2} & \sigma_{\beta_{logitrel_{0,sample}}} \sigma_{\beta_{logitrel_{2,sample}}} \rho_{0,3} \\
\sigma_{\beta_{logitrel_{1,sample}}} \sigma_{\beta_{logitrel_{0,sample}}} \rho_{0,1} & \sigma^2_{\beta_{logitrel_{1,sample}}} & \sigma_{\beta_{logitrel_{1,sample}}} \sigma_{\beta_{logitrel_{2,sample}}} \rho_{1,2} & \sigma_{\beta_{logitrel_{1,sample}}} \sigma_{\beta_{logitrel_{3,sample}}} \rho_{1,3} \\
\sigma_{\beta_{logitrel_{2,sample}}} \sigma_{\beta_{logitrel_{0,sample}}} \rho_{0,2} & \sigma_{\beta_{logitrel_{2,sample}}} \sigma_{\beta_{logitrel_{1,sample}}} \rho_{1,2} & \sigma^2_{\beta_{logitrel_{2,sample}}} & \sigma_{\beta_{logitrel_{2,sample}}} \sigma_{\beta_{logitrel_{3,sample}}} \rho_{2,3} \\
\sigma_{\beta_{logitrel_{3,sample}}} \sigma_{\beta_{logitrel_{0,sample}}} \rho_{0,3} & \sigma_{\beta_{logitrel_{3,sample}}} \sigma_{\beta_{logitrel_{1,sample}}} \rho_{1,3} & \sigma_{\beta_{logitrel_{3,sample}}} \sigma_{\beta_{logitrel_{2,sample}}} \rho_{2,3} & \sigma^2_{\beta_{logitrel_{3,sample}}}
\end{pmatrix}
$$

$$
\sigma_{\beta_{logitrel_{0,sample}}},\ \sigma_{\beta_{logitrel_{1,sample}}},\ \sigma_{\beta_{logitrel_{2,sample}}},\ \sigma_{\beta_{logitrel_{3,sample}}} \sim Cauchy_{+}(0, 1)
$$

$$\rho \sim LKJCorr(1)$$

**logitchange parameter**

$$logitchange = \beta_{logitchange_0} + +\beta_{logitchange_1} Age + \beta_{logitchange_2} Age2 + \beta_{logitchange_3} FemaleProp + \beta_{logitchange_4} Domain + \beta_{logitchange_5} (Age \times Domain) + \beta_{logitchange_6} (Age2 \times Domain)$$

$$\beta_{logitchange_0}, \beta_{logitchange_1}, \beta_{logitchange_2}, \beta_{logitchange_3}, \beta_{logitchange_4}, \beta_{logitchange_5}, \beta_{logitchange_6} \sim Normal(0, 1)$$

**logitstabch parameter**

$$logitstabch = \beta_{logitstabch_0} + +\beta_{logitstabch_1} Age + \beta_{logitstabch_2} Age2 + \beta_{logitstabch_3} FemaleProp + \beta_{logitstabch_4} Domain + \beta_{logitstabch_5} (Age \times Domain) + \beta_{logitstabch_6} (Age2 \times Domain)$$

$$\beta_{logitstabch_0}, \beta_{logitstabch_1}, \beta_{logitstabch_2}, \beta_{logitstabch_3}, \beta_{logitstabch_4}, \beta_{logitstabch_5}, \beta_{logitstabch_6} \sim Normal(0, 1)$$

**Re-analysis of the Anusic & Schimmack [10] dataset.** We re-analysed the data that the authors made available in the study's supplementary material. The authors collated and analysed test–retest correlations spanning 15 years for assessments of personality traits, self-esteem, life satisfaction, and affect. Prior to any data processing or analysis we excluded from the dataset retest correlations that were computed from samples that had missing sample size information (n = 4), and where respondents were on average below 10 years of age or above 90 years of age (n = 31) leaving a total of 949 test–retest correlations (personality = 226, self-esteem = 196, affect = 101, life satisfaction = 426) for analysis. To remain consistent with how we analysed the other set of retest correlations, prior to estimating the model parameters, we first:

a) calculated the sampling variance of each correlation using the following formula,

$$\frac{\sqrt{(1 - \text{retest}^2)^2}}{n - 1} \tag{A1}$$

b) centered the age variable at 40 years and transformed it into decades,

c) centered the proportion of females variable at 0.5,

d) dichotomized the number of items (i.e., -0.5 = "one-item" and 0.5 = "multi-item"), and

e) rounded the retest interval variable to .25 (i.e., 3 months bins).

Given that in the dataset close to 80% of the studies/samples had 4 or less observations, to avoid poor estimation of varying intercepts and slopes as well as model convergence issues, we did not specify a random effects structure for the *rel* parameter.

By following these data processing and analysis steps we deviated from the original study's analysis in four ways. First, we used a smaller dataset. Second, we carried out the analysis using a Bayesian instead of a Frequentist approach. Third, when conducting the meta-analysis we accounted for the correlations' standard error. Lastly, we changed the moderators that were included in the model by adding an interaction between age linear and construct, between age quadratic and construct, as well as dichotomized the scale length variable for the *rel* parameter.

Details of the model specification in *brms*, model fit and convergence statistics are provided in the companion website.

***Results.*** Here we used a Bayesian approach to directly compare our MASC estimates for different categories of risk preference measures to the estimates obtained by [10] for different psychological constructs (i.e., personality, affect, life satisfaction, and self-esteem). Our reanalysis shows (Figure 14) the highest reliability for personality traits ($M$: 0.66, 95% HDI: [0.61, 0.71]), followed by life satisfaction ($M$: 0.65, 95% HDI: [0.60, 0.69]), self-esteem ($M$: 0.54, 95% HDI: [0.45, 0.64]), and affect ($M$: 0.47, 95% HDI: [0.41, 0.53]). Our results suggest that the average stability of risk preference as captured by propensity and frequency measures, is, on average comparable to that of major psychological constructs. In turn, the reliability of behavioural risk preference measures is lower than any of the four constructs, suggesting a qualitative difference between this

category and the constructs considered. Of course, as suggested above, for frequency measures, some domains show considerably higher/lower levels of stability; consequently, while frequency measures in the smoking and alcohol domains rival the temporal stability of major personality traits, others, like ethical and driving, show some of the lowest reliability estimates observed, suggesting these do not have the same stable quality.

### ***Convergent Validity***

**Variance decomposition.** To gain a better understanding of the heterogeneity in the correlation between different measures, we conducted a variance decomposition analysis. We computed the Shapley values of the following predictors:

<u>Measure characteristics</u>

- Measure category match: whether or not both measures belong to the same category (i.e., propensity, frequency, behaviour)
- Domain match: whether or not both measures focus on the same life domain (e.g., smoking, driving, social, ethical)
- Scale type match: whether or not both measures have the same type of scale (i.e., open-ended/composite index, ordinal/discrete scales)
- Reliability: the average reliability of the measures (using MASC model parameter estimates to make measure and age-specific predictions)

<u>Respondent characteristics</u>

- Age: age group the respondents belong in (10 year bins)
- Gender: gender of the respondents (i.e., female, male)
- Number of responses: sample size for each correlation

**Meta-analyses.** Using the aggregated Fisher's z-transformed correlations, we conducted a Bayesian random-effects meta-analysis to quantify the convergence across all measures, and followed a distributional modelling approach by allowing the samples to vary in their residual standard deviation ($\sigma$).

$$
\begin{aligned}
y_i &\sim StudentT(\nu, \theta_i, \sqrt{se_i^2 + \sigma_i^2}) \\
\theta_i &\sim Normal(\mu_\theta, \tau_\theta) \\
\mu_\theta &\sim Normal(0, 1) \\
\tau_\theta &\sim Cauchy_+(0, 0.3) \\
\log\sigma_i &\sim Normal(\mu_\sigma, \tau_\sigma) \\
\mu_\sigma &\sim Normal(0, 2) \\
\tau_\sigma &\sim Cauchy_+(0, 0.3) \\
\nu &\sim Gamma(2, 0.1)
\end{aligned}
$$

Second, we conducted two meta-regressions with categorical covariates to estimate the convergence between a) different pairs of measure categories (e.g., frequency and propensity),

$$
\begin{aligned}
y_i &\sim StudentT(\nu, \theta_i, \sqrt{se_i^2 + \sigma_i^2}) \\
\theta_i &= \beta_{\theta_{0,sample[i]}} + \beta_{\theta_1} CategoryPair \\
\beta_{\theta_1} &\sim Normal(0, 1) \\
\beta_{\theta_{0,sample}} &\sim Cauchy_+(0, 0.3) \\
\log\sigma_i &= \beta_{\sigma_{0,sample[i]}} + \beta_{\sigma_1} CategoryPair \\
\beta_{\sigma_1} &\sim Normal(0, 2) \\
\beta_{\sigma_{0,sample}} &\sim Cauchy_+(0, 0.3) \\
\nu &\sim Gamma(2, 0.1)
\end{aligned}
$$

and, b) different domains (e.g., propensity-general and frequency-smoking).

$$\begin{aligned}
y_i &\sim StudentT(\nu, \theta_i, \sqrt{se_i^2 + \sigma_i^2}) \\
\theta_i &= \beta_{\theta_{0,sample[i]}} + \beta_{\theta_1} DomainPair \\
\beta_{\theta_1} &\sim Normal(0, 0.5) \\
\beta_{\theta_{0,sample}} &\sim Cauchy_{+}(0, 0.3) \\
\log \sigma_i &= \beta_{\sigma_{0,sample[i]}} + \beta_{\sigma_1} DomainPair \\
\beta_{\sigma_1} &\sim Normal(0, 1) \\
\beta_{\sigma_{0,sample}} &\sim Cauchy_{+}(0, 0.3) \\
\nu &\sim Gamma(2, 0.1)
\end{aligned}$$

In both meta-regressions we specified predictors for the residual standard deviations, and allowed it to vary across the different levels of the categorical variables. Based on recommendations, in all models, we used weakly informative priors [18]. Lastly, we back-transformed the results to Spearman's rho for the reporting.

***Robustness checks.*** We conducted the following additional sets of analyses to check for the robustness of the convergent validity results.

- Re-categorisation of behavioural measures: We treated behavioural measures as belonging to a single overarching "financial" domain (versus 4 domains: occupational, investment, gambling, and insurance). To do so, we first created a new dataset of aggregated intercorrelations to reflect this other categorisation of behavioural measures (resulting in 4'810 correlations), and then repeated the above-described meta-analyses. The results were qualitatively the same as those reported in the main text. We report the these additional analyses in more detail in the companion website.

- Attenuation due to measurement error: Additionally we corrected the 189 meta-analytic estimates of the intercorrelations reported in Figure 7 for attenuation due to measurement error by using the MASC *rel* parameter estimates and Spearman's formula [19]:

$$r_{true} = \frac{r_{obs}}{\sqrt{rel_1 \times rel_2}}$$

As expected, the results (see companion website) show overall increased correlations between measures, and these correlate highly with the uncorrected intercorrelations ($\rho(187) = 0.95$).

## Multiverse Analyses

For brevity and ease of communication, we limited the reporting to a single dataset that was the result of a specific set of data pre-processing and processing choices. To communicate transparently about our results and evaluate their robustness (i.e., how sensitive results were to different data processing choices), we repeated our main analyses using different datasets and model specifications [9]. On the companion website we describe the different steps and choices that were available when constructing and analysing the data, and include a visual summary of the alternative results [20].

**Supplementary Figure 1**

*Overview of the number of items per measure for the different measure categories (propensity, frequency, behaviour).*

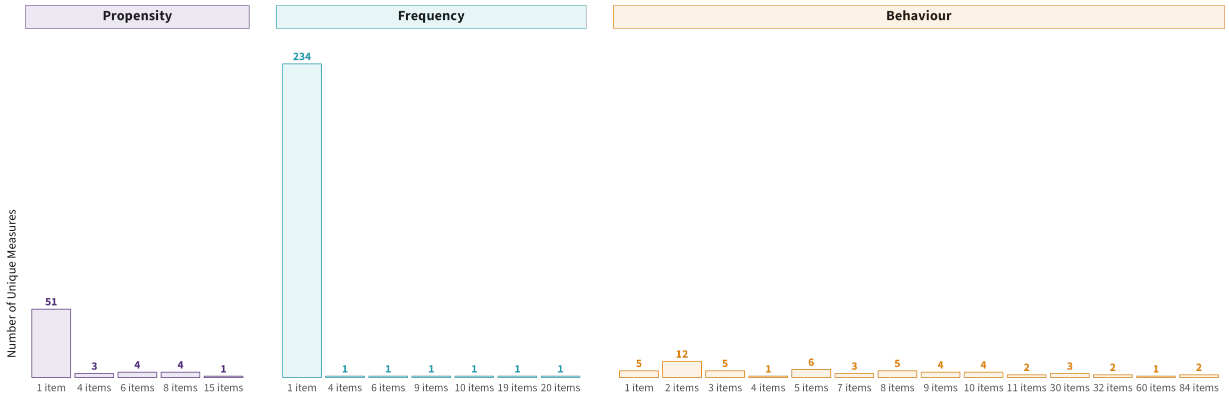

**Supplementary Figure 2**

*Overview of temporal stability measures and correlations. A) The number of measures by category (propensity, frequency, behaviour) and retest interval. B) Distributions of retest correlations as a function of retest interval for the different measure categories (propensity, frequency, behaviour).*

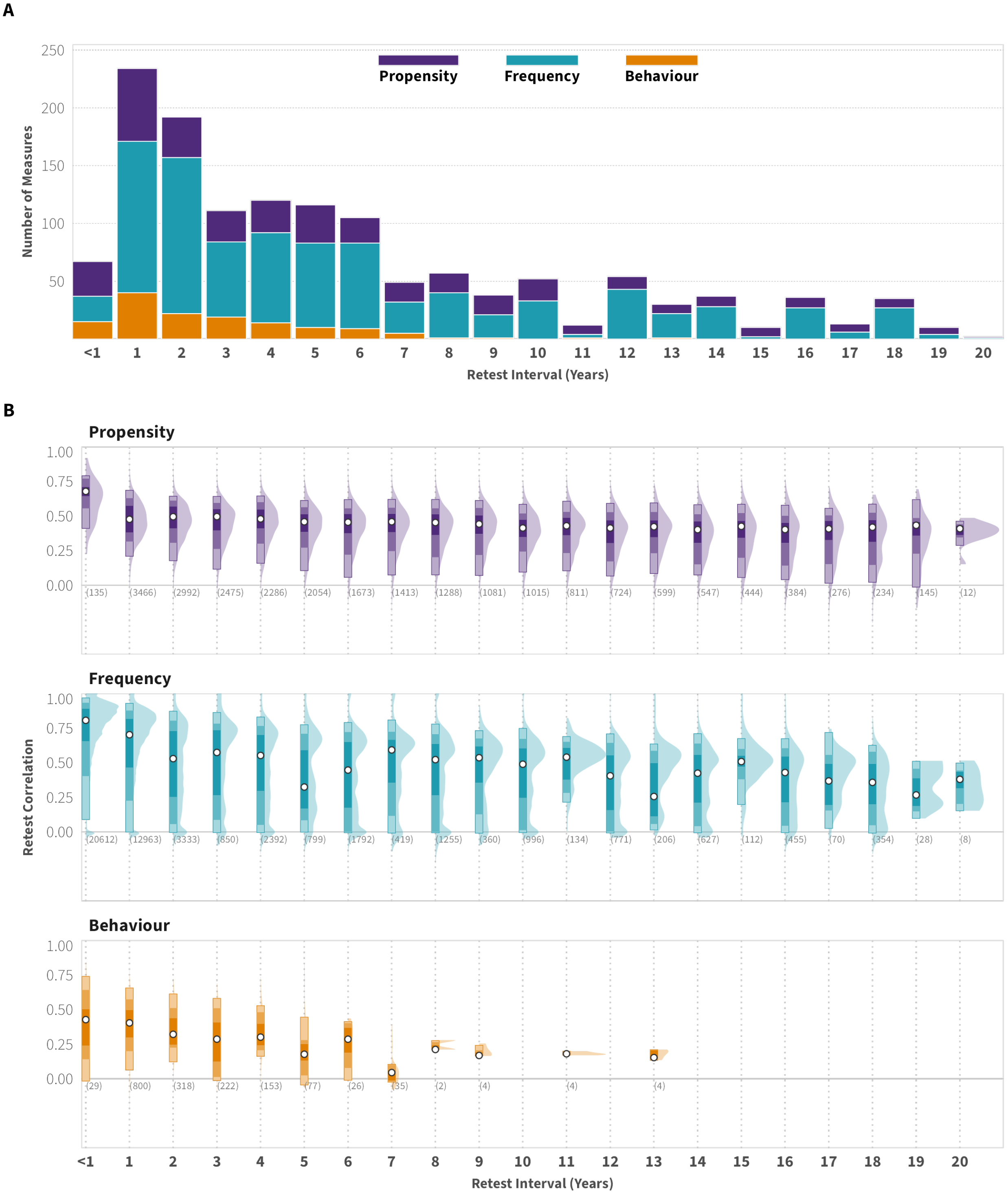

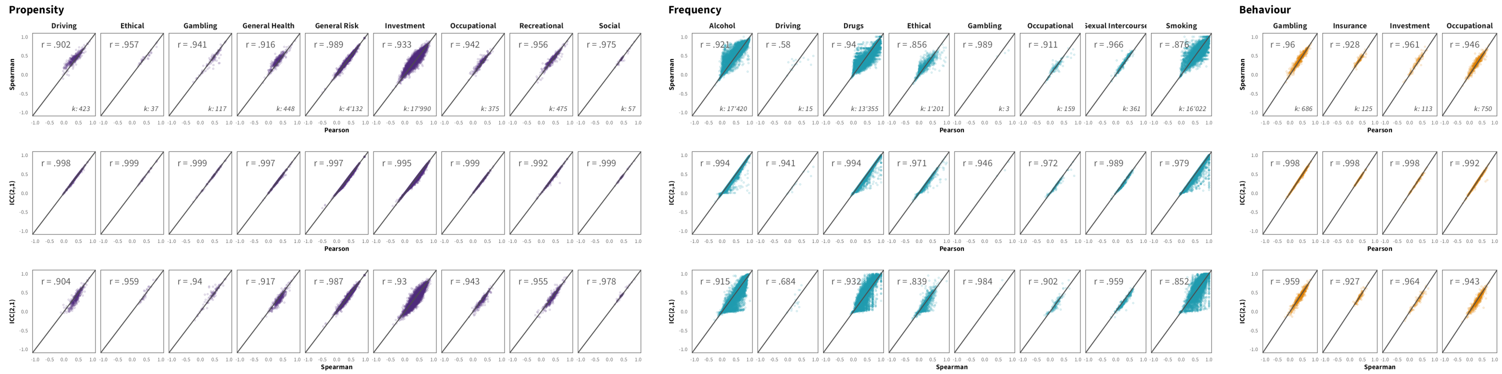


# Supplementary Figure 3

*Scatter plots of test–retest correlations calculated using Pearson's r, Spearman's rho, or ICC(2,1).*

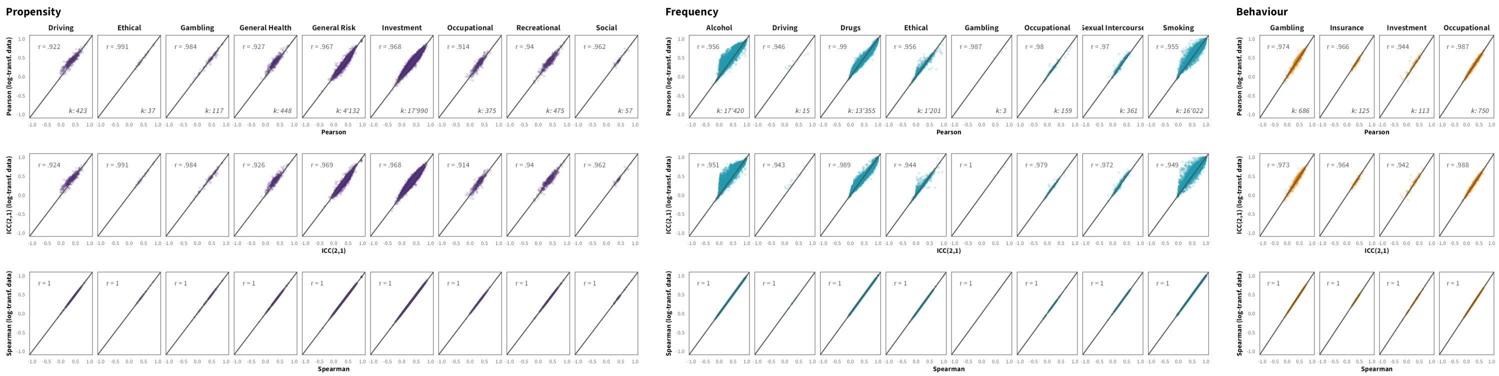


## Supplementary Figure 4

*Scatter plots of different test–retest metrics calculated using either log-transformed or non-transformed data*

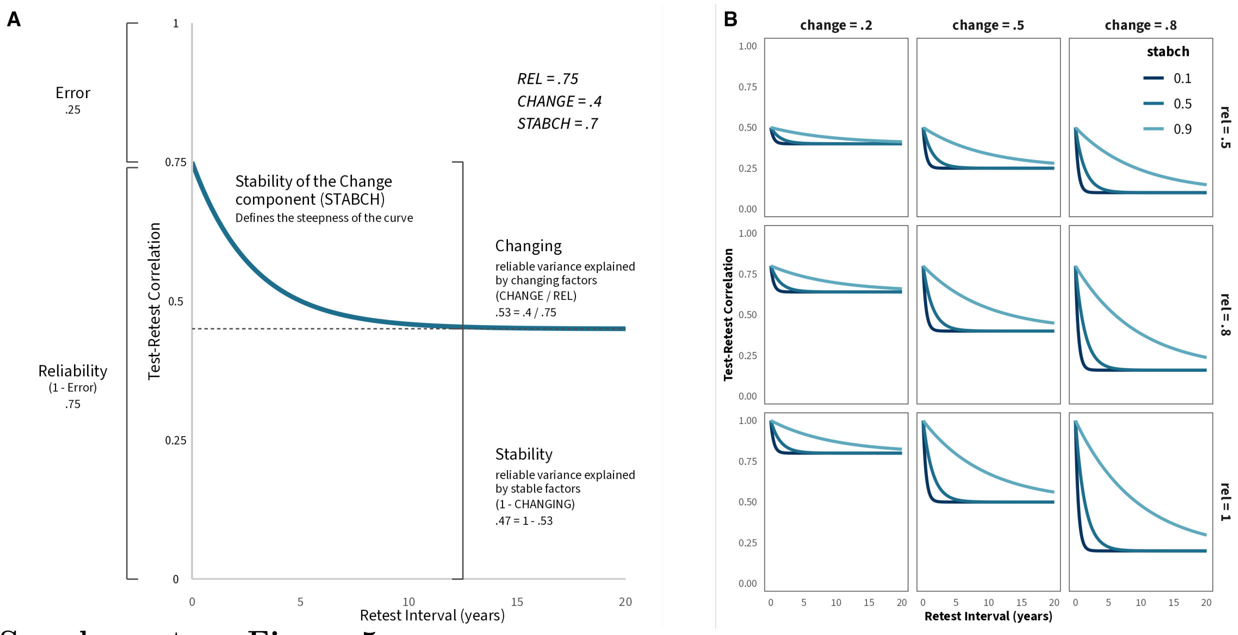


## Supplementary Figure 5

*Depiction of the Meta-Analytic Stability and Change model (MASC). A) Visual depiction of temporal stability curve for major personality traits as estimated by Anusic & Schimmack [10]. B) Examples of different parameterisations of MASC.*

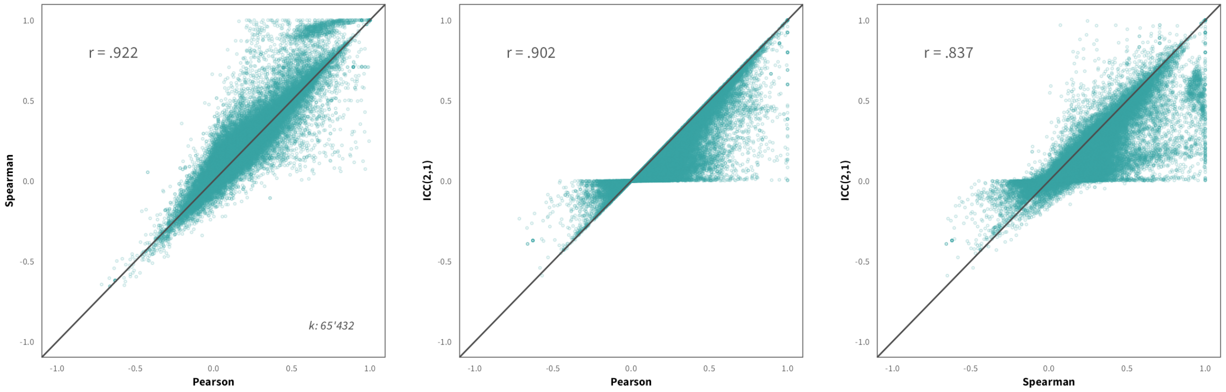


**Supplementary Figure 6**

*Scatter plots of intercorrelations computed using Pearson's r, Spearman's rho, or ICC(2,1).*

1228

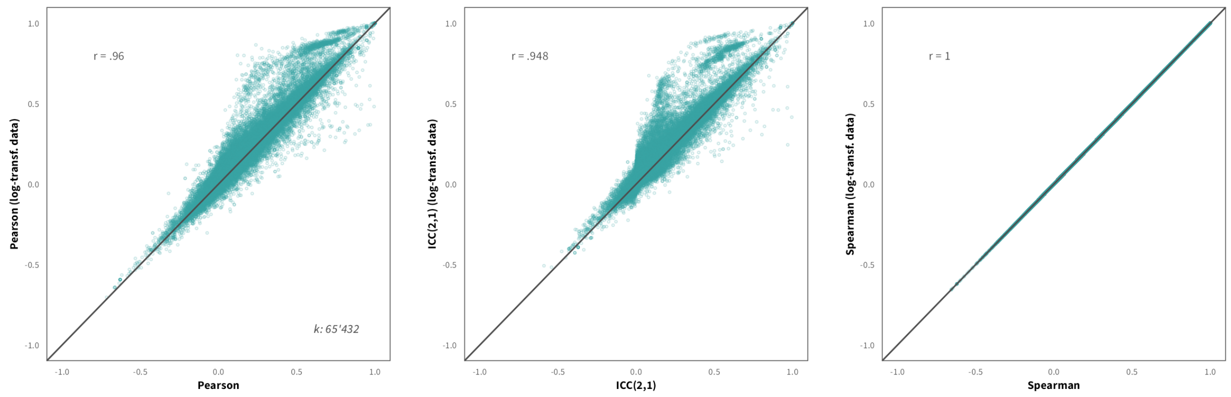


**Supplementary Figure 7**

*Scatter plots of different intercorrelation metrics calculated using either log-transformed or non-transformed data*

**Supplementary Figure 8**

*Expected values of the posterior predictive distribution (mean, 50%, 80%, and 95% HDI) of Meta-Analytic Stability and Change model (MASC) parameters and test–retest correlations for propensity measures of risk preference in the general (k = 1,780), investment (k = 1,083), and driving (k = 196) domains. Left: Predicted values of the Reliability, Change, and Stability of Change parameters, split by domain, age group and gender. Right: Predicted test–retest correlations as a function of time for different age groups (upper panels) and as a function of age for different retest intervals (lower panels).*

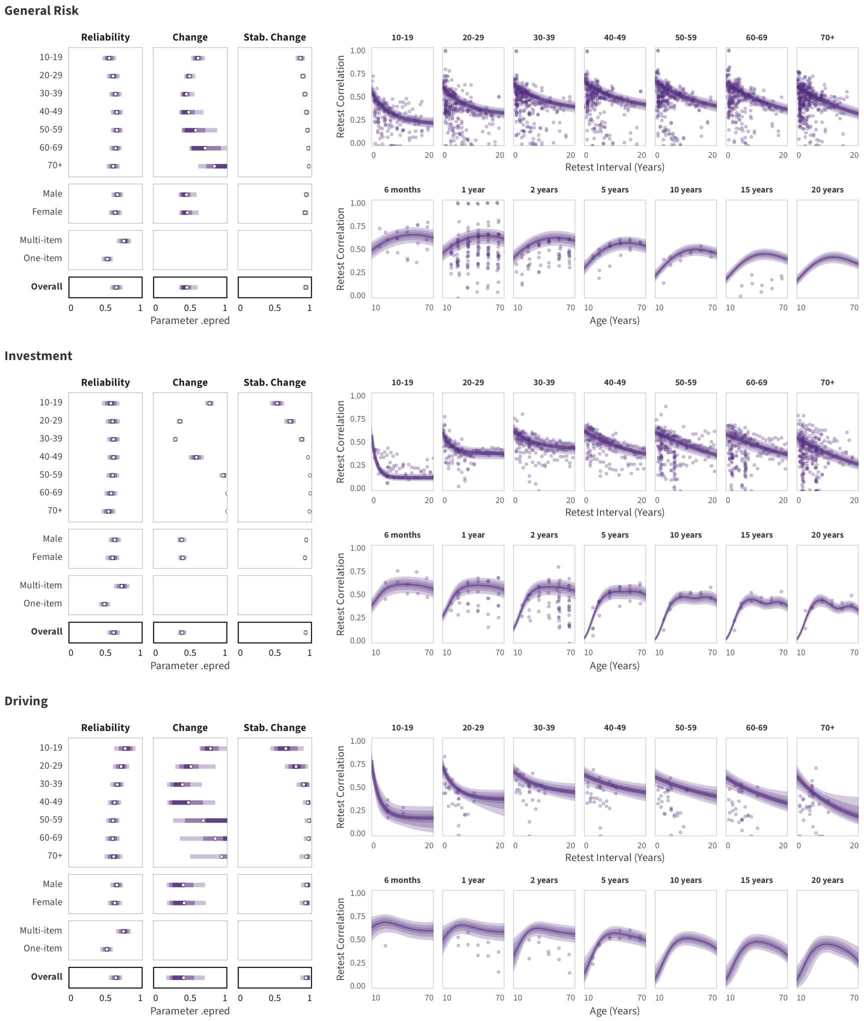

**Supplementary Figure 9**

*Expected values of the posterior predictive distribution (mean, 50%, 80%, and 95% HDI) of Meta-Analytic Stability and Change model (MASC) parameters and test–retest correlations for propensity measures of risk preference in the ethical (k = 21), gambling (k = 66), and general health (k = 212) domains. Left: Predicted values of the Reliability, Change, and Stability of Change parameters, split by domain, age group and gender. Right: Predicted test–retest correlations as a function of time for different age groups (upper panels) and as a function of age for different retest intervals (lower panels).*

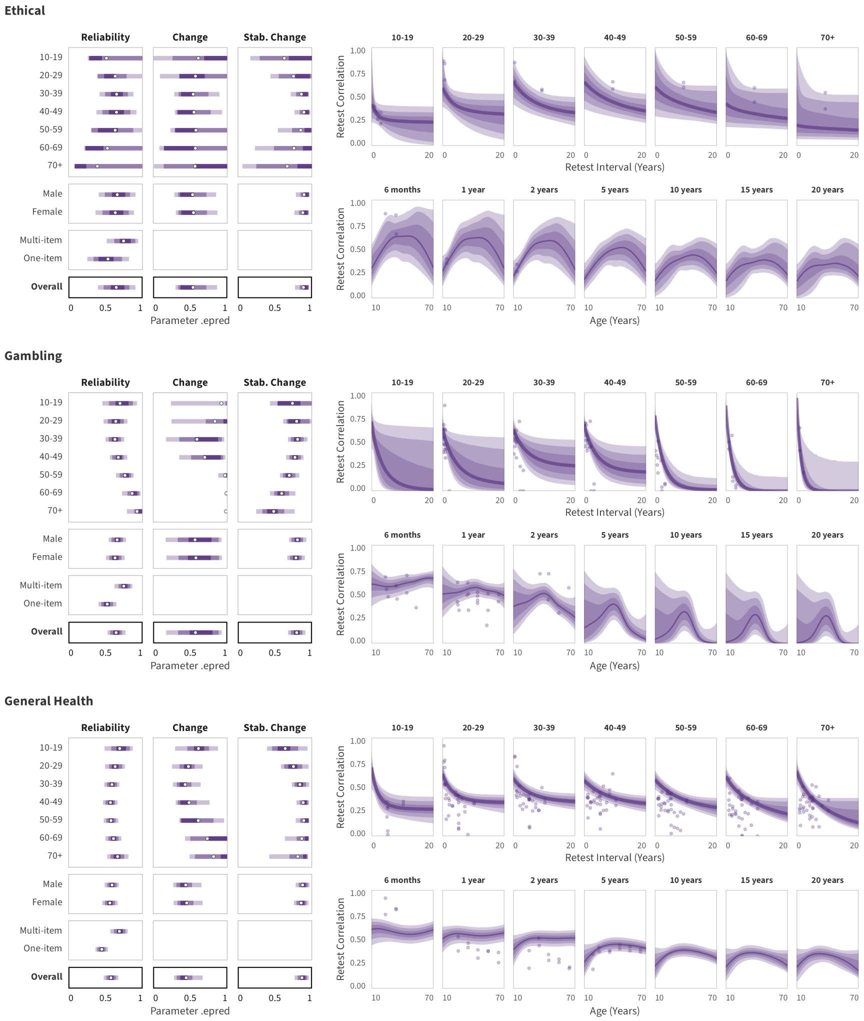

**Supplementary Figure 10**

*Expected values of the posterior predictive distribution (mean, 50%, 80%, and 95% HDI) of Meta-Analytic Stability and Change model (MASC) parameters and test–retest correlations for propensity measures of risk preference in the occupational (k = 181), recreational (k = 201), and social (k = 54) domains. Left: Predicted values of the Reliability, Change, and Stability of Change parameters, split by domain, age group and gender. Right: Predicted test–retest correlations as a function of time for different age groups (upper panels) and as a function of age for different retest intervals (lower panels).*

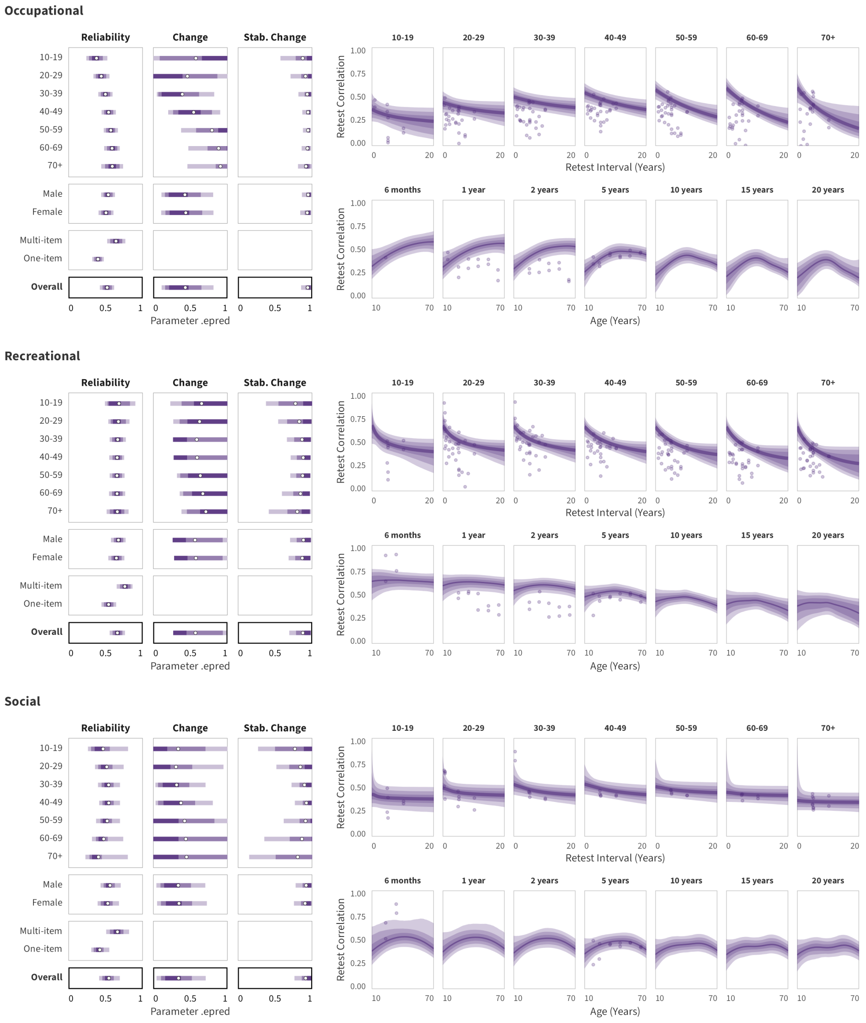

**Supplementary Figure 11**

*Expected values of the posterior predictive distribution (mean, 50%, 80%, and 95% HDI) of Meta-Analytic Stability and Change model (MASC) parameters and test–retest correlations for frequency measures of risk preference in the alcohol (k = 1,733), driving (k = 15), drugs (k = 227), and ethical (k = 92) domains. Left: Predicted values of the Reliability, Change, and Stability of Change parameters, split by domain, age group and gender. Right: Predicted test–retest correlations as a function of time for different age groups (upper panels) and as a function of age for different retest intervals (lower panels).*

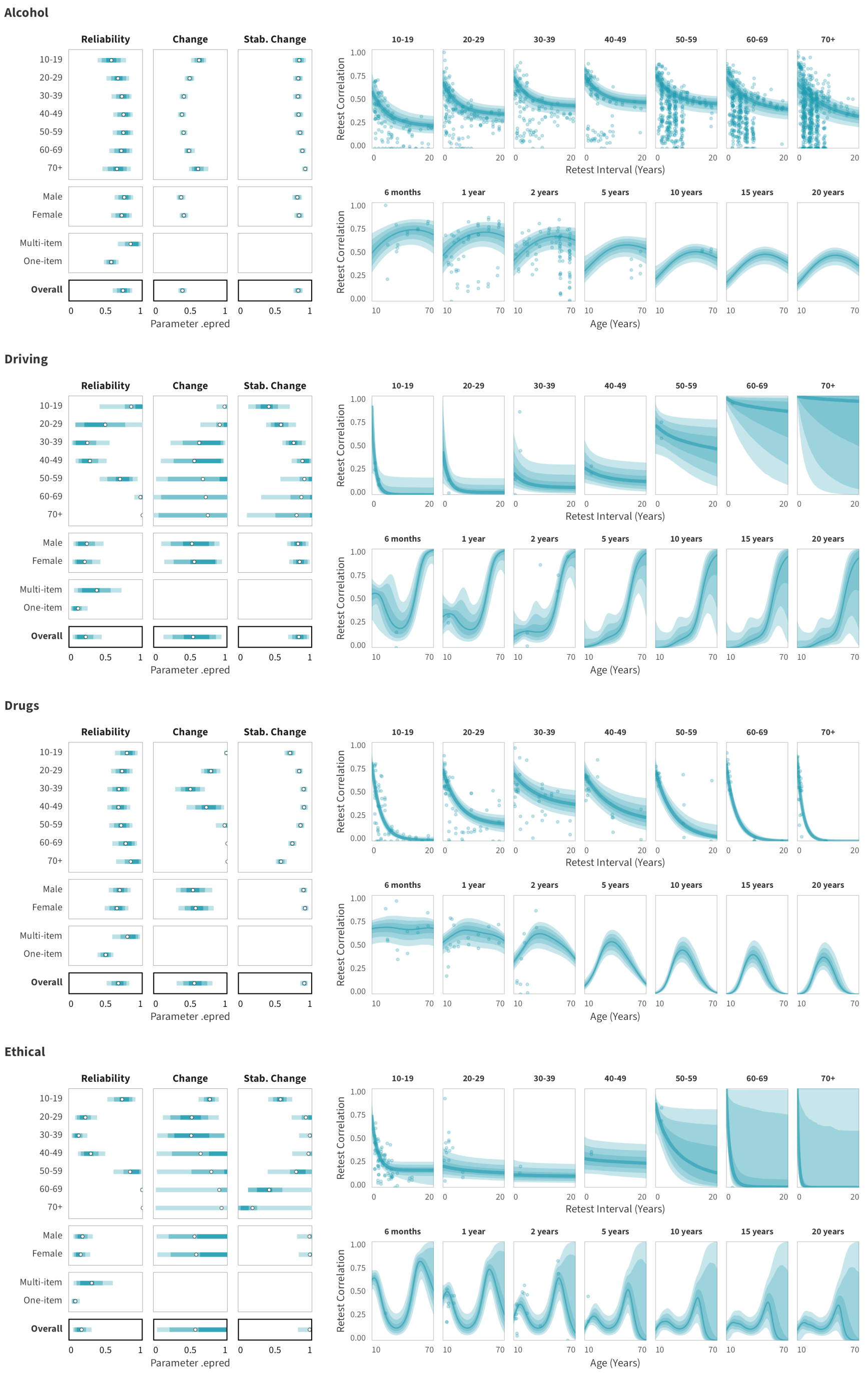

## Supplementary Figure 12

*Expected values of the posterior predictive distribution (mean, 50%, 80%, and 95% HDI) of Meta-Analytic Stability and Change model (MASC) parameters and test–retest correlations for frequency measures of risk preference in the smoking (k = 1,794), sexual intercourse (k = 82), gambling (k = 3), and occupational (k = 17) domains. Left: Predicted values of the Reliability, Change, and Stability of Change parameters, split by domain, age group and gender. Right: Predicted test–retest correlations as a function of time for different age groups (upper panels) and as a function of age for different retest intervals (lower panels).*

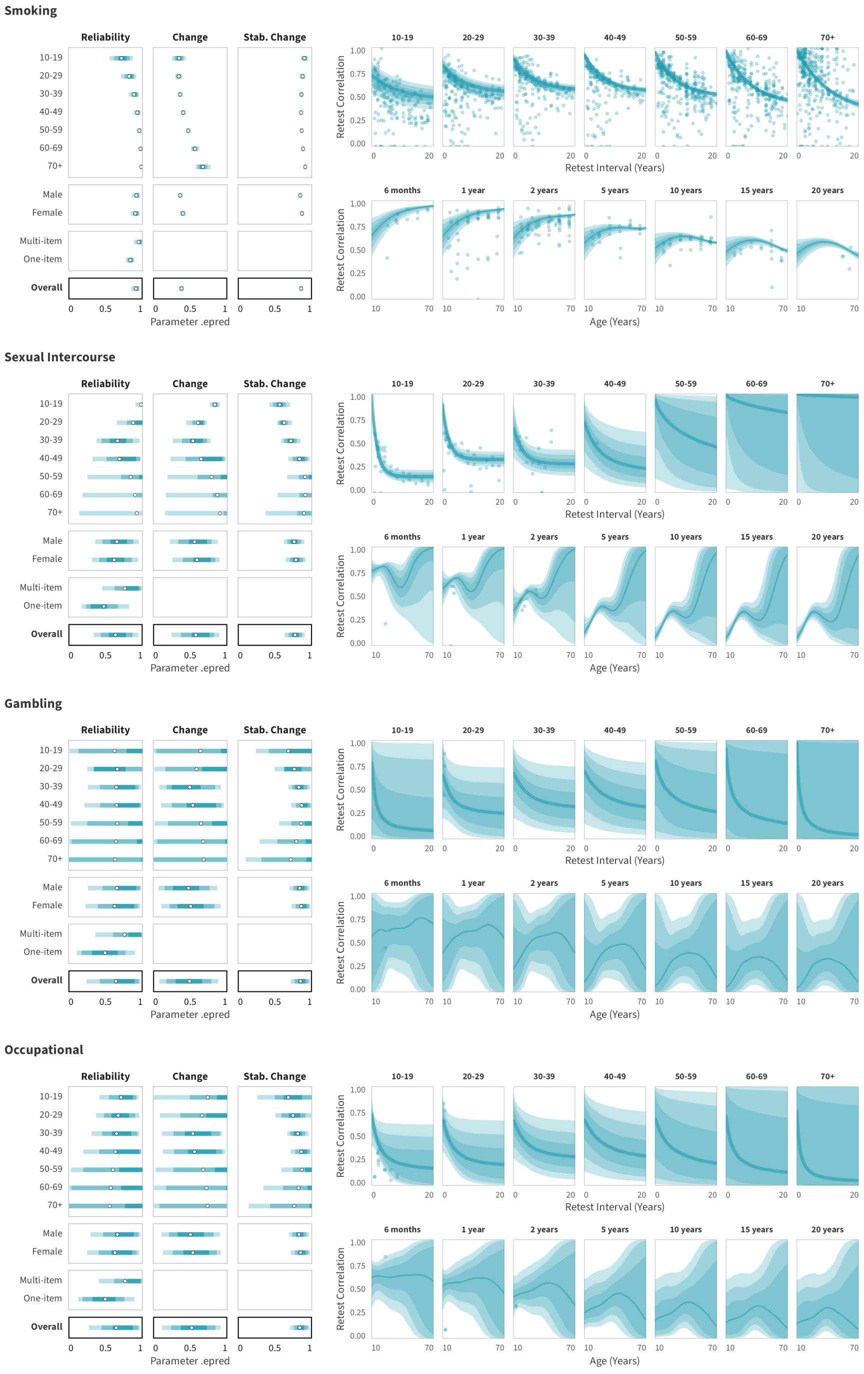

**Supplementary Figure 13**

*Expected values of the posterior predictive distribution (mean, 50%, 80%, and 95% HDI) of Meta-Analytic Stability and Change model (MASC) parameters and test–retest correlations for propensity measures of risk preference in the investment (k = 108), occupational (k = 313), gambling (k = 207), and insurance (k = 80), domains. Left: Predicted values of the Reliability, Change, and Stability of Change parameters, split by domain, age group and gender. Right: Predicted test–retest correlations as a function of time for different age groups (upper panels) and as a function of age for different retest intervals (lower panels).*

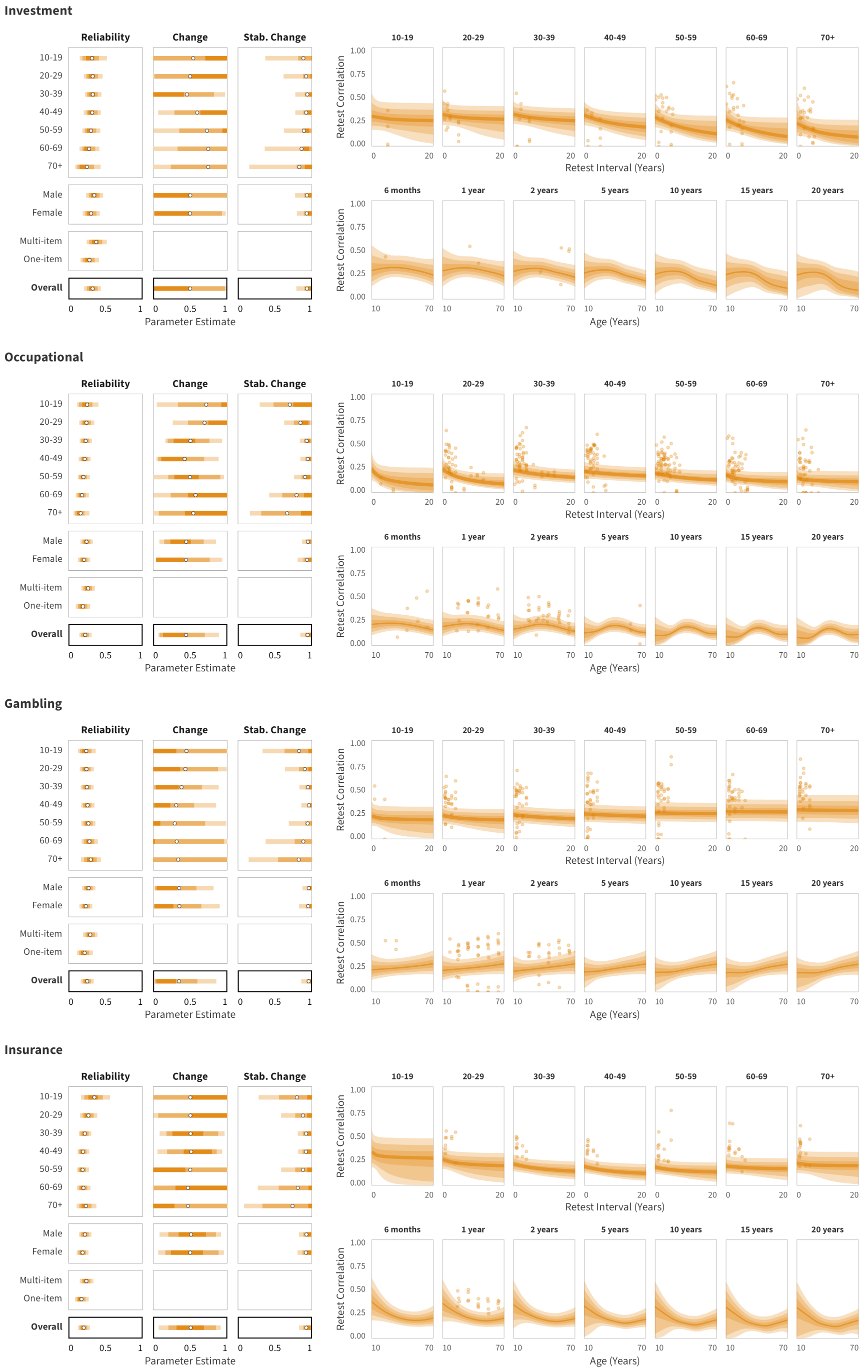

**Supplementary Figure 14**

*Expected values of the posterior predictive distribution (mean, 50%, 80%, and 95% HDI) of Meta-Analytic Stability and Change model (MASC) parameters and test–retest correlations for personality (k = 226), affect (k = 101), life satisfaction (k = 426), and self-esteem (k = 196). Left: Predicted values of the Reliability, Change, and Stability of Change parameters, split by domain, age group and gender. Right: Predicted test–retest correlations as a function of time for different age groups (upper panels) and as a function of age for different retest intervals (lower panels).*

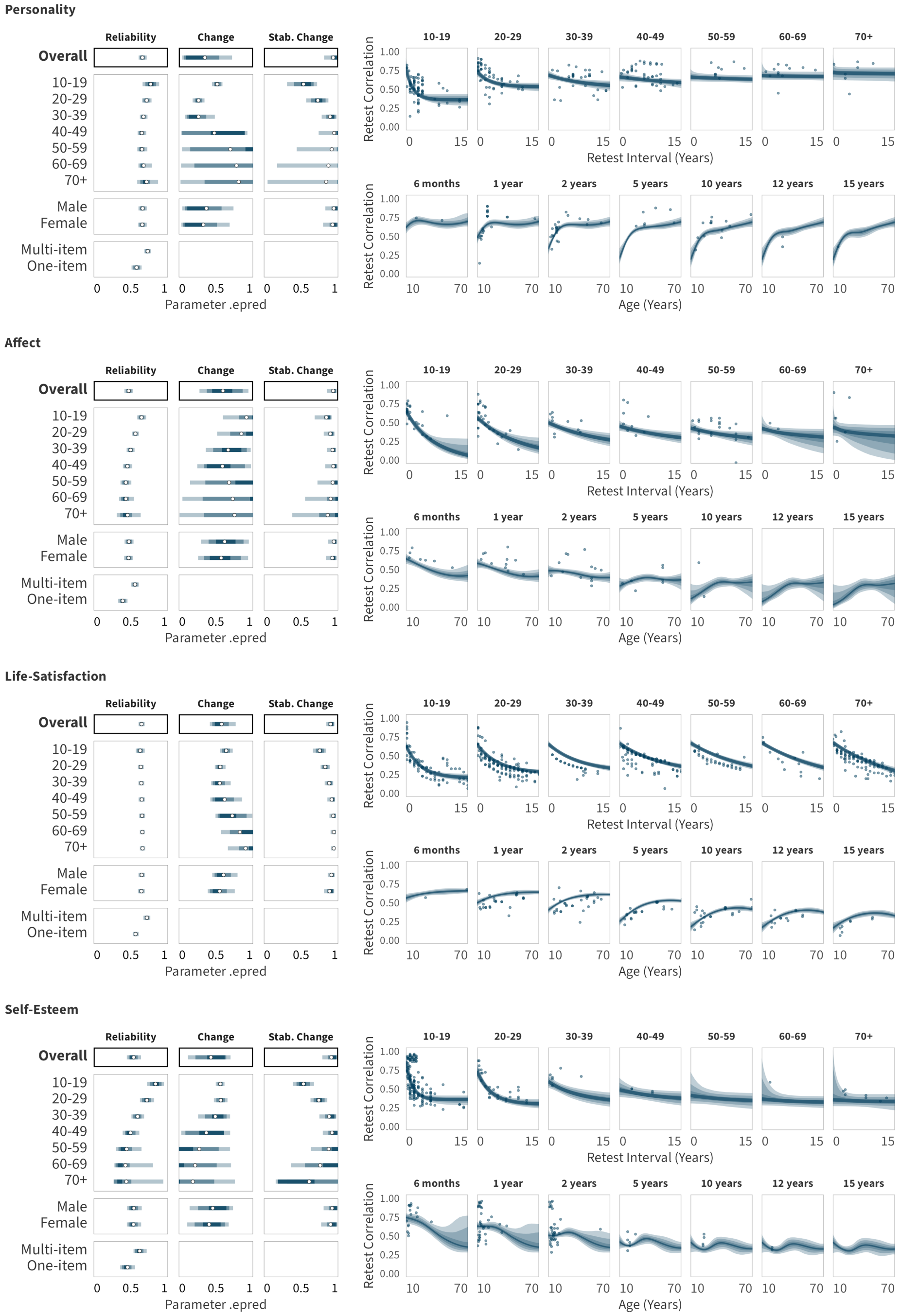

**Supplementary Figure 15**

*Meta-Analytic Stability and Change Model (MASC) parameter estimates. Expected values of the posterior predictive distribution (mean, 50%, 80%, and 95% HDI) of model parameters for a sample of 40-year old individuals for different measure categories of risk preference and other psychological constructs controlling for measure characteristics (multi-item vs single item)*

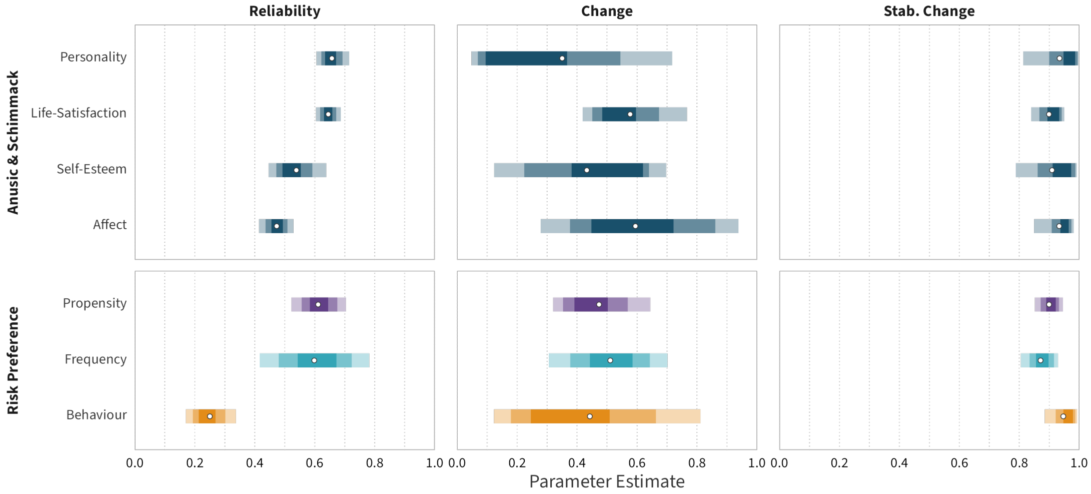

## Supplementary Figure 16

*Overview of the approach used to aggregate test–retest correlations and intercorrelations*

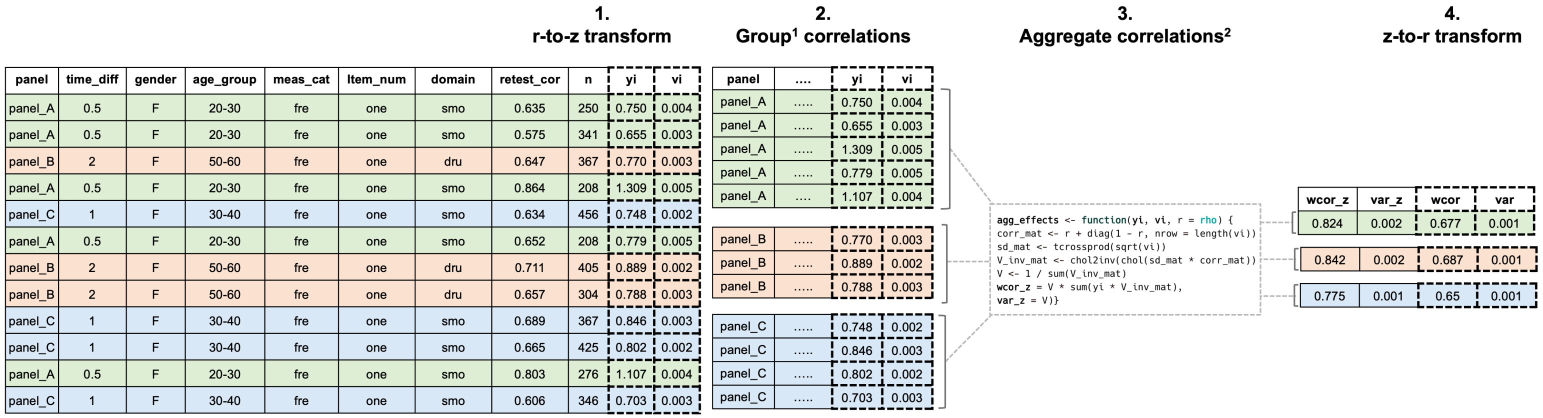


[1] By panel, retest interval, gender, age group, measure category, number of items, and domain

[2] Function from James E. Pustejovsky, J. E. (2019). Sometimes, aggregating effect sizes is fine.

## Supplementary Figure 17

*Convergence of risk preference measures. Distributions of intercorrelations between different risk preference measures at the same measurement occasion (k = 65'432), split by category-domain pairs (A), and category pairs (B).*

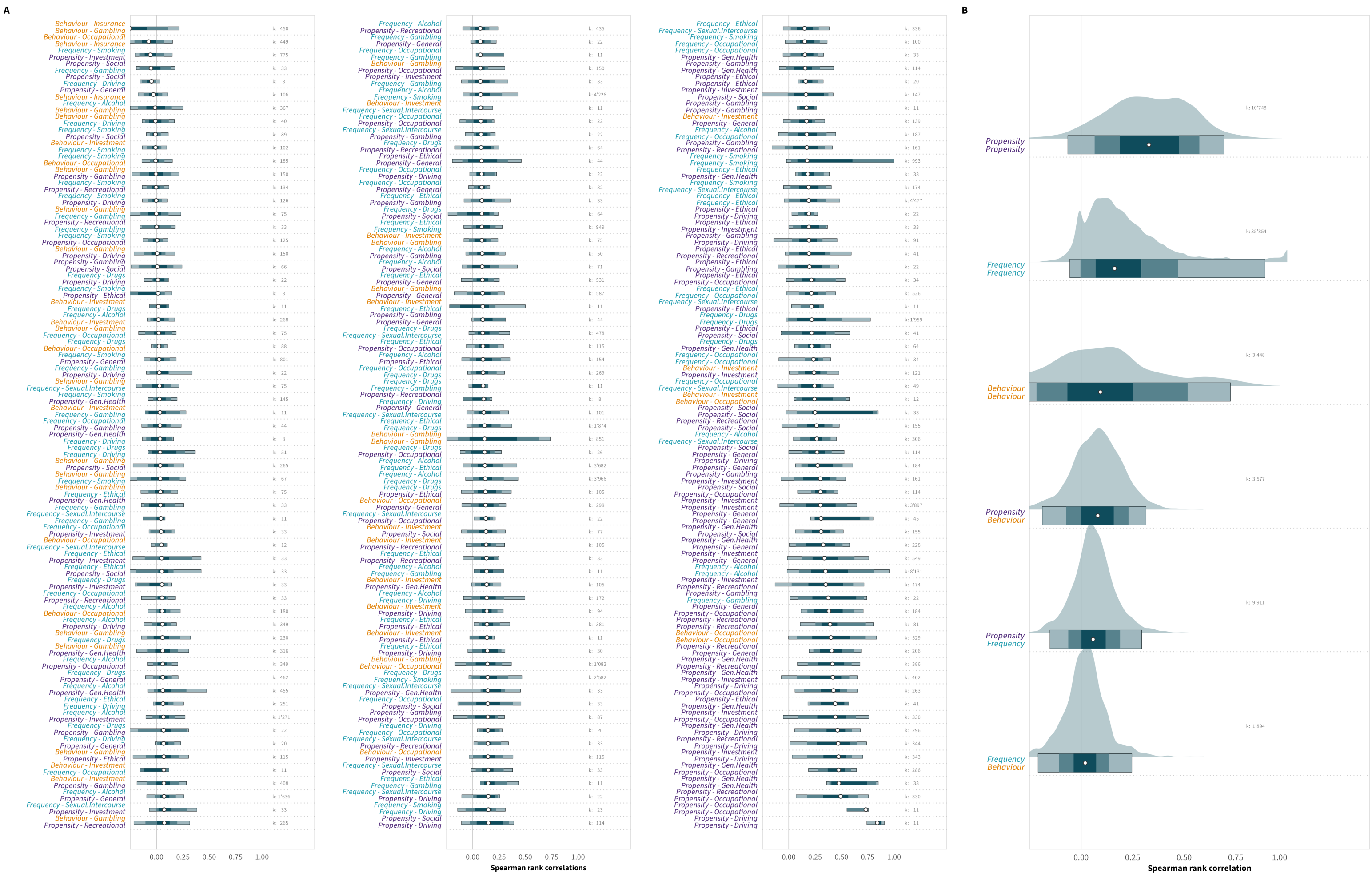

**Supplementary Table 1**

*Search terms used to identify risk preference measures*

| Search terms |
| --- |
| risk ; attitude ; loss/losing/lose ; excit* ; danger* ; avers* ; chance* ; certain ; safe* ; fear* ; |
| adventure/venture ; impuls* ; prefer* ; careful |
| driv* ; car ; fast ; speed ; motor* ; traffic |
| vandal* ; damage ; cheat ; police ; convict* ; arrest* ; gun ; weapon ; shoot/shot ; troubl*; |
| stole/steal ; lie ; crim* ; delinquen*; ; aggressive ; fight* ; |
| assault ; violen* ; injur* ; bully ; affair ; *faith* |
| finan* ; gambl* ; lottery ; coin ; invest* ; stocks ; bet* ; |
| casino ; fund ; poker ; trad* ; shares ; bonds |
| health ; drug ; alcohol ; smok* ; drink* ; cigarette ; drunk ; intox* ; marijuana/cannabis ; heroin ; |
| meth* ; cocaine ; stimulant ; ecstasy ; hallucinogen ; |
| tobacco ; wine ; liquor ; spirit ; beer/pint; unprotected sex/intercourse ; contracept* |
| occupation ; career ; job ; self-employ* ; employ* ; business ; work* ; entrepreneur |
| extreme ; sport ; bar/pub ; night* ; mountain* ; skydiving ; bunjee ; ski ; climb* ; race |
| stranger ; trust |

## Supplementary Table 2

*Overview of panels screened*

| Panel/Sample | Status | Reason for exclusion, if applicable |
|---|---|---|
| Adema, Nikolka, Poutvaara Sunde (2022); Economics Letters (ANPS) - Czeck Republic sample | Incl. | |
| Adema, Nikolka, Poutvaara Sunde (2022); Economics Letters (ANPS) - India sample | Excl. | Small sample size |
| Adema, Nikolka, Poutvaara Sunde (2022); Economics Letters (ANPS) - Mexico sample | Excl. | Small sample size |
| Adema, Nikolka, Poutvaara Sunde (2022); Economics Letters (ANPS) - Spain sample | Incl. | |
| American Life Panel (ALP) | Incl. | |
| American National Election Studies (ANES) | Excl. | Does not include a propensity or behavioural measure |
| Americans' Changing Lives study (ACL) | Excl. | Does not include a propensity or behavioural measure |
| Basel-Berlin Risk Study (BBRS) - Basel (From Frey et al., 2017 Science Advances) | Incl. | |
| Basel-Berlin Risk Study (BBRS) - Berlin (From Frey et al., 2017 Science Advances) | Incl. | |
| Berlin Aging Study (BASE) | Excl. | Restricted data access |
| Berlin Aging Study-II (BASE-II) | Excl. | Restricted data access |
| British Election Study 2005-2009 (BES05) | Incl. | |
| British Election Study 2014-2023 (BES14) | Incl. | |
| Bundesbank - Panel of Household Finances (PHF) | Incl. | |
| Bundesbank Online Panel – Haushalte (BOP-HH)/Survey on Consumer Expectations | Excl. | Propensity measure not asked repeatedly to same respondents. Behavioural measure only Incl. in one wave |
| California Families Project (CFP) | Excl. | Restricted data access |
| Canadian Longitudinal Study on Aging (CLSA) | Excl. | Does not include a propensity or behavioural measure |
| Cape Area Panel Study (CAPS) | Excl. | Propensity item only Incl. in one wave. Does not include a behavioural measure |
| China Health and Retirement Longitudinal Survey (CHARLS) | Excl. | Does not include a propensity or behavioural measure |
| Cognition and Aging in the USA | Excl. | Propensity item only Incl. in one wave. Does not include a behavioural measure |
| Cognitive Economics Project (COGECON) | Incl. | |
| Collaborative Studies on the Genetics of Alcoholism (COGA) | Excl. | Does not include a propensity or behavioural measure |
| Costa Rican Longevity and Healthy Aging Study (CRELES) | Excl. | Does not include a propensity or behavioural measure |
| Crime in the Modern City. A Longitudinal Study of Juvenile Delinquency in Münster (CMC) | Incl. | |
| DNB Household Survey (DHS) | Incl. | |
| Drichoutis Vassilopoulos (2021); Journal of Economics Management Strategy | Incl. | |
| Einstein Aging Study (EAS) | Excl. | Limited documentation + Restricted data access |
| English Longitudinal Study of Ageing (ELSA) | Excl. | Propensity and behavioural measures only Incl. in one wave |
| Enkavi et al., (2019) PNAS | Incl. | |
| Financial Crisis: A Longitudinal Study of Public Response (FICR) | Incl. | |
| Fragile Families and Child Wellbeing Study (FFCWS) | Excl. | Does not include a propensity or behavioural measure |

Table 2 cont.

| Panel/Sample | Status | Reason for exclusion, if applicable |
|---|---|---|
| General Social Survey Panel (GSSP) | Excl. | Propensity item only Incl. in one wave. Does not include a behavioural measure |
| German Internet Panel (GIP) | Incl. | |
| German Longitudinal Election Study (GLES) - Long-term Online Tracking, Cumulation | Excl. | Does not include a propensity or behavioural measure |
| German Longitudinal Election Study (GLES) - Long-term Panel 2002-2005-2009 | Excl. | Does not include a propensity or behavioural measure |
| German Longitudinal Election Study (GLES) - Long-term Panel 2005-2009-2013 | Excl. | Does not include a propensity or behavioural measure |
| German Longitudinal Election Study (GLES) - Long-term Panel 2009-2013-2017 | Excl. | Does not include a propensity or behavioural measure |
| German Longitudinal Election Study (GLES) - Panel 2016-2021 | Incl. | |
| German Longitudinal Election Study (GLES) - Short-term Campaign Panel 2009 | Excl. | Propensity item only Incl. in one wave. Does not include a behavioural measure |
| German Longitudinal Election Study (GLES) - Short-term Campaign Panel 2013 | Excl. | Does not include a propensity or behavioural measure |
| German Longitudinal Election Study (GLES) - Short-term Campaign Panel 2013-2017 (repeatedly questioned respondents) | Incl. | |
| German Longitudinal Election Study (GLES) - Short-term Campaign Panel 2017 | Excl. | Propensity and behavioural measures not asked repeatedly to the same respondents |
| Health and Aging in Africa: A Longitudinal Study of an INDEPTH Community in South Africa (HAALSI) | Excl. | Does not include a propensity or behavioural measure |
| Health Retirement Survey (HRS) | Incl. | |
| Health, Aging, and Retirement in Thailand (HART) | Excl. | Does not include a propensity or behavioural measure |
| Healthy Ageing in Scotland (HAGIS) | Excl. | Only W1/Pilot data available |
| High School and Beyond (HSB) | Excl. | Does not include a propensity or behavioural measure |
| Household Finance and Consumption Survey (HFCS) | Excl. | Household-level question |
| Household, Income and Labour Dynamics in Australia (HILDA) | Incl. | |
| Indonesia Family Life Survey (IFLS) | Incl. | |
| Interdisciplinary Longitudinal Study of Adult Development (ILSE and ILSE.Y) | Excl. | Limited documentation + Restricted data access |
| Japan Household Panel Survey (JHPS) | Excl. | Does not include a propensity or behavioural measure |
| Japanese Study of Aging and Retirement (JSTAR) | Incl. | |
| Korean Labour Income Panel Survey (KLIPS) | Incl. | |
| Korean Longitudinal Study of Aging (KLoSA) | Excl. | Does not include a propensity or behavioural measure |
| Life in Kyrgyzstan Study (LIKS) | Incl. | |
| Longitudinal Aging Study in India (LASI) | Excl. | Only W1/Pilot data available |
| Longitudinal Aging Study of Amsterdam (LASA) | Excl. | Does not include a propensity or behavioural measure |
| Longitudinal Internet studies for the Social Sciences (LISS) | Excl. | Propensity and behavioural measures not asked repeatedly to the same respondents |
| Longitudinal Study of American Youth (LSAY) | Excl. | Does not include a propensity or behavioural measure |
| Longitudinal Study of Australian Children (LSAC) | Excl. | Propensity item only Incl. in one wave. Does not include a behavioural measure |
| Longitudinal Study of Violence Against Women - Men Sample (LSVAW-M) | Incl. | |
| Longitudinal Study of Violence Against Women - Women Sample (LSVAW-W) | Incl. | |

Table 2 cont.

| Panel/Sample | Status | Reason for exclusion, if applicable |
|---|---|---|
| Longitudinal Surveys of Australian Youth (LSAY) | Excl. | Does not include a propensity or behavioural measure |
| Lothian Birth Cohort 1936 | Excl. | Restricted data access |
| Malaysia Ageing and Retirement Survey (MARS) | Excl. | Does not include a propensity or behavioural measure |
| Medical Expenditure Panel Survey (MEPS) | Incl. | |
| Mexican Family Life Survey (MxFLS) | Excl. | Propensity and behavioural measures not asked repeatedly to the same respondents |
| Mexican Health and Aging Study (MHAS) | Excl. | Does not include a propensity or behavioural measure |
| Midlife in Japan (MIDJA) | Incl. | |
| Midlife in the United States (MIDUS) - Milwaukee Dample | Excl. | Restricted data access |
| Midlife in the United States (MIDUS) - Project 1 Sample | Incl. | |
| Millennium Cohort Study (MCS) | Excl. | Propensity and behavioural measures only Incl. in one wave |
| Monitoring the Future: Restricted-Use Panel Data | Excl. | Restricted data access |
| National Health and Nutrition Examination Survey (NHANES) | Excl. | Does not include a propensity or behavioural measure |
| National Income Dynamics Study (NIDS) | Excl. | Does not include a propensity or behavioural measure |
| National Longitudinal Study of Adolescent to Adult Health (Add Health) | Incl. | |
| National Longitudinal Survey of Youth 1979 (NLSY79) | Incl. | |
| National Longitudinal Survey of Youth 1979 Child and Young Adult (NLSY79-CYA) | Incl. | |
| National Longitudinal Survey of Youth 1997 (NLSY97) | Excl. | Propensity and behavioural measures not asked repeatedly to the same respondents |
| National Social Life, Health, and Aging Project (NSHAP) | Incl. | |
| National Survey of Families and Households (NSFH) | Excl. | Does not include a propensity or behavioural measure |
| New Zealand Health, Work and Retirement Study | Excl. | Does not include a propensity or behavioural measure |
| Nießen et al. (2020) . GESIS Instrument | Excl. | Small sample size |
| Northern Ireland Cohort for the Longitudinal Study of Ageing (NICOLA) | Excl. | Propensity item only Incl. in one wave. Does not include a behavioural measure |
| Origin of Variance in the Oldest-Old: Octogenerian Twins (Octo-Twin) | Excl. | Does not include a propensity or behavioural measure |
| Panel Study of Income Dynamics (PSID) | Excl. | Propensity measure not asked repeatedly to same respondents. Behavioural measure only Incl. in one wave |
| Panel Survey of Consumer Finances 1983-1989 | Excl. | Cannot match respondents across waves |
| Panel Survey of Consumer Finances 2007-2009 | Excl. | Household-level question |
| Parenting Across Cultures | Excl. | Restricted data access |
| Preference Parameters Study - China (urban area) (GCOE - CN) | Incl. | |
| Preference Parameters Study - India (rural area) (GCOE - IN Rural) | Incl. | |
| Preference Parameters Study - India (urban area) (GCOE - IN) | Incl. | |
| Preference Parameters Study - Japan (GCOE - JP) | Incl. | |
| Preference Parameters Study - United States of America (GCOE - USA) | Incl. | |

Table 2 cont.

| Panel/Sample | Status | Reason for exclusion, if applicable |
|---|---|---|
| Public Opinion and the Syrian Crisis in Three Democracies | Excl. | Propensity measure only Incl. in one wave. Does not include a behavioural measure |
| Risky decision and happiness task: The Great Brain Experiment smartphone app | Excl. | No comparable retest intervals across respondents |
| Rochester Adult Longitudinal Study (RALS) | Excl. | Does not include a propensity or behavioural measure |
| Rural-Urban Migration in China and Indonesia: China | Excl. | Limited documentation + Restricted data access |
| Rural-Urban Migration in China and Indonesia: Indonesia | Excl. | Limited documentation + Restricted data access |
| Russian Longitudinal Monitoring Survey (RLMS-HSE) | Excl. | Restricted data access |
| Screening Across the Lifespan Twin Study: the Younger (SALTY) | Excl. | Restricted data access |
| Seattle Longitudinal Study (SLS) | Excl. | Does not include a propensity or behavioural measure |
| Socio-Economic Panel Study - Core (SOEP-Core and SOEP-CoV) | Incl. | |
| Socio-Economic Panel Study Retest (SOEP-Retest) | Excl. | Small sample size |
| Sparen und Altersvorsorge in Deutschland (SAVE) | Incl. | |
| Steiner et al., (2020); Decision | Excl. | Small sample size |
| Studies Incl. in Enkavi et al. (2019 PNAS) meta-analyis | Excl. | No studies with open data |
| Studies Incl. in Mata et al. (2018 JEP) meta-analysis | Excl. | No studies with open data |
| Study to Assess Risk and Resilience in Servicemembers — Longitudinal Study (STARRS) | Excl. | Propensity measure not asked repeatedly to same respondents. Does not include a behavioural measure |
| Survey of Consumer Expectations (SCE) | Excl. | Propensity measure not asked repeatedly to same respondents. Does not include a behavioural measure |
| Survey of Health, Ageing and Retirement in Europe (SHARE) (Excluding the following countries: Bulgaria, Croatia, Cyprus, Finland, Greece, Hungary, Latvia, Lithuania, Luxemburg, Malta, Poland, Portugal, Romania, Slovak Republic ) | Excl. | Propensity measure not asked repeatedly to same respondents. Does not include a behavioural measure |
| Survey of Health, Ageing and Retirement in Europe (SHARE) (Including the following countries: Austria, Belgium, Czech_Rep, Denmark, Estonia, France, Germany, Israel, Italy, Netherlands, Slovenia, Spain, Sweden, Switzerland) | Incl. | |
| Swedish Adoption/Twin Study of Aging (SATSA) | Excl. | Small sample size |
| Swiss Household Panel (SHP) | Excl. | Propensity measure not asked repeatedly to same respondents. Does not include a behavioural measure |
| Thailand Vietnam Socio Economic Panel - Thailand (TVSEP-TH) | Excl. | Difficult to match respondents across waves |
| Thailand Vietnam Socio Economic Panel -Vietnam (TVSEP-VN) | Excl. | Difficult to match respondents across waves |
| The Brazilian Longitudinal Study of Aging (ELSI-Brazil) | Excl. | Does not include a propensity or behavioural measure |
| The Irish LongituDinal Study on Ageing (TILDA) | Excl. | Does not include a propensity or behavioural measure |
| Tracking Adolescents' Individual Lives Survey (TRAILS) | Excl. | Propensity and behavioural measures not asked repeatedly to the same respondents |
| TwinLife | Incl. | |
| Twins of Early Development Study (TEDS) | Excl. | Propensity item only Incl. in one wave. Does not include a behavioural measure |
| UK Biobank | Excl. | Does not include a propensity or behavioural measure |

Table 2 cont.

| Panel/Sample | Status | Reason for exclusion, if applicable |
|---|---|---|
| UK Household Longitudinal Survey + British Household Panel Survey (USOC) | Excl. | Propensity measure not asked repeatedly to the same respondents. Does not include a behavioural measure |
| UK Household Longitudinal Survey Innovation Panel (USOC_IP) | Incl. | |
| Ukrainian Longitudinal Monitoring Survey (ULMS) | Incl. | |
| Understanding America Study (UAS) | Incl. | |
| VA Normative Aging Study (VA NAS) | Excl. | Does not include a propensity or behavioural measure |
| WHO Study on global AGEing and adult health (SAGE) | Excl. | Does not include a propensity or behavioural measure |
| Wisconsin Longitudinal Study (WLSG/WLSS) | Excl. | Does not include a propensity measure. Behavioural measure only Incl. in one wave |
| Work and Family Life Study | Excl. | Propensity item only Incl. in one wave. Does not include a behavioural measure |

End of Table

## Supplementary Table 3

*Overview of exclusion and inclusion criteria of measures for the analyses, split by measure category*

| Category | Inclusion | Exclusion | Rationale |
|---|---|---|---|
| All | 1. Measures that have been asked to the same respondents across at least two time points. | 1. Measures that have been asked only in one wave or only once to the respondents | 1. We need responses from a least two time points two compute a test–retest correlation coefficient. |
| All | 2. Measures where the wording and response format remained consistent across at least two time points. | 2. Measures that are not consistent across at least two time points | 2. Measures need to be the same across waves to accurately measure test–retest correlations |
| All | 3. Measures that include at least 4 response options/values, or is composed of multiple (binary) measures that can be aggregated to calculate an index. | 3. Measures that include less than four response options/values (e.g., yes/no, never/sometimes/always). | 3. With more response options it is possible to capture more meaningful changes over time . |
| All | 4. Measures that use an ordinal scale, discrete scale (with a clear response range) or are open-ended | 4. Measures that use a nominal scale or scales with options that cannot be objectively ranked | 4. Can result in subjective interpretations of what a category is and thus reduces response comparability between participants. Further if response options cannot be ranked, this can reduce the accuracy of how the test–retest correlations are computed. |
| Propensity | 1. Measures that ask respondents about recent behaviour. | 1. Measures that ask respondents about behaviour that is too far back in time or no longer relevant (e.g., asking adult respondents about their risk propensity as a child). | 1. Relies on the recollection of certain events, which can result in inaccuracies. We are not capturing temporal stability based on the responses of actions that are no longer relevant . |
| Propensity | 2. Measures that refer directly to the respondent. | 2. Measures that refer to an individual other than the respondent (e.g., partner/spouse, household) | 2. Another person's or group's risk preference is not necessarily reflective of the respondent's. Thus, individual changes would not be reflected in the response. |
| Propensity | 3. Measures that can be answered by both women and men | 3. Gender-specific measures (e.g., specific behaviour during pregnancy) | 3. We want to collect approximately the same amount of responses from both males and females respondents to best explore gender differences. |
| Propensity | 4. Measures that explicitly ask about risk-taking. | 4. Measures that ask about ambiguity. | 4. Ambiguity preference is shown to differ from risk preference [21] |
| Propensity | 5. Measures that can be classified into a general or single life domain (e.g., general, driving, recreational) | 5. Measures for which the behaviour cannot be classified into more than one pre-specified domain | 5. More accurate comparison across domains |
| Frequency | 1. Measures that ask respondents about recent or ongoing behaviour. | 1. Measures that ask respondents about behaviour that is too far in time or no longer relevant (e.g., number of cigarettes smoked before quitting). | 1. Relies on the recollection of certain events, which can result in inaccuracies. Asking about behaviours that are no longer taking place in the present can result in inflated correlation coefficients. |

Table 3 cont.

| Category | Inclusion | Exclusion | Rationale |
| --- | --- | --- | --- |
| Frequency | 2. Measures with a clearly specified time frame (e.g., in the last month/week how often...). | 2. Measures with no clearly specified time frame or that refer to the course of the respondent's life time or that are dependent on a specific event (e.g., since you were 14 years old). | 2. Such questions do not allow a proper comparison between participants as these can result in the subjective interpretation of a time frame or they are dependent on other factors (e.g., current age). |
| Frequency | 3. Measures that refer directly to the respondent. | 3. Measures that refer to an individual other than the respondent (e.g., partner/spouse, household ) | 3. Another person's or group's risk preference is not necessarily reflective of the respondent's. Thus, individual changes would not be reflected in the response. |
| Frequency | 4. Measures that use an ordinal scale, discrete scale (with a clear response range) or are open-ended | 4. Measures that use a nominal scale or scales than cannot be objectively ranked | 4. Can result in subjective interpretations of what a category is and thus reduces response comparability between participants. Further if response options cannot be ranked, this can reduce the accuracy of how the test–retest correlations are computed. |
| Frequency | 5. Measures that include 0 or Never response options | 6. Measures that do not include 0 or Never response options | 5. It is possible to enter a response for those respondent whom this question does not apply (e.g., non-smokers smoking 0 cigarettes). Additionally, such measures help better capture changes across time (e.g., a frequent smoker at T1 but quits smoking at T2) |
| Frequency | 5. Measures that can be answered by both women and men | 6. Gender-specific measures (e.g., specific behaviour during pregnancy) | 6. We want to collect the same amount of responses from both males and females respondents to best explore gender differences. |
| Frequency | 6. Measures that can be classified into a single life domain (e.g., smoking, alcohol, driving) | 6. Measures for which the behaviour can be classified into more than one life domain | 6. More accurate comparison across domains |
| Behaviour | 1. Measures with choices that vary on in terms of probabilities, or that have a clear risk component. | 1. Measures with choices that not solely vary in terms of probabilities (e.g. choices dependent on the response of another individual, choices involving a dimension of time). | 1. Including measures that vary on other dimensions of the choice options would result in risk preference being confounded by other preferences (e..g, social preference, time preference ) |
| Behaviour | 2. Measures with choices that involve a form of monetary outcome or reward. | 2. Measures with choices in non-financial contexts with other forms of outcomes | 2. Such measures allow a direct comparison to tasks commonly using the economics literature |

End of Table

**Supplementary Table 4**

*Overview of panels included in the analyses*

| Sample | Country | Collect | Oper. | Domains | N.meas. | N.waves | N.corr | N |
|---|---|---|---|---|---|---|---|---|
| ADDHEALTH | U.S.A. | Int. | F, P | Alc., Dri., Dru., Eth., Gen., Sex., Smo. | 49 | 5 | 379 | 6,138 |
| ALP | U.S.A. | Onl. | P, B | Gen., Inv., Gam., Occ. | 11 | 18 | 215 | 3,180 |
| ANPS-Czech-Republic | Czech Republic | Onl. | P, B | Gen., Inv. | 2 | 2 | 4 | 230 |
| ANPS-Spain | Spain | Onl. | P, B | Gen., Inv. | 2 | 2 | 5 | 177 |
| BBRS-CH | Switzerland | Lab. | F, B, P | Alc., Inv., Gam., Eth., Occ., Sex., Dru., Hea-gen., Rec., Soc., Dri., Gen. | 35 | 2 | 35 | 34 |
| BBRS-DE | Germany | Lab. | F, B, P | Alc., Inv., Gam., Eth., Occ., Sex., Dru., Hea-gen., Rec., Soc., Dri., Gen. | 35 | 2 | 70 | 99 |
| BES05 | U.K. | Onl. | P | Gen. | 1 | 2 | 12 | 3,291 |
| BES14 | U.K. | Onl. | P, B | Gen., Gam. | 2 | 4 | 64 | 32,982 |
| CMC | Germany | Int. | F, P | Eth., Dru., Occ. | 25 | 4 | 223 | 2,017 |
| COGECON | U.S.A. | Int. | P, B | Inv., Gen. | 3 | 4 | 54 | 871 |
| DHS | Netherlands | Int. | B, P | Gam., Gen., Inv. | 7 | 30 | 14,161 | 10,581 |
| DRICHOUTIS | Greece | Self-adm. | P, B | Gen., Inv. | 2 | 3 | 10 | 113 |
| ENKAVI | U.S.A. | Onl. | F, P, B | Alc., Dri., Dru., Eth., Gam., Hea-gen., Rec., Smo., Soc. | 19 | 2 | 32 | 68 |
| FICR | U.S.A. | Onl. | P | Gam. | 1 | 5 | 70 | 689 |
| GCOE-CN | China | Int. | P | Gen. | 1 | 2 | 10 | 958 |
| GCOE-IN | India | Int. | P, B | Gen., Gam., Occ. | 5 | 5 | 49 | 1,280 |
| GCOE-IN-RUR | India | Int. | B | Gam., Occ. | 4 | 2 | 16 | 263 |
| GCOE-JP | Japan | Self-adm. | P, B | Gen., Occ., Gam., Ins. | 15 | 12 | 949 | 8,040 |
| GCOE-USA | U.S.A. | Self-adm. | P, B | Gen., Occ., Gam., Ins. | 15 | 9 | 684 | 7,523 |
| GIP | Germany | Onl. | P | Gen. | 1 | 3 | 32 | 2,129 |
| GLES-LT | Germany | Int. | P | Gen. | 1 | 6 | 130 | 17,320 |
| GLES-ST | Germany | Onl. | P | Gen. | 1 | 2 | 12 | 2,045 |
| HILDA | Australia | Int. | P, F | Inv., Gen., Smo. | 4 | 21 | 5,976 | 25,154 |
| HRS-Core | U.S.A. | Int. | F, P, B | Alc., Dri., Gen., Hea-gen., Inv., Occ., Rec., Smo. | 15 | 15 | 2,376 | 34,027 |
| IFLS | Indonesia | Int. | F, B | Smo., Occ. | 4 | 5 | 227 | 25,399 |
| JSTAR | Japan | Int. | B | Occ. | 2 | 4 | 54 | 1,905 |
| KLIPS | South Korea | Int. | P, B | Gen., Occ. | 2 | 5 | 64 | 25,962 |
| LIKS | Kyrgyzstan | Int. | F, P | Alc., Gen., Smo. | 8 | 6 | 758 | 10,082 |
| LSVAW-M | U.S.A. | Int. | F, P | Alc., Dru., Eth., Gen., Sex. | 26 | 5 | 306 | 650 |
| LSVAW-W | U.S.A. | Int. | F, P | Alc., Dru., Eth., Gen., Sex. | 23 | 5 | 166 | 1,394 |
| MEPS | U.S.A. | Int. | P | Gen. | 1 | 34 | 272 | 157,599 |
| MIDJA | Japan | Int. | P, F | Gen., Alc. | 6 | 2 | 58 | 655 |
| MIDUS-Project1 | U.S.A. | Int. | F, P | Alc., Dru., Gen., Eth. | 9 | 3 | 181 | 4,357 |
| NLSY79 | U.S.A. | Int. | F, B | Alc., Dru., Smo., Occ. | 41 | 18 | 870 | 12,483 |
| NLSY79-CYA | U.S.A. | Int. | F, P, B | Alc., Dru., Eth., Gen., Occ., Sex., Smo. | 31 | 17 | 4,222 | 8,613 |
| NSHAP | U.S.A. | Int. | F, P | Alc., Gen., Smo. | 5 | 3 | 86 | 2,943 |
| PHF | Germany | Int. | P | Inv., Gen. | 2 | 3 | 56 | 3,566 |
| SAVE | Germany | Self-adm. | F, P | Alc., Dri., Gam., Hea-gen., Inv., Occ., Rec. | 9 | 10 | 1,895 | 3,758 |
| SHARE-Austria | Austria | Int. | F, P | Alc., Inv., Smo. | 7 | 7 | 148 | 4,863 |
| SHARE-Belgium | Belgium | Int. | F, P | Alc., Inv., Smo. | 7 | 7 | 191 | 6,544 |
| SHARE-Czech-Rep | Czech-Rep | Int. | F, P | Alc., Inv., Smo. | 6 | 6 | 159 | 5,673 |
| SHARE-Denmark | Denmark | Int. | F, P | Alc., Inv., Smo. | 8 | 7 | 183 | 4,249 |

Table 4 cont.

| Sample | Country | Collect | Oper. | Domains | N.meas. | N.waves | N. corr | N |
|---|---|---|---|---|---|---|---|---|
| SHARE-Estonia | Estonia | Int. | F, P | Alc., Inv., Smo. | 6 | 4 | 80 | 6,214 |
| SHARE-France | France | Int. | F, P | Alc., Inv., Smo. | 7 | 7 | 183 | 5,593 |
| SHARE-Germany | Germany | Int. | F, P | Alc., Inv., Smo. | 7 | 7 | 160 | 5,463 |
| SHARE-Israel | Israel | Int. | F, P | Alc., Inv., Smo. | 7 | 5 | 68 | 2,665 |
| SHARE-Italy | Italy | Int. | F, P | Alc., Inv., Smo. | 7 | 7 | 185 | 5,251 |
| SHARE-Netherlands | Netherlands | Int. | F, P | Alc., Inv., Smo. | 7 | 5 | 97 | 3,796 |
| SHARE-Slovenia | Slovenia | Int. | F, P | Alc., Inv., Smo. | 6 | 4 | 82 | 3,729 |
| SHARE-Spain | Spain | Int. | F, P | Alc., Inv., Smo. | 7 | 7 | 174 | 6,310 |
| SHARE-Sweden | Sweden | Int. | F, P | Alc., Inv., Smo. | 7 | 7 | 167 | 4,869 |
| SHARE-Switzerland | Switzerland | Int. | F, P | Alc., Inv., Smo. | 7 | 7 | 170 | 3,442 |
| SOEP-Core | Germany | Int. | P, B, F | Dri., Gen., Hea-gen., Inv., Occ., Rec., Smo., Soc. | 11 | 19 | 3,822 | 61,611 |
| TWINLIFE | Germany | Int. | F, P | Alc., Dri., Eth., Gen., Occ. | 18 | 3 | 132 | 9,035 |
| UAS | U.S.A. | Onl. | F, P, B | Alc., Dru., Gen., Inv., Smo. | 13 | 42 | 32,710 | 9,371 |
| ULMS | Ukraine | Int. | F, P, B | Alc., Dri., Gen., Hea-gen., Inv., Occ., Rec., Smo. | 21 | 4 | 277 | 8,154 |
| USOC-IP | U.K. | Int. | F, B, P | Alc., Dru., Eth., Gam., Gen., Hea-gen., Inv., Smo. | 12 | 13 | 493 | 3,707 |

End of Table

Notes. Mode of data collection: Onl(ine), Self-Adm(inistered), Lab(oratory), Int(erview). Measures: P(ropensity), F(requency), and B(ehaviour). Domains: Alc(ohol), Dri(ving), Dru(gs), Eth(ical), Gam(bling), Gen(eral), Hea(lth)-Gen(eral), Ins(urance), Inv(estment), Occ(upational), Rec(reational), Smok(ing), Soc(ial),

## Supplementary Table 5

*Overview and description of the different risk preference measures included in the study, split by measure category, and domain*

| Category | Domain | Description | Example |
|---|---|---|---|
| Propensity | Driving | Respondents indicate on a (ordinal) scale to what extent they are likely to take risks while driving. | *For each of the following statements, please indicate the likelihood that you would engage in the described activity or behaviour if you were to find yourself in that situation: Not wearing a seat belt when being a passenger in the front seat. Very unlikely/Unlikely/Not sure/Likely/Very likely* |
| Propensity | Ethical | Respondents indicate on a (ordinal) scale to what extent they are likely to break rules/laws or cause harm to others or the extent to which they identify/perceive themselves as being someone who breaks rules/laws or causes harm to others. | *For each of the following statements, please indicate the likelihood that you would engage in the described activity or behaviour if you were to find yourself in that situation: Taking some questionable deductions on your income tax return. Very unlikely/Unlikely/Not sure/Likely/Very likely* |
| Propensity | Gambling | Respondents indicate on a (ordinal) scale to what extent they are likely to take risks with gambling-related activities. | *What is the probability that you would do one of the following activities? Please rate on a scale from 0 to 10. Wagering a daily earnings on a bet. 0) very unlikely....10) very likely* |
| Propensity | Health | Respondents indicate on a (ordinal) scale to what extent they are likely to take risks with regards to their health or take part in activities or make decisions that can have detrimental consequences on their health. | *Please indicate the likelihood that you would engage in the described activity or behaviour if you were to find yourself in that situation: Drinking heavily at a social function. Extremely Unlikely (1) - Extremely Likely (7)* |
| Propensity | General | Respondents indicate on a (ordinal) scale to what extent they generally identify as someone who likes to take risks or is willing to take risks. | *Are you generally a person who is willing to take risks or do you try to avoid taking risks? Please answer on a scale from 0 to 10, where 0 means "not at all willing to take risks" and 10 means "very willing to take risks".* |
| Propensity | Investment | Respondents indicate on a (ordinal) scale to what extent they are likely to take risks with investments. | *Which of the statements comes closest to the amount of financial risk that you are willing to take when you save or make investments? Take substantial financial risks expecting to earn substantial returns / Take above average financial risks expecting to earn above average returns / Take average financial risks expecting to earn average returns / Not willing to take any financial risks* |
| Propensity | Occupation | Respondents indicate on a (ordinal) scale to what extent they are likely to take risks with regards to their job. | *Rate using a scale from 0 to 10. I don't mind taking risks in ... my professional career* |
| Propensity | Recreation | Respondents indicate on a (ordinal) scale to what extent they are likely to take risks with regards recreational actives or their likelihood of engaging in activities that involve height and/or speed and high risk of serious injury or death. | *For each of the following statements, please indicate your likelihood of engaging in each activity or behaviour: Going down a ski run that is beyond your ability or closed. Very unlikely/Unlikely/Not sure/Likely/Very likely* |
| Propensity | Social | Respondents indicate on a (ordinal) scale to what extent they are likely to take risks in social situations, or when trusting strangers. | *For each of the following statements, please indicate your likelihood of engaging in each activity or behaviour: Admitting that your tastes are different from those of your friends. Very unlikely/Unlikely/Not sure/Likely/Very likely.* |

Table 5 cont.

| Measure | Domain | Description | Example |
|---|---|---|---|
| Frequency | Alcohol | Respondents quantify the extent to which they consumed alcohol or experienced the consequences of alcohol consumption within a certain time frame. | *How many times in the last four weeks have you had an alcoholic drink? Most days / Once or twice a week / 2 or 3 times / Once only / Never* |
| Frequency | Driving | Respondents quantify the extent to which they have not been prudent while driving a vehicle within a certain time frame. | *During the past 30 days, how often did you drive a car or other vehicle when you had been drinking alcohol?* |
| Frequency | Drug | Respondents quantify the extent to which they consumed drugs or experienced the consequences of drug consumption within a certain time frame. | *During the last 30 days, how often, if ever, did you use these other drugs? Heroin, steroids, or MDMA (Ecstasy). 0) Never, 1) Less than once a week, 2) 1 or 2 days per week, 3) 3 or 4 days per week, 4) 5 or 6 days per week, 5) Every day* |
| Frequency | Ethical | Respondents quantify the extent to which they broke rules/laws or had issues with the law or cause harm to others within a certain time period. | *This is about fare dodging. How often did you do that in the last 12 months? Indicate number of times.* |
| Frequency | Gambling | Respondents quantify the extent to which they partook in gambling-related activities within a certain time frame. | Pathological gambling [22] |
| Frequency | Occupation | Respondents indicate the extent to which they have been reckless at their job/school or behaved in a way that could lead to them losing their job/get in trouble at school. | *This is about skipping school. How often did you do that in the last 12 months?* |
| Frequency | Sexual Intercourse | Respondents indicate the number of sexual partners or how often they had sexual intercourse without using a form of contraception within a certain time frame. | *With how many persons are you currently having a romantic or sexual relationship?* |
| Frequency | Smoking | Respondents quantify the extent to which they smoke cigarettes or other tobacco products within a certain time frame. | *About how many cigarettes or packs do you usually smoke in a day now?* |
| Behaviour | Gambling | These tasks mention a gambling-related activity/scenario or a form of game. Respondents are asked to decide between two or more options that offer different potential monetary gains and/or losses with varying probability. Also includes Willingness to Pay and Willingness to Accept tasks. Depending on the respondents' responses in such tasks, composite measures can be derived which summarise their tolerance towards risk (e.g., proportion of safe choices, risk aversion category). Such tasks can involve decision from experience or description, with hypothetical or incentivised choices. | *Now, imagine you have a choice between the following two options: Option A: A lottery with a 50% chance of winning 80$ and a 50% chance of losing 50$ / Option B: Zero dollars. Which option would you choose?* |
| Behaviour | Insurance | Tasks require respondents make choices about insurances, with hypothetical or incentivised choices. Also includes Willingness to Pay and Willingness to Accept tasks. Depending on the respondents' responses in such tasks, composite measures can be derived which summarise their tolerance towards risk (e.g., proportion of safe choices, risk aversion category). | *Assume that you know there is a 50% chance of losing $1000 on a given day. You can take out insurance to cover this amount in case of loss. If an insurance policy is sold as listed below, would you purchase it?* |

Table 5 cont.

| Measure | Domain | Description | Example |
| --- | --- | --- | --- |
| Behaviour | Investment | These tasks explicitly mention an investment-related activity/scenario. Respondents can be asked how much of an endowment they wish to allocate to different options. These tasks can be hypothetical or incentivised. | *Imagine that you had won 100,000 euros in the lottery. Immediately after receiving your winnings you receive the following offer: You have the chance to double your money. But it is equally possible that you will lose half of the amount invested. You can participate by staking all or part of your 100,000 euros on the lottery, or choose not to participate at all. What portion of your lottery winnings would you be prepared to stake on this financially risky yet potentially lucrative lottery investment?* |
| Behaviour | Occupational | Tasks require respondents to make choices about jobs offering different salaries with different probabilities. Depending on the respondents' responses in such tasks, composite measures can be derived which summarise their tolerance towards risk (e.g., proportion of safe choices, risk aversion category). | *Which ONE do you prefer? Option A: A 50% chance of the salary increasing by 30%, but also a 50% chance of the salary increasing by 11%: Option B: Guaranteed salary increase of 20%.* |

End of Table

## References

1. Arslan, R. C. *et al.* How People Know Their Risk Preference. *Scientific Reports* **10,** 15365. ISSN: 2045-2322. (2022) (Sept. 2020).

2. Eisenberg, I. W. *et al.* Uncovering the Structure of Self-Regulation through Data-Driven Ontology Discovery. *Nature Communications* **10,** 2319. ISSN: 2041-1723. (2022) (May 2019).

3. Enkavi, A. Z. *et al.* Large-Scale Analysis of Test–Retest Reliabilities of Self-Regulation Measures. *Proceedings of the National Academy of Sciences* **116,** 5472–5477. (2022) (Mar. 2019).

4. Falk, A. *et al.* Global Evidence on Economic Preferences. *The Quarterly Journal of Economics* **133,** 1645–1692. ISSN: 0033-5533. (2021) (Nov. 2018).

5. Frey, R., Pedroni, A., Mata, R., Rieskamp, J. & Hertwig, R. Risk Preference Shares the Psychometric Structure of Major Psychological Traits. *Science Advances* **3,** e1701381. (2021) (2017).

6. Chapman, J., Dean, M., Ortoleva, P., Snowberg, E. & Camerer, C. *Econographics* tech. rep. w24931 (National Bureau of Economic Research, Cambridge, MA, Aug. 2018), w24931. (2022).

7. Harrison, G. W. in *Handbook of Choice Modelling* (eds Hess, S. & Daly, A.) 236–254 (Edward Elgar Publishing, Aug. 2014). ISBN: 978-1-78100-315-2. (2023).

8. Schönbrodt, F. D. & Perugini, M. At What Sample Size Do Correlations Stabilize? *Journal of Research in Personality* **47,** 609–612. ISSN: 1095-7251 (2013).

9. Steegen, S., Tuerlinckx, F., Gelman, A. & Vanpaemel, W. Increasing Transparency through a Multiverse Analysis. *Perspectives on Psychological Science* **11,** 702–712. ISSN: 1745-6916. (2022) (Sept. 2016).

10. Anusic, I. & Schimmack, U. Stability and Change of Personality Traits, Self-Esteem, and Well-Being: Introducing the Meta-Analytic Stability and Change Model of Retest Correlations. *Journal of Personality and Social Psychology* **110,** 766–781. ISSN: 1939-1315 (2016).

11. Hedges, L. V. & Olkin, I. *Statistical Methods for Meta-Analysis* 1st Ed. ISBN: 978-0-08-057065-5 (Academic Press, London, 1985).

12. Bürkner, P.-C. Bayesian Item Response Modeling in R with Brms and Stan. *Journal of Statistical Software* **100,** 1–54. ISSN: 1548-7660. (2022) (Nov. 2021).

13. Mata, R., Frey, R., Richter, D., Schupp, J. & Hertwig, R. Risk Preference: A View from Psychology. *Journal of Economic Perspectives* **32,** 155–172. ISSN: 0895-3309. (2021) (May 2018).

14. Stan Development Team. *Stan User's Guide. Version 2.29* 2022.

15. Carpenter, B. *et al.* Stan: A Probabilistic Programming Language. *Journal of Statistical Software* **76,** 1–32. ISSN: 1548-7660. (2022) (Jan. 2017).

16. Bürkner, P.-C. Advanced Bayesian Multilevel Modeling with the R Package Brms. *The R Journal* **10,** 395–411. ISSN: 2073-4859. (2022) (2018).

17. Bürkner, P.-C. Brms: An R Package for Bayesian Multilevel Models Using Stan. *Journal of Statistical Software* **80,** 1–28. ISSN: 1548-7660. (2022) (Aug. 2017).

18. Williams, D. R., Rast, P. & Bürkner, P.-C. *Bayesian Meta-Analysis with Weakly Informative Prior Distributions* Jan. 2018. (2023).

19. Spearman, C. The Proof and Measurement of Association between Two Things. *The American Journal of Psychology* **15,** 72. ISSN: 00029556. JSTOR: 1412159. (2024) (Jan. 1904).

20. Hall, B. D. *et al.* A Survey of Tasks and Visualizations in Multiverse Analysis Reports. *Computer Graphics Forum* **41,** 402–426. ISSN: 1467-8659. (2023) (2022).

21. Levy, I., Snell, J., Nelson, A. J., Rustichini, A. & Glimcher, P. W. Neural Representation of Subjective Value Under Risk and Ambiguity. *Journal of Neurophysiology* **103,** 1036–1047. ISSN: 0022-3077. (2023) (Feb. 2010).

22. Brodbeck, J., Duerrenberger, S. & Znoj, H. Prevalence Rates of at Risk, Problematic and Pathological Gambling in Switzerland. *The European Journal of Psychiatry* **23,** 67–75. ISSN: 0213-6163. (2023) (June 2009).
