# Supplementary material for: A systematic review and meta-analyses of the temporal stability and convergent validity of risk preference measures
Source: Nat Hum Behav. 2025 Jan 27;9(4):700–12. doi: 10.1038/s41562-024-02085-2 (PMC12018263; doi:10.1038/s41562-024-02085-2)
Supplement: Supplementary file 2 — Reporting Summary [file 41562_2024_2085_MOESM2_ESM.pdf]

nature portfolio

Corresponding author(s): Rui Mata

Last updated by author(s): May 23, 2024

Reporting Summary

Nature Portfolio wishes to improve the reproducibility of the work that we publish. This form provides structure for consistency and transparency in reporting. For further information on Nature Portfolio policies, see our Editorial Policies and the Editorial Policy Checklist.

Statistics

For all statistical analyses, confirm that the following items are present in the figure legend, table legend, main text, or Methods section.

n/a	Confirmed	
☐	☒	The exact sample size (*n*) for each experimental group/condition, given as a discrete number and unit of measurement
☐	☒	A statement on whether measurements were taken from distinct samples or whether the same sample was measured repeatedly
☒	☐	The statistical test(s) used AND whether they are one- or two-sided<br>*Only common tests should be described solely by name; describe more complex techniques in the Methods section.*
☐	☒	A description of all covariates tested
☒	☐	A description of any assumptions or corrections, such as tests of normality and adjustment for multiple comparisons
☐	☒	A full description of the statistical parameters including central tendency (e.g. means) or other basic estimates (e.g. regression coefficient) AND variation (e.g. standard deviation) or associated estimates of uncertainty (e.g. confidence intervals)
☒	☐	For null hypothesis testing, the test statistic (e.g. *F*, *t*, *r*) with confidence intervals, effect sizes, degrees of freedom and *P* value noted<br>*Give P values as exact values whenever suitable.*
☐	☒	For Bayesian analysis, information on the choice of priors and Markov chain Monte Carlo settings
☐	☒	For hierarchical and complex designs, identification of the appropriate level for tests and full reporting of outcomes
☐	☒	Estimates of effect sizes (e.g. Cohen's *d*, Pearson's *r*), indicating how they were calculated

Our web collection on statistics for biologists contains articles on many of the points above.

Software and code

Policy information about availability of computer code

Data collection	No software was used for data collection.
Data analysis	The code used to analyze the data is publicly available in an online repository (https://osf.io/5kzgd/). Analyses were performed using the R programming language (R version 4.4.1) and the brms package (version 2.22).

For manuscripts utilizing custom algorithms or software that are central to the research but not yet described in published literature, software must be made available to editors and reviewers. We strongly encourage code deposition in a community repository (e.g. GitHub). See the Nature Portfolio guidelines for submitting code & software for further information.

Data

Policy information about availability of data

All manuscripts must include a data availability statement. This statement should provide the following information, where applicable:

- Accession codes, unique identifiers, or web links for publicly available datasets
- A description of any restrictions on data availability
- For clinical datasets or third party data, please ensure that the statement adheres to our policy

We used data from existing studies or panels, some of which are restricted to protect the privacy of the study participants. We make a minimum dataset with the estimated test-retest correlations and inter-correlations from the primary data sources publicly available in an online repository (https://osf.io/5kzgd/). In what follows, we list information concerning each data source used, including the waves and information about the data access.

ADDHEALTH

Panel Name: National Longitudinal Study of Adolescent to Adult Health (ADDHEALTH)

Description: The National Study of Adolescent to Adult Health (Add Health; Harris & Udry, 2018) is an ongoing longitudinal study of U.S. adolescents in grades 7 through 12 during the 1994-1995 school year. The initial sample of participants was approximately 20,000 students who completed at home the study. Wave II included almost 15,000 follow-up-in-home interviews with adolescents from Wave I. Currently, five waves of data collection (1994-1995, 1996, 2001-2002, 2008-2009, 2016-2018) have been completed. There is a set of public-use datasets available that contain all the survey data for a subsample of the respondents.

More information at: https://addhealth.cpc.unc.edu/

Country/Countries: United States of America

Waves included in the analyses: Wave I - Wave V

Data collection period (of waves included in the analyses): 1994-2018

Dataset(s) version number/name: Waves 1-4 In-Home Questionnaire Data and Wave 5 Mixed-Mode Survey Data [Public-Use]

Harris, Kathleen Mullan, and Udry, J. Richard. National Longitudinal Study of Adolescent to Adult Health (Add Health), 1994-2018 [Public Use]. Carolina Population Center, University of North Carolina-Chapel Hill [distributor], Inter-university Consortium for Political and Social Research [distributor], 2022-08-09. https://doi.org/10.3886/ICPSR21600.v25

(specific data files: DS1, DS5, DS8, DS22, DS32)

Data access: The Add Health public-use dataset can be downloaed via the ICPSR Add Health page.

ALP

Panel Name: American Life Panel (ALP)

Description: The RAND American Life Panel (ALP) is a nationally representative, probability-based panel of 6,000 individuals ages 18 and older who speak English or Spanish. Participants are regularly completing surveys over the internet. The ALP has conducted more than 450 surveys covering diverse topics, such as financial decision-making, health decision-making, and numeracy.

More information at: https://www.rand.org/research/data/alp.html

Country/Countries: United States

Waves included in the analyses: ms2, ms48, ms50, ms130, ms133, ms167, ms169, ms186, ms189, ms197, ms246, ms260, ms284, ms342, ms349, ms315, ms352, ms472, ms474 (survey numbers of ALP public release data)

Data collection period (of waves included in the analyses): 2004-2017

Dataset(s) version number/name used for the analyses:

Well Being 2 - Health, Risk, Expenditures (ms2). Study page link

Well Being 48 - Cognition and Aging in the USA Internet Decision Making Survey [W01] (ms48). Study page link

Well Being 50 - Cognition and Aging in the USA Internet Decision Making Survey [W02] (ms50). Study page link

Well Being 130 - NYFED Module (ms130). Study page link

Well Being 133 - Health Expectations (ms133). Study page link

Well Being 167 - NYFED Module (ms167). Study page link

Well Being 169 - NYFED Module (ms169). Study page link

Well Being 186 - Long-term Care Insurance (ms186). Study page link

Well Being 189 - Savings Behavior (ms189). Study page link

Well Being 197 - Risk Aversion and Cognitive Ability (ms197). Study page link

Well Being 246 - Measuring Decision Quality (ms246). Study page link

Well Being 260 - Social Norms Marketing Interventions in Portfolio Choice (ms260). Study page link

Well Being 284 - National Financial Capability Study (ms284). Study page link

Well Being 315 - Decision Quality [Composite 1] (ms315). Study page link Well Being 342 - NBER [2] Followup to 341 Insurance (ms342). Study page link

Well Being 349 - Affordable Care Act (ms349). Study page link

Well Being 352 - Decision Quality [Composite 2] (ms352). Study page link

Well Being 472 - Copy of ms352 - Decision Quality [Composite 2] (ms472). Study page link

Well Being 474 - Copy of ms315 - Decision Quality [Composite 1] (ms474). Study page link

Data access: To access the ALP public release data one must first register as a user, more information is available on the Access ALP Data page The public release data can then be download via the ALP data catalogue

ANPS SPAIN

Panel Name: Study by Adema, Nikolka, Poutvaara, & Sunde (2022) (ANPS)

Description: Study conducted by Adema et al., (2022) published in Economics Letters. The study investigated the stability of risk preferences in the context of the COVID-19 pandemic. The survey was sent out to students attending one of nine universities located in four different countries (Czechia, India, Mexico, and Spain).

Adema, J., Nikolka, T., Poutvaara, P., & Sunde, U. (2022). On the stability of risk preferences: Measurement matters. Economics Letters, 210, 110172.

Country/Countries: Spain

Waves included in the analyses: W1 and W2

Data collection period (of waves included in the analyses): 2019-2021

Dataset(s) version number/name: Main data set available on Mendely Data (ANPS_main.csv)

Nikolka, Till; Poutvaara, Panu; Sunde, Uwe ; Adema, Joop (2021), "Supplementary Data to"On the Stability of Risk Preferences: Measurement Matters"", Mendeley Data, V2, doi: 10.17632/jzysn9brrb.2

Data access: Data can be directly downloaded from Mendely Data

ANPS CZECH REPUBLIC

Panel Name: Study by Adema, Nikolka, Poutvaara, & Sunde (2022) (ANPS)

Description: Study conducted by Adema et al., (2022) published in Economics Letters. The study investigated the stability of risk preferences in the context of the COVID-19 pandemic. The survey was sent out to students attending one of nine universities located in four different countries (Czechia, India, Mexico, and Spain).

Adema, J., Nikolka, T., Poutvaara, P., & Sunde, U. (2022). On the stability of risk preferences: Measurement matters. Economics Letters, 210, 110172.

Country/Countries: Czech Republic

Waves included in the analyses: W1 and W2

Data collection period (of waves included in the analyses): 2019-2021

Dataset(s) version number/name: Main data set available on Mendely Data (ANPS_main.csv)

Nikolka, Till; Poutvaara, Panu; Sunde, Uwe ; Adema, Joop (2021), "Supplementary Data to"On the Stability of Risk Preferences: Measurement Matters"", Mendeley Data, V2, doi: 10.17632/jzysn9brrb.2

Data access: Data can be directly downloaded from Mendely Data

BBRS-CH

Panel Name: Basel-Berlin Risk Study - Basel Sample (BBRS-CH)

Description: Study conducted by Frey et al., (2017) published in Science Advances (full reference below). The study investigated to what extent there is a general factor of risk preference, and whether risk preference can be regarded as a stable psychological trait. In this study, 1'507 healthy adults completed 39 risk-taking measures. A subsample completed a retest session. Data was collected in two cities (BBRS_Basel and BBRS_Berlin).

Frey, R., Pedroni, A., Mata, R., Rieskamp, J., & Hertwig, R. (2017). Risk preference shares the psychometric structure of major psychological traits. Science Advances, 3, e1701381.

Country/Countries: Switzerland

Waves included in the analyses: main (W1), retest_basel (W2)

Data collection period (of waves included in the analyses): 2015

Dataset(s) version number/name: From the main and retest_basel folders on the Open Science Framework repository - bart.csv, cct_overt.csv, dfd_perpers.csv, dfe_perpers.csv, lotteriesOvert.csv, mplBehavior.csv, mt.csv, participants.csv, quest_proc.csv

Data access: Data can be directly downloaded from the study's Open Science Framework repository

BBRS-DE

Panel Name: Basel-Berlin Risk Study - Berlin Sample (BBRS-DE)

Description: Study conducted by Frey et al., (2017) published in Science Advances (full reference below). The study investigated to what extent there is a general factor of risk preference, and whether risk preference can be regarded as a stable psychological trait. In this study, 1'507 healthy adults completed 39 risk-taking measures. A subsample completed a retest session. Data was collected in two cities (BBRS_Basel and BBRS_Berlin).

Frey, R., Pedroni, A., Mata, R., Rieskamp, J., & Hertwig, R. (2017). Risk preference shares the psychometric structure of major psychological traits. Science Advances, 3, e1701381.

Country/Countries: Germany

Waves included in the analyses: main (W1), retest_berlin (W2)

Data collection period (of waves included in the analyses): 2015

Dataset(s) version number/name: From the main and retest_berlin folders on the Open Science Framework repository - bart.csv, cct_overt.csv, dfd_perpers.csv, dfe_perpers.csv, lotteriesOvert.csv, mplBehavior.csv, mt.csv, participants.csv, quest_proc.csv

Data access: Data can be directly downloaded from the study's Open Science Framework repository

BES05

Panel Name: British Election Study 2005 (BES05)

Description: The British Election Study Nine-Wave Panel Survey, contains panel data from nine surveys conducted between the 2005 and 2010 general elections. The initial sample of participants who completed the survey online was around 8,000. The nine waves were collected as follows: three waves in 2005, conducted before the election campaign, during the campaign and post-election; one wave conducted in 2006, one in 2008 and one in 2009; and three waves conducted in 2010, before the election campaign, during the campaign and post-election. The surveys covered topics such as electoral issues, voting intentions and behaviour, as well as social and political attitudes.

More information on the UK Data Service study catalogue

Country/Countries: United Kingdom

Waves included in the analyses: Pre-Election 2005 (Internet) & Pre-Campaign 2010

Data collection period (of waves included in the analyses): 2005-2010

Dataset(s) version number/name: Stewart, M., Sanders, D., Whiteley, P. F., Clarke, H. (2014). British Election Study Nine-Wave Panel Survey, 2005-2010. [data collection]. 2nd Edition. UK Data Service. SN: 6607, http://doi.org/10.5255/UKDA-SN-6607-2

Data access: Data can be requested and downloaded via the UK Data Service study catalogue

BES14

Panel Name: The British Election Study 2014-2023 (BES14)

Description: The British Election Study Internet Panel is a longitudinal study on changes in attitudes and voting preferences in the United Kingdom. Surveys take place after every important election, helping researchers understand changing patterns of party support and election outcomes. The first survey was distributed in February 2014 to around 30,000 participants.

More information on the British Election Study webpage

Country/Countries: United Kingdom

Waves included in the analyses: Wave 1, Wave 7, Wave 8, and Wave 20

Data collection period (of waves included in the analyses): 2014-2020

Dataset(s) version number/name: Fieldhouse, E., J. Green, G. Evans, J. Mellon & C. Prosser, J. Bailey, R. de Geus, H. Schmitt and C. van der Eijk (2022) British Election Study Internet Panel Waves 1-23. DOI: 10.5255/UKDA-SN-8810-1

Data access: Data can be requested and downloaded via the British Election Study panel data catalogue

CMC

Panel Name: Crime in the Modern City. A Longitudinal Study of Juvenile Delinquency in Münster (CMC)

Description: This longitudinal study includes children and adolescents who attended school in Münster in the 7th grade in 2000. They were surveyed again in 2001, 2002 and 2003. The survey contains topics such as attitudes towards violence, crime and school as well as alcohol and drug consumption. The data was collected by the Institute for Criminal Research at the Westfälische Wilhelms-Universität.

More information at: https://search.gesis.org/research_data/ZA7480

Country/Countries: Germany

Waves included in the analyses: 2000, 2001, 2002 and 2003 (Wave 1 - Wave 4)

Data collection period (of waves included in the analyses): 2000-2003

Dataset(s) version number/name used for the analyses: Boers, Klaus, & Reinecke, Jost (2019). Crime in the Modern City. A Longitudinal Study of Juvenile Delinquency in Münster - Panel Study in 4 Waves (2000 - 2003). GESIS Data Archive, Cologne. ZA7480 Data file Version 1.0.0, https://doi.org/10.4232/1.13287.

Data access: Access to the data can be requested on the GESIS webpage

COGECON

Panel Name: Cognitive Economics Project (COGECON)

Description: The Cognitive Economics Project is a panel study focusing on the decision-making of aging citizens. This project was designed the increase the understanding of the cognitive bases of economic decision-making. Researchers collected data on topics such as: wealth, income, risk preference, affect, and cognition. The study was conducted from 2008 until 2017, yielding 5 waves.

More information at: https://ebp-projects.isr.umich.edu/CogEcon/

Country/Countries: United States

Waves included in the analyses: 2008, 2009, 2011 and 2013

Data collection period (of waves included in the analyses): 2008 - 2013

Dataset(s) version number/name used for the analyses: Cognitive Economics Study Data (the list of datasets can be viewed here)

CogEcon 2008-2009: Latest release - Jan 2012 (Ver 1.0)

CogEcon 2011: Latest release - Jan 2011 (Ver 1.2)

CogEcon 2013: Latest release - Jan 2013 (Ver 1.0)

Data access: Access to the data can be requested via the HRS Data Portal. Additional information can be found on the Access to Cognitive Economics Project Data page

DHS

Panel Name: DNB Household Survey (DNB)

Description: The DNB Household Survey, undertaken by CentERdata at Tilburg University since 1993, provides annual financial information on 2,000 Dutch households. The DNB Household Survey includes 6 questionnaires that cover topics such as : work, accommodation, health, assets and psychological constructs.

More information at: https://www.centerdata.eu/en/projects-by-centerdata/dnb-household-survey-dhs

Country/Countries: Netherlands

Waves included in the analyses: 1993-2022

Data collection period (of waves included in the analyses): 1993-2022

Dataset(s) version number/name: In this paper use is made of data of the DNB Household Survey administered by Centerdata (Tilburg University, The Netherlands). We used data from the PSY and HHI modules for years 1993-2022, for 1993 also used data from the WRK module

Data access: Access to the data can be requested via the CentERdata's website. The data sets can then be downloaded on the DHS data access website

DRICHOUTIS

Panel Name: Study by Drichoutis & Vassilopoulos (2019) (DRICHOUTIS)

Description: Study conducted by Drichoutis & Vassilopoulos (2019) published in Journal of Economics & Management Strategy. The study investigated the intertemporal stability of six measures over the course of 3 waves. The survey included assessments of risk, time, and social preferences.

Drichoutis, A. C., & Vassilopoulos, A. (2021). Intertemporal stability of survey-based measures of risk and time preferences. Journal of Economics & Management Strategy, 30(3), 655-683.

Country/Countries: Greece

Waves included in the analyses: W1, W2, and W3

Data collection period (of waves included in the analyses): 2013-2015

Dataset(s) version number/name: data.dta file from Open Science Repository

Data access: Open Science Repository

ENKAVI

Panel Name: Study by Enkavi et al., (2019) (ENKAVI)

Description: Study conducted by Enkavi et al., (2019) published in Proceedings of the National Academy of Sciences (full reference below). The paper examined the test-retest reliability of various self-report and behavioral measures of self-regulation. Retest data was collected from 150 participants who were a subset of a sample from another study (Eisenberg et al., 2018). Data was collected between 2016 and 2017 using Amazon MTurk.

Enkavi, A. Z., Eisenberg, I. W., Bissett, P. G., Mazza, G. L., MacKinnon, D. P., Marsch, L. A., & Poldrack, R. A. (2019). Large-scale analysis of test-retest reliabilities of self-regulation measures. Proceedings of the National Academy of Sciences of the United States of America, 116(12), 5472–5477. https://doi.org/10.1073/pnas.1818430116

Eisenberg, I. W., et al. (2018). Applying novel technologies and methods to inform the ontology of self-regulation. Behaviour research and therapy, 101, 46–57. https://doi.org/10.1016/j.brat.2017.09.014

Country/Countries: United States

Waves included in the analyses: Wave 1 and Wave 2

Data collection period (of waves included in the analyses): 2016-2017

Dataset(s) version number/name: Complete_02-16-2019 (variables_exhaustive.csv, alcohol_drugs.csv, demographics.csv and demographics_survey.csv) and Retest_02-16-2019 (variables_exhaustive.csv, alcohol_drugs.csv, demographics.csv and demographics_survey.csv)

Data access: GitHub Repository

FICR

Panel Name: Financial Crisis: A Longitudinal Study of Public Response (FICR)

Description: The Financial Crisis: A Longitudinal Study of Public Response (FICR) was conducted to understand hoe peopel percieved risk during the economic crisis in 2008. Eight (online) surveys were sent out between late September 2008 and August 2011. At least 600 respondents participated in each survey, with 325 completing all eight surveys. It ocntaned questions focused on risk perception, nagetive emotions, and confidence in national leaders.

Burns, William. Financial Crisis: A Longitudinal Study of Public Response. Ann Arbor, MI: Inter-university Consortium for Political and Social Research [distributor], 2016-01-25. https://doi.org/10.3886/ICPSR36341.v1

Country/Countries: United States

Waves included in the analyses: Wave 3, Wave 5-7

Data collection period (of waves included in the analyses): 2008-2009

Dataset(s) version number/name:

DS1 Financial Crisis: A Longitudinal Study of Public Response

Data access: Data can be downloaded via the ICPSR page of the study

GCOE (China Urban Sample)

Panel Name: Preference Parameters Study - China Urban Sample (GCOE_CN)

Description: The Preference Parameters Study of Osaka University is an extensive panel study conducted in 4 different countries (Japan, United States, China and India). The study includes measures to assess time preference, risk aversion, habit formation as well as externality.

For the survey in the Chinese urban area, the panel survey was conducted in six cities (Beijing, Shanghai, Guangzhou, Chengdu, Wuhan, Shenyang) since 2009 with a sample of men and women aged 20-69 years old.

More information at: https://www.iser.osaka-u.ac.jp/survey_data/eng_panelsummary.html

Country/Countries: China

Waves included in the analyses: 2009 and 2010

Data collection period (of waves included in the analyses): 2009-2010

Dataset(s) version number/name used for the analyses: This research utilizes the micro data from the Preference Parameters Study of Osaka University's 21st Century COE Program 'Behavioral Macro Macro-Dynamic s Based on Surveys and Experiments', its Global COE project 'Human Behavior and Socioeconomic Dynamics' and JSPS KAKENHI 15H05728 'Behavioral Behavioral-Economic Analysis of Long Long-Run Stagnation'.

Specifically, we used the following data sets: 2009Data_CHINA and 2010Data_CHINA.

Data access: Access to the data can be requested via the form available on the Data Application page

GCOE (India Rural Sample)

Panel Name: Preference Parameters Study - India Rural Sample (GCOE_IN_RUR)

Description: The Preference Parameters Study of Osaka University is an extensive panel study conducted in 4 different countries (Japan, United States, China and India). The study includes measures to assess time preference, risk aversion, habit formation as well as externality.

For the survey in the Indian rural areas, the panel survey was conducted annually from 2012 to 2013. Samples of men and women aged 20-69 living in the rural areas of four cities (Delhi, Mumbai, Bangalore, Calcutta) were interviewed.

More information at: https://www.iser.osaka-u.ac.jp/survey_data/eng_panelsummary.html

Country/Countries: India

Waves included in the analyses: 2012 and 2013

Data collection period (of waves included in the analyses): 2012-2013

Dataset(s) version number/name used for the analyses: This research utilizes the micro data from the Preference Parameters Study of Osaka University's 21st Century COE Program 'Behavioral Macro Macro-Dynamic s Based on Surveys and Experiments', its Global COE project 'Human Behavior and Socioeconomic Dynamics' and JSPS KAKENHI 15H05728 'Behavioral Behavioral-Economic Analysis of Long Long-Run Stagnation'.

Specfically we used the following data sets:2012Data_RURAL_INDIA, and 2013Data_RURAL_INDIA

Data access: Access to the data can be requested via the form available on the Data Application page

GCOE (India Urban Sample)

Panel Name: Preference Parameters Study - India Urban Sample (GCOE_IN)

Description: The Preference Parameters Study of Osaka University is an extensive panel study conducted in 4 different countries (Japan, United States, China and India). The study includes measures to assess time preference, risk aversion, habit formation as well as externality.

For the survey in the India urban areas, the panel survey has been conducted annually from 2009 to 2013. Samples of men and women aged 20-69 living in urban areas of six cities (Delhi, Mumbai, Bangalore, Chennai, Calcutta, Hyderabad) were interviewed.

More information at: https://www.iser.osaka-u.ac.jp/survey_data/eng_panelsummary.html

Country/Countries: India

Waves included in the analyses: 2009-2013

Data collection period (of waves included in the analyses): 2009-2013

Dataset(s) version number/name used for the analyses: This research utilizes the micro data from the Preference Parameters Study of Osaka University's 21st Century COE Program 'Behavioral Macro Macro-Dynamic s Based on Surveys and Experiments', its Global COE project 'Human Behavior and Socioeconomic Dynamics' and JSPS KAKENHI 15H05728 'Behavioral Behavioral-Economic Analysis of Long Long-Run Stagnation'.

Specfically we used the following data sets:2009Data_INDIA, 2010Data_INDIA, 2011Data_INDIA, 2012Data_URBAN_INDIA, 2013Data_URBAN_INDIA

Data access: Access to the data can be requested via the form available on the Data Application page

GCOE (Japan Sample)

Panel Name: Preference Parameters Study - Japan Sample (GCOE_JP)

Description: The Preference Parameters Study of Osaka University is an extensive panel study conducted in 4 different countries (Japan, United States, China and India). The study includes measures to assess time preference, risk aversion, habit formation as well as externality.

The panel survey in Japan has been conducted annually from 2003 until 2018 using a random sample of men and women aged 20-69 years old by a self-administered placement method.

More information at: https://www.iser.osaka-u.ac.jp/survey_data/eng_panelsummary.html

Country/Countries: Japan

Waves included in the analyses: 2003-2018

Data collection period (of waves included in the analyses): 2003-2018

Dataset(s) version number/name used for the analyses: This research utilizes the micro data from the Preference Parameters Study of Osaka University's 21st Century COE Program 'Behavioral Macro Macro-Dynamic s Based on Surveys and Experiments', its Global COE project 'Human Behavior and Socioeconomic Dynamics' and JSPS KAKENHI 15H05728 'Behavioral Behavioral-Economic Analysis of Long Long-Run Stagnation'.

Specfically we used the following data sets:2003Data_JAPAN, 2004Data_JAPAN, 2005Data_JAPAN, 2006Data_JAPAN, 2007Data_JAPAN, 2008Data_JAPAN, 2009Data_JAPAN, 2010Data_JAPAN, 2011Data_JAPAN, 2012Data_JAPAN, 2013Data_JAPAN, 2016Data_JAPAN, 2017Data_JAPAN, 2018Data_JAPAN

Data access: Access to the data can be requested via the form available on the Data Application page

GCOE (USA Sample)

Panel Name: Preference Parameters Study - USA Sample (GCOE_USA)

Description: The Preference Parameters Study of Osaka University is an extensive panel study conducted in 4 different countries (Japan, United States, China and India). The study includes measures to assess time preference, risk aversion, habit formation as well as externality. The panel survey for the GCOE USA sample has been conducted annually from 2005 to 2013 using a random sample of men and women aged 18-99 years old.

More information at: https://www.iser.osaka-u.ac.jp/survey_data/eng_panelsummary.html

Country/Countries: United States of America

Waves included in the analyses: 2005-2013

Data collection period (of waves included in the analyses): 2005-2013

Dataset(s) version number/name used for the analyses: This research utilizes the micro data from the Preference Parameters Study of Osaka University's 21st Century COE Program 'Behavioral Macro Macro-Dynamic s Based on Surveys and Experiments', its Global COE project 'Human Behavior and Socioeconomic Dynamics' and JSPS KAKENHI 15H05728 'Behavioral Behavioral-Economic Analysis of Long Long-Run Stagnation'.

Specfically we used the following data sets:2005Data_USA, 2006Data_USA, 2007Data_USA, 2008Data_USA, 2009Data_USA, 2010Data_USA, 2011Data_USA, 2012Data_USA, 2013Data_USA.

Data access: Access to the data can be requested via the form available on the Data Application page

GIP

Panel Name: German Internet Panel (GIP)

Description: The German Internet Panel (GIP) is a longitudinal study developped by the University of Mannheim and the central infrastructure project of the Collaborative Research Center (SFB) 884 "Political Economy of Reforms", which is funded by the German Research Foundation (DFG). The panel studies attitudes and preferences relevant in political and economic decision-making processes. Approximately 4,000 people in Germany are regularly interviewed online on a variety of topics.

More information at: https://www.uni-mannheim.de/en/gip/

Country/Countries: Germany

Waves included in the analyses: W9, W14, W56

Data collection period (of waves included in the analyses): 2014 and 2021

Dataset(s) version number/name used for the analyses: This study uses data from the wave(s) 9, 14, and 56 of the German Internet Panel (GIP; DOI: [10.4232/1.12615; 10.4232/1.12620; 10.4232/1.13945]; Blom et al. (2014)). A study description can be found in Blom et al. (2015). The GIP is funded by the German Research Foundation (DFG) as part of the Collaborative Research Center 884 (SFB 884; Project Number 139943784; Project Z1).

Blom, A. G., Gathmann, C., and Krieger, U. (2015). Setting Up an Online Panel Representative of the General Population: The German Internet Panel. Field Methods, 27(4), 391–408. DOI: 10.1177/1525822X15574494

Data access: Instructions on how to access the data can be found on the Data Use page

GLES-LT

Panel Name: GLES Panel 2016-2021 (Long-Term Panel; GLES-LT)

Description: The German Longitudinal Election Study (GLES) collects data on the political attitudes and behaviour of voters and candidates. It is carried in close cooperation with the German Society for Electoral Studies (DGfW) and the GESIS – Leibniz Institute for the Social Sciences. The GLES Panel conducts surveys before and after the German federal elections, allowing to track intra-individual changes in political attitudes and behaviors. Topics in the survey include political involvement, political attitudes, personality, and voting behaviour.

More information is available on the GLES website

Country/Countries: Germany

Waves included in the analyses: Wave 1, Wave a1, Wave a2, Wave 13, Wave 14 and Wave 15

Data collection period (of waves included in the analyses): 2016 - 2021

Dataset(s) version number/name: GLES (2021). GLES Panel 2016-2021, Waves 1-15. GESIS Data Archive, Cologne. ZA6838 Data file Version 5.0.0, https://doi.org/10.4232/1.13783.

Data access: After registering on the GESIS website, the data can be downloaded directly via the page of each data set

GLES ST

Panel Name: German Longitudinal Election Study - Short term Campaign (GLES-ST)

Description: The German Longitudinal Election Study (GLES) collects data on the political attitudes and behaviour of voters and candidates. It is carried in close cooperation with the German Society for Electoral Studies (DGfW) and the GESIS – Leibniz Institute for the Social Sciences. The Campaign Panel 2013-2017 is a repeat survey of internet-users eligible to vote in the election to the German Bundestag in 2013 and 2017. It allows to track intra-individual changes in political attitudes and behaviors. Topics in the survey include political involvement, political attitudes, personality, and voting behaviour.

More information is available on the GLES website

Country/Countries: Germany

Waves included in the analyses: Wave 9 and Wave 10

Data collection period (of waves included in the analyses): 2015 - 2016

Dataset(s) version number/name: GLES (2018). Repeatedly questioned respondents of the Short-term Campaign Panel 2013 and 2017 (GLES). GESIS Data Archive, Cologne. ZA6827 Data file Version 1.0.0, https://doi.org/10.4232/1.13129.

Data access: After registering on the GESIS website, the data can be downloaded directly via the page of each data set

HILDA

Panel Name: Household, Income and Labour Dynamics in Australia (HILDA)

Description: The Household, Income and Labour Dynamics in Australia (HILDA) Survey is a household-based panel study that collects information about economic and personal well-being, labour market dynamics and family life of participants. Since 2001, the study has been following more than 17,000 Australian participants each year.

More information at: https://melbourneinstitute.unimelb.edu.au/hilda

Country/Countries: Australia

Waves included in the analyses: Wave 1 - Wave 21

Data collection period (of waves included in the analyses): 2001-2021

Dataset(s) version number/name used for the analyses: This paper uses unit record data from Household, Income and Labour Dynamics in Australia Survey (HILDA). HILDA conducted by the Australian Government Department of Social Services (DSS). The findings and views reported in this paper, however, are those of the author[s] and should not be attributed to the Australian Government, DSS, or any of DSS' contractors or partners. DOI: doi:10.26193/KXNEBO

Department of Social Services; Melbourne Institute of Applied Economic and Social Research, 2022, "The Household, Income and Labour Dynamics in Australia (HILDA) Survey, GENERAL RELEASE 21 (Waves 1-21)", doi:10.26193/KXNEBO, ADA Dataverse, V3

Data access: Data can be requested and downloaded via the National Centre for Longitudinal Data Dataverse.

HRS

Panel Name: Health and Retirement Study (HRS)

Description: The Health and Retirement Study (HRS) is a longitudinal panel study that surveys a representative sample of approximately 20,000 individuals of 50+ years old living in the United States of America. A new cohort of individuals between 51 and 56 years old is added every 6 years. Individuals and their spouses/partners are followed until their death. The survey focuses on financial and social factors. Data have been collected biannually since 1992.

The HRS (Health and Retirement Study) is sponsored by the National Institute on Aging (grant number NIA U01AG009740) and is conducted by the University of Michigan.

More information at: https://hrs.isr.umich.edu/about

Country/Countries: United States of America

Waves included in the analyses: Waves 1992 - 2020

Data collection period (of waves included in the analyses): 1992 - 2021

Dataset(s) version number/name used for the analyses:

Health and Retirement Study, (1992 HRS Core: Latest Release - Sep 2004 (Final V2.0)) public use dataset. Produced and distributed by the University of Michigan with funding from the National Institute on Aging (grant number NIA U01AG009740). Ann Arbor, MI, (2004).

Health and Retirement Study, (1994 HRS Core: Latest Release - Sep 2004 (Final V2.0)) public use dataset. Produced and distributed by the University of Michigan with funding from the National Institute on Aging (grant number NIA U01AG009740). Ann Arbor, MI, (2004).

Health and Retirement Study, (1996 HRS Core: Latest Release - Mar 2007 (Final V4.00)) public use dataset. Produced and distributed by the University of Michigan with funding from the National Institute on Aging (grant number NIA U01AG009740). Ann Arbor, MI, (2007).

Health and Retirement Study, (1998 HRS Core: Latest Release - Nov 2003 (Final V2.3)) public use dataset. Produced and distributed by the University of Michigan with funding from the National Institute on Aging (grant number NIA U01AG009740). Ann Arbor, MI, (2003).

Health and Retirement Study, (2000 HRS Core: Latest Release - Apr 2004 (Final V1.0)) public use dataset. Produced and distributed by the University of Michigan with funding from the National Institute on Aging (grant number NIA U01AG009740). Ann Arbor, MI, (2004).

Health and Retirement Study, (2002 HRS Core: Latest Release - Jul 2006 (Final V2.0)) public use dataset. Produced and distributed by the University of Michigan with funding from the National Institute on Aging (grant number NIA U01AG009740). Ann Arbor, MI, (2006).

Health and Retirement Study, (2004 HRS Core: Latest Release - May 2016 (Final V1.0)) public use dataset. Produced and distributed by the University of Michigan with funding from the National Institute on Aging (grant number NIA U01AG009740). Ann Arbor, MI, (2016).

Health and Retirement Study, (2006 HRS Core: Latest Release - Aug 2021 (Final V4.0)) public use dataset. Produced and distributed by the University of Michigan with funding from the National Institute on Aging (grant number NIA U01AG009740). Ann Arbor, MI, (2021).

Health and Retirement Study, (2008 HRS Core: Latest Release - Dec 2014 (Final V3.0)) public use dataset. Produced and distributed by the University of Michigan with funding from the National Institute on Aging (grant number NIA U01AG009740). Ann Arbor, MI, (2014).

Health and Retirement Study, (2010 HRS Core: Latest Release - Aug 2021 (Final V6.0)) public use dataset. Produced and distributed by the University of Michigan with funding from the National Institute on Aging (grant number NIA U01AG009740). Ann Arbor, MI, (2021).

Health and Retirement Study, (2012 HRS Core: Latest Release - Mar 2020 (Final V3.0)) public use dataset. Produced and distributed by the University of Michigan with funding from the National Institute on Aging (grant number NIA U01AG009740). Ann Arbor, MI, (2020).

Health and Retirement Study, (2014 HRS Core: Latest Release - Dec 2017 (Final V2.0)) public use dataset. Produced and distributed by the University of Michigan with funding from the National Institute on Aging (grant number NIA U01AG009740). Ann Arbor, MI, (2017).

Health and Retirement Study, (2016 HRS Core: Latest Release - Dec 2019 (Final V2.0)) public use dataset. Produced and distributed by the University of Michigan with funding from the National Institute on Aging (grant number NIA U01AG009740). Ann Arbor, MI, (2019).

Health and Retirement Study, (2018 HRS Core: Latest Release - Dec 2019 (Early V1.0)) public use dataset. Produced and distributed by the University of Michigan with funding from the National Institute on Aging (grant number NIA U01AG009740). Ann Arbor, MI, (2019). These data have not been cleaned and may contain errors that will be corrected in the Final Public Release version of the dataset.

Health and Retirement Study, (2020 HRS Core: Latest Release - May 2023 (Final V1.0)) public use dataset. Produced and distributed by the University of Michigan with funding from the National Institute on Aging (grant number NIA U01AG009740). Ann Arbor, MI, (2023).

Data access: Access to the data can be requested via the HRS Data Portal.

IFLS

Panel Name: Indonesia Family Life Survey (IFLS)

Description: The Indonesian Family Life Survey (IFLS) is an on-going longitudinal survey in Indonesia. The sample consists of over 30,000 individuals. The first wave was conducted in 1993/94, then again in 1997/98. The third waves was conducted in 2000, the fourth wave in 2007/2008, and the fifth wave in 2014-15. Survey items include: personality, well-being, positive and negative affect, health status, and education.

More information at: https://www.rand.org/well-being/social-and-behavioral-policy/data/FLS/IFLS.html

Strauss, J., F. Witoelar, and B. Sikoki. "The Fifth Wave of the Indonesia Family Life Survey (IFLS5): Overview and Field Report". March 2016. WR-1143/1-NIA/NICHD. Papers that use IFLS4 (2007):

Strauss, J., F. Witoelar, B. Sikoki and A.M. Wattie. "The Fourth Wave of the Indonesian Family Life Survey (IFLS4): Overview and Field Report". April 2009. WR-675/1-NIA/NICHD. Papers that use IFLS3 (2000):

Strauss, J., K. Beegle, B. Sikoki, A. Dwiyanto, Y. Herawati and F. Witoelar. "The Third Wave of the Indonesia Family Life Survey (IFLS): Overview and Field Report", March 2004. WR-144/1-NIA/NICHD.

Frankenberg, E. and D. Thomas. "The Indonesia Family Life Survey (IFLS): Study Design and Results from Waves 1 and 2." March 2000. RAND, Santa Monica, CA. DRU-2238/1-NIA/NICHD. Papers that use IFLS1 (1993):

Frankenberg, E. and L. Karoly. "The 1993 Indonesian Family Life Survey: Overview and Field Report." November, 1995. RAND, Santa Monica, CA.

Country/Countries: Indonesia

Waves included in the analyses: Waves 1-5

Data collection period (of waves included in the analyses): 1993-2015

Dataset(s) version number/name:

Wave 1: hh93b3. (Individual adult)

Wave 2: hh97b3 (Individual adult)

Wave 3: hh00_b3a_dta and hh00_b3b_dta (Individual adult Part A & B)

Wave 4: hh07_b3a_dta and hh07_b3b_dta (Individual adult Part A & B)

Wave 5: hh14_b3a_dta and hh14_b3b_dta (Individual adult Part A & B)

Data access: Data can be requested and downloaded via the study page on the RAND website

JSTAR

Panel Name: Japanese Study of Aging and Retirement (JSTAR)

Description: The Japanese Study of Aging and Retirement (JSTAR) was conducted by the Research Institute of Economy, Trade and Industry (RIETI), Hitotsubashi University, and the University of Tokyo. The Japanese Study of Aging and Retirement (JSTAR) is a panel survey of elderly people (+50 years old) conducted by the Research Institute of Economy, Trade and Industry of the Hitotsubashi University, and the University of Tokyo. It is a panel survey that collects data on people's economic, social, and health conditions. In addition, the survey is designed to ensure comparability with other retirement surveys such as the Health and Retirement Study (HRS) from the U.S.A.

More information at: https://www.rieti.go.jp/en/projects/jstar/

Country/Countries: Japan

Waves included in the analyses: Wave 2007, Wave 2009, Wave 2011, Wave 2013

Data collection period (of waves included in the analyses): 2007-2013

Dataset(s) version number/name used for the analyses:

2007 JSTAR (Japanese Study of Aging and Retirement)—High Level

2009 JSTAR (Japanese Study of Aging and Retirement)—High Level

2011 JSTAR (Japanese Study of Aging and Retirement)—High Level

2013 JSTAR (Japanese Study of Aging and Retirement)—High Level

Data access: Access to the data can be requested via the Research Institute of Economy, Trade and Industry (RIETY) JSTAR study page.

KLIPS

Panel Name: Korean Labor & Income Panel Study (KLIPS)

Description: Korean Labor & Income Panel Study is a longitudinal survey of the income of households. The survey was launched by the Korea Labor Institute in 1998, and has been been collected data since then, and is currently on its 25th wave. Data is collected from 5'000 households, which includes over 13'000 individuals. Contents of the survey include questions on education, employment, housing, leisure, decision-making, and atitudes towards life.

More information at: https://www.kli.re.kr/klips_eng

Country/Countries: South Korea

Waves included in the analyses: Wave 7, Wave 10, Wave 23-25

Data collection period (of waves included in the analyses): 2007-2024

Dataset(s) version number/name used for the analyses:

1-25th wave SPSS version
Data access: Access to the data can be requested via the Korea Labor Institute (KLI) KLIPS page.

LIKS

Panel Name: Life in Kyrgyzstan Study (LIKS)

Description: The 'Life in Kyrgyzstan' Study is a longitudinal survey of households and individuals in Kyrgyzstan. It tracks the same 3,000 households and 8,000 individuals over time in all seven Kyrgyz regions (oblasts) and the two cities of Bishkek and Osh. The data are representative at the national and regional level (East, West, North, South). The survey interviews all adult household members about household demographics, assets, expenditure, migration, employment, agricultural markets, shocks, social networks, subjective well-being, and many other topics. The survey was first conducted in 2010 and it has been repeated five times in 2011, 2012, 2013, 2016, and 2019.

More information at: https://lifeinkyrgyzstan.org/about/

Country/Countries: Kyrgyzstan

Waves included in the analyses: Wave 2010, Wave 2011, Wave 2012, Wave 2013, Wave 2016, Wave 2019

Data collection period (of waves included in the analyses): 2010-2019

Dataset(s) version number/name used for the analyses:

Brück, T., D. Esenaliev, A. Kroeger, A. Kudebayeva, B. Mirkasimov and S. Steiner (2014): "Household Survey Data for Research on Well-Being and Behavior in Central Asia". Journal of Comparative Economics, vol. 42, no. 3, pp. 819-35.

Leibniz Institute of Vegetable and Ornamental Crops (IGZ), Germany; University of Central Asia (UCA), Kyrgyzstan; Stockholm International Peace Research Institute (SIPRI), Sweden; German Institute for Economic Research (DIW Berlin). Research Data Center of IZA (IDSC). Version 1.0, doi:10.15185/izadp.7055.1 Downloaded the Lik_2022 file

Data access: Access to the data can be requested via the International Data Service Center of the Institute for Study of Labour (IDSC IZA) Data Set Repository.

LSVAW-M

Panel Name: Longitudinal Study of Violence Against Women: Victimization and Perpetration Among College Students in a State-Supported University in the United States (LSVAW - Men sample)

Description: A longitudinal study aimed at investigating the developmental antecedents of physical and sexual violence against young women. The survey included questions about the respondent's personality, dating behaviour, and other social behaviour. The sample was constituted of males that woman who had responded to the survey reported having had sexual intercourse with.

More information on the ICPSR website

Country/Countries: United States of America

Waves included in the analyses: Waves 1-5

Data collection period (of waves included in the analyses): 1991-1995

Dataset(s) version number/name:

White, Jacquelyn W., University of North Carolina-Greensboro, and Humphrey, John A. Longitudinal Study of Violence Against Women: Victimization and Perpetration Among College Students in a State-Supported University in the United States, 1990-1995. Inter-university Consortium for Political and Social Research [distributor], 2015-09-11. https://doi.org/10.3886/ICPSR03212.v1

Specific file: DS2 Male Data

Data access: Data can be downloaded via the ICPSR page of the study

LSVAW-W

Panel Name: Longitudinal Study of Violence Against Women: Victimization and Perpetration Among College Students in a State-Supported University in the United States (LSVAW - Women sample)

Description: A longitudinal study aimed at investigating the developmental antecedents of physical and sexual violence against young women. Data for the female sample were collected when women were aged 18 years old, and again when they were 19, 20, 21, and 22 years old. The survey included questions about the respondent's personality, dating behaviour, and other social behaviour.

More information on the ICPSR website

Country/Countries: United States of America

Waves included in the analyses: Waves 1-5

Data collection period (of waves included in the analyses): 1990-1994

Dataset(s) version number/name:

White, Jacquelyn W., University of North Carolina-Greensboro, and Humphrey, John A. Longitudinal Study of Violence Against Women: Victimization and Perpetration Among College Students in a State-Supported University in the United States, 1990-1995. Inter-university Consortium for Political and Social Research [distributor], 2015-09-11. https://doi.org/10.3886/ICPSR03212.v1

Specific file: DS1 Female Data

Data access: Data can be downloaded via the ICPSR page of the study

MEPS

Panel Name: Medical Expenditure Panel Survey (MEPS)

Description: The Medical Expenditure Panel Survey (MEPS) is a set of large-scale surveys of families and individuals, their medical providers, and employers across the United States of America. MEPS collects data on the specific health services that Americans use, how frequently they use them, the cost of these services, and how they are paid for, as well as data on the cost, scope, and breadth of health insurance held by and available to U.S. workers. The number of families recruited have ranged from around 8,000 to 15,000. The survey was launched in 1996 and continues to collect data until today on an annual basis. Data is also collected from respondents who participated in two surveys (approx. a year a part).

More information on the MEPS website

Country/Countries: United States of America

Waves included in the analyses: See waves listed below

Data collection period (of waves included in the analyses): 2000-2017

Dataset(s) version number/name: See PUF No. and File name list below

The following data was obtained from the Agency for Healthcare Research and Quality (AHRQ) and the Medical Expenditure Panel Survey

HC-202: MEPS Panel 21 Longitudinal Data File

HC-193: MEPS Panel 20 Longitudinal Data File

HC-183: MEPS Panel 19 Longitudinal Data File

HC-172: MEPS Panel 18 Longitudinal Data File

HC-164: MEPS Panel 17 Longitudinal Data File

HC-156: MEPS Panel 16 Longitudinal Data File

HC-148: MEPS Panel 15 Longitudinal Data File

HC-139: MEPS Panel 14 Longitudinal Data File

HC-130: MEPS Panel 13 Longitudinal Data File

HC-122: MEPS Panel 12 Longitudinal Data File

HC-114: MEPS Panel 11 Longitudinal Data File

HC-106: MEPS Panel 10 Longitudinal Data File

HC-098: MEPS Panel 9 Longitudinal Data File

HC-086: MEPS Panel 8 Longitudinal Data File

HC-080: MEPS Panel 7 Longitudinal Data File

HC-071: MEPS Panel 6 Longitudinal Data File

HC-065: MEPS Panel 5 Longitudinal Data File

Data access: Data can be downloaded via the MEPS Longitudinal Data File page

MIDJA

Panel Name: Midlife in Japan (MIDJA)

Description: Midlife in Japan is a longitudinal study conducted with the aim of comparing the results to the Midlife in the United States sample (MIDUS). Baseline and follow-up survey responses were collected from a sample of Japanese adults. The MIDJA survey contains a similar set of questions as MIDUS, it is interested in the association between psycho-social factors and health.

More information on the MIDJA page of the MIDUS website

Country/Countries: Japan

Waves included in the analyses: MIDJA 1, MIDJA 2

Data collection period (of waves included in the analyses): 2008, 2012

Dataset(s) version number/name:

MIDJA 1: Ryff, Carol D., Kitayam, Shinobu, Karasawa, Mayumi, Markus, Hazel, Kawakami, Norito, and Coe, Christopher. Survey of Midlife in Japan (MIDJA), April-September 2008. Inter-university Consortium for Political and Social Research [distributor], 2018-03-09. https://doi.org/10.3886/ICPSR30822.v3

MIDJA 2: Ryff, Carol D., Kitayama, Shinobu, Karasawa, Mayumi, Markus, Hazel, Kawakami, Norito, and Coe, Christopher. Survey of Midlife in Japan (MIDJA 2), May-October 2012. Inter-university Consortium for Political and Social Research [distributor], 2018-02-19. https://doi.org/10.3886/ICPSR36427.v3

Data access: Data can be downloaded via the MIDUS collectica platform

MIDUS

Panel Name: Midlife in the United States (MIDUS)

Description: Midlife in the United States is a national longitudinal study that begun in 1995. It includes data from over 12,000 individuals, and investigates the role of different factors (e.g., behavioral, psychological) on age-related differences in physical and mental health.

More information on the MIDUS website

Country/Countries: United States of America

Waves included in the analyses: MIDUS 1 (Core), MIDUS 2 (Core), MIDUS 3 (Core)

Data collection period (of waves included in the analyses): 1995-2013

Dataset(s) version number/name:

MIDUS 1 - Project 1 (DS1 Main, Siblings and Twin Data): Brim, Orville Gilbert, Baltes, Paul B., Bumpass, Larry L., Cleary, Paul D., Featherman, David L., Hazzard, William R., ... Shweder, Richard A. Midlife in the United States (MIDUS 1), 1995-1996. Inter-university Consortium for Political and Social Research [distributor], 2020-09-28. https://doi.org/10.3886/ICPSR02760.v19

MIDUS 2 - Project 1: Ryff, Carol D., Almeida, David M., Ayanian, John Z., Carr, Deborah S., Cleary, Paul D., Coe, Christopher, ... Williams, David R. Midlife in the United States (MIDUS 2), 2004-2006. Inter-university Consortium for Political and Social Research [distributor], 2021-09-15. https://doi.org/10.3886/ICPSR04652.v8
Obtained via the MIDUS collectica platform

MIDUS 3 - Project 1 (DS1 Aggregate Data): Ryff, Carol, Almeida, David, Ayanian, John, Binkley, Neil, Carr, Deborah S., Coe, Christopher, ... Williams, David. Midlife in the United States (MIDUS 3), 2013-2014 . Ann Arbor, MI: Inter-university Consortium for Political and Social Research [distributor], 2019-04-30. https://doi.org/10.3886/ICPSR36346.v7

Data access: Data can be downloaded via the MIDUS collectica platform

NLSY79

Panel Name: National Longitudinal Survey of Youth-1979 (NLSY79)

Description: The NLSY79 is a longitudinal project that studies the lives of young Americans born between 1957-64. The project started in 1979 and included 12,686 respondents between the ages of 14 and 22. Afterwards certain participants were dropped from the project, leaving 9,964 respondents. Data are available from the 1979 to 2020 survey year.

The NLSY97 survey is sponsored and directed by the U.S. Bureau of Labor Statistics and and managed by the Center for Human Resource Research (CHRR) at The Ohio State University. Interviews are conducted by the National Opinion Research Center (NORC) at the University of Chicago.

More information at: https://www.nlsinfo.org/content/cohorts/nlsy79

Country/Countries: Unites States of America

Waves included in the analyses: 1982 - 2018

Data collection period (of waves included in the analyses): 1982 - 2018

Dataset(s) version number/name used for the analyses:

Bureau of Labor Statistics, U.S. Department of Labor. National Longitudinal Survey of Youth 1979 cohort, 1979-2016 (rounds 1-27). Produced and distributed by the Center for Human Resource Research (CHRR), The Ohio State University. Columbus, OH: 2019.

We created a dataset by selecting the relevant variables from the surveys using the NLS Investigator tool (dataset created on February 7th, 2024)

Data access: Data can be accessed and directly downloaded via the NLS investigator tool

NLSY79-CYA

Panel Name: National Longitudinal Survey of Youth 1979 - Child and Young Adult (NLSY79_CYA)

Description: The NLSY79 Child and Young Adult cohort is a longitudinal project that follows the biological children of the women in the National Longitudinal Survey of Youth 1979. The Child Survey began in 1986, collecting child-specific information every two years. The Youth Survey began in 1994, interviewing children ages 15 and older on topics such as education, health, and employment.

The Children of the NLSY79 survey is sponsored and directed by the U.S. Bureau of Labor Statistics and the National Institute for Child Health and Human Development. The survey is managed by the Center for Human Resource Research (CHRR) at The Ohio State University and interviews are conducted by the National Opinion Research Center (NORC) at the University of Chicago.

More information at: https://www.nlsinfo.org/content/cohorts/nlsy79-children

Country/Countries: Unites States of America

Waves included in the analyses: 1988 - 2014 (Child Self-Report) and 1994 - 2020 (Young Adult self-report)

Data collection period (of waves included in the analyses): 1988 - 2014 (Child Self-Report) and 1994 - 2020 (Young adult self-report)

Dataset(s) version number/name used for the analyses:

Bureau of Labor Statistics, U.S. Department of Labor, and National Institute for Child Health and Human Development. Children of the NLSY79, 1979-2016. Produced and distributed by the Center for Human Resource Research (CHRR), The Ohio State University. Columbus, OH: 2019.

We created a dataset by selecting the relevant variables from the Child and Young Adult self-report surveys using the NLS Investigator tool (dataset created on May 9th, 2023)

Data access: Data can be accessed and directly downloaded via the NLS investigator tool

NSHAP

Panel Name: National Social Life, Health, and Aging Project (NSHAP)

Description: The National Social Life, Health, and Aging Project (NSHAP) is a longitudinal, population-based study of health and social factors. It is conducted to understand the well-being of older adults by investigating associations between various factors, such as physical health, emotional health, social connectedness, sexuality, and relationship quality.

Face-to-face interviews were conducted on more than 3,000 respondents, and data was collected in three waves

More information at: https://www.norc.org/content/norc-org/us/en/research/projects/national-social-life-health-and-aging-project.html

Country/Countries: United States

Waves included in the analyses: Round 1, Round 2, Round 3

Data collection period (of waves included in the analyses): 2005 - 2016

Dataset(s) version number/name used for the analyses: Round 1: Waite, Linda J., Laumann, Edward O., Levinson, Wendy S., Lindau, Stacy Tessler, and O'Muircheartaigh, Colm A. National Social Life, Health, and Aging Project (NSHAP): Round 1, [United States], 2005-2006. Inter-university Consortium for Political and Social Research [distributor], 2023-01-30. https://doi.org/10.3886/ICPSR20541.v10

Round 2: Waite, Linda J., Cagney, Kathleen A., Dale, William, Huang, Elbert S., Laumann, Edward O., McClintock, Martha K., ... Cornwell, Benjamin. National Social Life, Health, and Aging Project (NSHAP): Round 2 and Partner Data Collection, [United States], 2010-2011. Inter-university Consortium for Political and Social Research [distributor], 2023-05-24. https://doi.org/10.3886/ICPSR34921.v5

Round 3: Waite, Linda J., Cagney, Kathleen A., Dale, William, Hawkley, Louise C., Huang, Elbert S., Lauderdale, Diane S., ... Schumm, L. Philip. National Social Life, Health, and Aging Project (NSHAP): Round 3 and COVID-19 Study, [United States], 2015-2016, 2020-2021. Inter-university Consortium for Political and Social Research [distributor], 2022-11-17. https://doi.org/10.3886/ICPSR36873.v7

Data access: The Public-Use data set can be downloaded from the ICPSR-NACDA portal.

PHF

Panel Name: Deutsche Bundesbank Panel on Household Finances (PHF)

Description: The German Panel on Household Finances (PHF) is a panel survey on household finance and wealth in Germany, covering the balance sheet, pension, income, work life and other demographic characteristics of private households living in Germany. The first wave of the PHF was carried out in 2010/2011, the second and third wave in 2014 and 2017, respectively. In the first wave, around 3,500 randomly selected households participated, from which about 2,200 also participated in the second wave.

This paper uses data from the Deutsche Bundesbank Panel on Household Finances. The results published and the related observations and analysis may not correspond to results or analysis of the data producers.

More information at: https://www.bundesbank.de/en/bundesbank/research/panel-on-household-finances

Country/Countries: Germany

Waves included in the analyses: Wave 1, Wave 2, Wave 3

Data collection period (of waves included in the analyses): 2010-2017

Dataset(s) version number/name used for the analyses:

PHF Scientific Use File data sets

Wave 1 Version 4.0 DOI: 10.12757/Bbk.PHF.01.04.01

Wave 2 Version 4.0. DOI: 10.12757/Bbk.PHF.02.04.01

Wave 3 Version 2.0. DOI: 10.12757/Bbk.PHF.03.02.01

Data access: Access to the data can be requested via the Deutsche Bundesbank Eurosystem PHF Data Access page

SAVE

Panel Name: Sparen und Altersvorsorge in Deutschland (SAVE)

Description: The Sparen und Altersvorsorge in Deutschland (SAVE) is a representative, longitudinal study on households' financial behavior with a special focus on savings and old-age provision. Started in 2001, SAVE has collected data on households' financial structure and relevant socio- and psychological aspects until 2013.

Country/Countries: Germany

Waves included in the analyses: 2001, 2003-2004, 2005, 2006, 2007, 2008, 2009, 2010, 2013

Data collection period (of waves included in the analyses): 2001-2013

Dataset(s) version number/name used for the analyses:

Börsch-Supan, Axel, & Essig, Lothar (2004). Saving and old-age provision in Germany (SAVE) 2001. GESIS Data Archive, Cologne. ZA4051 Data file Version 1.0.0, https://doi.org/10.4232/1.4051.

Börsch-Supan, Axel, Schunk, Daniel, & Essig, Lothar (2006). Saving and old-age provision in Germany (SAVE) 2003/04. GESIS Data Archive, Cologne. ZA4436 Data file Version 1.0.0, https://doi.org/10.4232/1.4436 . Börsch-Supan, Axel, & Schunk, Daniel (2006). Saving and old-age provision in Germany (SAVE) 2005. GESIS Data Archive, Cologne. ZA4437 Data file Version 1.0.0, https://doi.org/10.4232/1.4437.

Börsch-Supan, Axel, & Schunk, Daniel (2007). Saving and old-age provision in Germany (SAVE) 2006. GESIS Data Archive, Cologne. ZA4521 Data file Version 1.0.0, https://doi.org/10.4232/1.4521.

Börsch-Supan, Axel, & Coppola, Michela (2007). Saving and old-age provision in Germany (SAVE) 2007. GESIS Data Archive, Cologne. ZA4740 Data file Version 1.0.0, https://doi.org/10.4232/1.4740.

Börsch-Supan, Axel, Coppola, Michela, & Ziegelmeyer, Michael (2009). Saving and old-age provision in Germany (SAVE) 2008. GESIS Data Archive, Cologne. ZA4970 Data file Version 1.0.0, https://doi.org/10.4232/1.4970.

Börsch-Supan, Axel, Coppola, Michela, & Ziegelmeyer, Michael (2010). Saving and old-age provision in Germany (SAVE) 2009. GESIS Data Archive, Cologne. ZA5230 Data file Version 1.0.0, https://doi.org/10.4232/1.10062.

Börsch-Supan, Axel, Coppola, Michela, & Ziegelmeyer, Michael (2011). Saving and old-age provision in Germany (SAVE) 2010. GESIS Data Archive, Cologne. ZA5292 Data file Version 1.0.0, https://doi.org/10.4232/1.10423.

Börsch-Supan, Axel, Coppola, Michela, Lamla, Bettina, & Bucher-Koenen, Tabea (2014). Saving and old-age provision in Germany (SAVE) 2013. GESIS Data Archive, Cologne. ZA5647 Data file Version 1.0.0, https://doi.org/10.4232/1.11886.

Data access: Access to the data can be requested on the GESIS webpage

SHARE

Panel Name: Survey of Health, Ageing and Retirement in Europe (SHARE)

Description: The Survey of Health, Ageing and Retirement in Europe (SHARE) is a research infrastructure for studying the effects of health, social, economic and environmental policies over the life-course of European citizens and beyond. From 2004 until today, 140,000 people aged 50 or older from 28 European countries and Israel have been interviewed in 8 waves. SHARE is the largest pan-European social science panel study providing internationally comparable longitudinal micro data which allow insights in the fields of public health and socio-economic living conditions of European individuals.

More information at: https://share-eric.eu/

Börsch-Supan, A., M. Brandt, C. Hunkler, T. Kneip, J. Korbmacher, F. Malter, B. Schaan, S. Stuck, S. Zuber (2013). Data Resource Profile: The Survey of Health, Ageing and Retirement in Europe (SHARE). International Journal of Epidemiology. DOI: 10.1093/ije/dyt088

Country/Countries: Austria, Belgium, Czech_Rep, Denmark, Estonia, France, Germany, Israel, Italy, Netherlands, Slovenia, Spain, Sweden, Switzerland.

Waves included in the analyses: Wave 1, Wave 2, Wave 4, Wave 5, Wave 6, Wave 7, Wave 8

Data collection period (of waves included in the analyses): 2004-2020

Dataset(s) version number/name:

This paper uses data from SHARE Waves 1, 2, 4, 5, 6, 7, and 8 (DOIs: 10.6103/SHARE.w1.710, 10.6103/SHARE.w2.710, 10.6103/SHARE.w4.710, 10.6103/SHARE.w5.710, 10.6103/SHARE.w6.710, 10.6103/SHARE.w7.711, 10.6103/SHARE.w8.100) see Börsch-Supan et al. (2013) for methodological details.(1) The SHARE data collection has been funded by the European Commission, DG RTD through FP5 (QLK6-CT-2001-00360), FP6 (SHARE-I3: RII-CT-2006-062193, COMPARE: CIT5-CT-2005-028857, SHARELIFE: CIT4-CT-2006-028812), FP7 (SHARE-PREP: GA N°211909, SHARE-LEAP: GA N°227822, SHARE M4: GA N°261982, DASISH: GA N°283646) and Horizon 2020 (SHARE-DEV3: GA N°676536, SHARE-COHESION: GA N°870628, SERISS: GA N°654221, SSHOC: GA N°823782, SHARE-COVID19: GA N°101015924) and by DG Employment, Social Affairs & Inclusion through VS 2015/0195, VS 2016/0135, VS 2018/0285, VS 2019/0332, VS 2020/0313 and SHARE-EUCOV: GA N° 101052589 and EUCOVII: GA N°101102412. Additional funding from the German Ministry of Education and Research, the Max Planck Society for the Advancement of Science, the U.S. National Institute on Aging (U01_AG09740-13S2, P01_AG005842, P01_AG08291, P30_AG12815, R21_AG025169, Y1-AG-4553-01, IAG_BSR06-11, OGHA_04-064, BSR12-04, R01_AG052527-02, HHSN271201300071C, RAG052527A) and from various national funding sources is gratefully acknowledged (see www.share-eric.eu).

SHARE-ERIC (2020). Survey of Health, Ageing and Retirement in Europe (SHARE) Wave 1. Release version: 7.1.0. SHARE-ERIC. Data set. DOI: 10.6103/SHARE.w1.710

SHARE-ERIC (2020). Survey of Health, Ageing and Retirement in Europe (SHARE) Wave 2. Release version: 7.1.0. SHARE-ERIC. Data set. DOI: 10.6103/SHARE.w2.710

SHARE-ERIC (2020). Survey of Health, Ageing and Retirement in Europe (SHARE) Wave 4. Release version: 7.1.0. SHARE-ERIC. Data set. DOI: 10.6103/SHARE.w4.710

SHARE-ERIC (2020). Survey of Health, Ageing and Retirement in Europe (SHARE) Wave 5. Release version: 7.1.0. SHARE-ERIC. Data set. DOI: 10.6103/SHARE.w5.710

SHARE-ERIC (2020). Survey of Health, Ageing and Retirement in Europe (SHARE) Wave 6. Release version: 7.1.0. SHARE-ERIC. Data set. DOI: 10.6103/SHARE.w6.710

SHARE-ERIC (2020). Survey of Health, Ageing and Retirement in Europe (SHARE) Wave 7. Release version: 7.1.1. SHARE-ERIC. Data set. DOI: 10.6103/SHARE.w7.711

SHARE-ERIC (2021). Survey of Health, Ageing and Retirement in Europe (SHARE) Wave 8. Release version: 1.0.0. SHARE-ERIC. Data set. DOI: 10.6103/SHARE.w8.100

Data access: Data access can be requested via the Data Access page of the SHARE website.

SOEP

Panel Name: German Socio-Economic Panel (SOEP)

Description: The Socio-Economic Panel (SOEP) is a longitudinal study of private households in Germany. It is one of the largest and longest-running multidisciplinary household surveys worldwide. Every year, approximately 30,000 people in 15,000 households are interviewed. SOEP questionnaires cover various topics such as, healthcare, family life and personality assessments. Data collection began in 1984, and households are surveyed on a annual basis.

Jan Goebel, Markus M. Grabka, Stefan Liebig, Martin Kroh, David Richter, Carsten Schröder, Jürgen Schupp (2018) The German Socio-Economic Panel Study (SOEP). Jahrbücher für Nationalökonomie und Statistik / Journal of Economics and Statistics (online first), doi: 10.1515/jbnst-2018-0022

More information at: https://www.diw.de/en/diw_02.c.299726.en/soep_overview.html

Country/Countries: Germany

Waves: 1984-2020

Data collection period: 1984-2020

Dataset(s) version number/name used for the analyses: Socio-Economic Panel (SOEP), data for years 1984-2020, version 37, SOEP, 2020, 10.5684/soep.core.v37eu.

Data access: Access to the data can be requested on the DIW Berlin's SOEP Research Data Center Data Access webpage

TWINLIFE

Panel Name: TwinLife (TWINLIFE)

Description: TwinLife is a longitudinal, interdisciplinary twin family study on the development of social inequality. It takes a genetically informed life course perspective on social inequalities that acknowledges the importance of both genetic and social influences, social structure, and individual agency. Data collection began in 2014 with a population-based sample of 4,097 twin families. The cross-sequential survey design contains four twin birth cohorts with ~1,000 same-sex (both monozygotic and dizygotic) twin pairs. Face-to-face interviews within the households take place every other year, and telephone interviews are conducted in the consecutive years.

More information available at: https://www.twin-life.de/studie-twinlife

Country/Countries: Germany

Waves included in the analyses: Face-to-face 1 (F2F 1 [wid1]); Face-to-face 2 (F2F 2 [wid3]); Face-to-Face 3 (F2F 3 [wid5])

Data collection period (of waves included in the analyses): 2015-2019 (also refer here)

Dataset(s) version number/name used for the analyses: Diewald, M., Riemann, R., Spinath, F. M., Gottschling, J., Hahn, E., Kornadt, A. E., ... & Weigel, L. (2020). TwinLife. GESIS Data Archive, Cologne. ZA6701 Data file Version 6.1.0, https://doi.org/10.4232/1.13987

(specific data files: ZA6701_person_wid1_v6-1-0; ZA6701_person_wid3_v6-1-0; ZA6701_person_wid5_v6-1-0)

Data access: Access to the data can be requested on the GESIS ZA6701 study webpage

UAS

Panel Name: Understanding America Study (UAS)

Description: The Understanding America Study (UAS) is a panel of about 12,000 respondents representing the entire United States of America. Respondents complete surveys on a variety of topics via their computer, tablet, or smart phone.

More information at: https://uasdata.usc.edu/index.php

Country/Countries: United States of America

Waves included in the analyses:

The project described in this paper relies on data from survey(s) administered by the Understanding America Study, which is maintained by the Center for Economic and Social Research (CESR) at the University of Southern California. The content of this paper is solely the responsibility of the authors and does not necessarily represent the official views of USC or UAS.

Surveys - UAS185, UAS20, UAS396, UAS95, UAS411, UAS242, UAS244, UAS246, UAS250, UAS254, UAS256, UAS258, UAS260, UAS262, UAS264, UAS266, UAS268, UAS270, UAS272, UAS274, UAS276, UAS278, UAS280, UAS282, UAS340, UAS342, UAS344, UAS346, UAS348, UAS240, UAS248, UAS252, UAS182, UAS230, UAS235, UAS164, UAS193, UAS331, UAS65, UAS166, UAS226, UAS117

Data collection period (of waves included in the analyses): 2015-2021

Dataset(s) version number/name: NA

Data access: To access the data, refer to About the data page on https://uasdata.usc.edu/index.php

ULMS

Panel Name: Ukrainian Longitudinal Monitoring Survey (ULMS)

Description: The Ukrainian Longitudinal Monitoring Survey was aimed at obtaining information on the active adult population of Ukraine about employment, education and health.

H. Lehmann, A. Muravyev & Zimmermann, K.F.. (2012). "The Ukrainian Longitudinal Monitoring Survey: Towards a Better Understanding of Labor Markets in Transition", in IZA Journal of Labor and Development, 1, Article 9.

More information at: https://datasets.iza.org/dataset/56/ukrainian-longitudinal-monitoring-survey

Country/Countries: Ukraine

Data collection period (of waves included in the analyses): 2003, 2004, 2007 and 2012

Data collection period: 2003-2012

Dataset(s) version number/name:

Institute of Labor Economics (IZA) (2014). The Ukrainian Longitudinal Monitoring Survey. Research Data Center of IZA (IDSC). Version 1.0. doi:10.15185/izadp.7090.1

Lehmann, Hartmut; Muravyev, Aleksander; Kiev International Institute of Sociology, KIIS; Centre for Economic Reform and Transformation, CERT; Economics Education and Research Consortium-Ukraine, EERC; Rheinisch-Westfälisches Institut für Wirtschaftsforschung-Essen, RWI, 2023, “Ukrainian Longitudinal Monitoring Survey”, https://doi.org/10.15185/izadp.7090.1, Research Data Center of IZA (IDSC), V1

Data access: Data can be accessed via the IZA portal.

USOC_IP

Panel Name: UK Household Longitudinal Survey-Innovation Panel (USOC-IP)

Description: The Innovation Panel (IP) is a separate survey, conducted as part of the UK Household Longitudinal Study, Understanding Society. It is designed for experimental and methodological research relevant to longitudinal surveys. Data collection procedures are similar to the Understanding Society survey. Each person aged 16 or older answers the individual adult interview, including and self-completion questionnaire. Young people aged 10 to 15 years are asked to respond to a paper self-completion questionnaire. The survey started in 2008 and has been continuing to collect data annually.

Understanding Society is an initiative funded by the Economic and Social Research Council and various Government Departments, with scientific leadership by the Institute for Social and Economic Research, University of Essex, and survey delivery by the National Centre for Social Research (NatCen) and Verian (formerly Kantar Public). The research data are distributed by the UK Data Service. The COVID-19 study (2020-2021) was funded by the Economic and Social Research Council and the Health Foundation. Serology testing was funded by the COVID-19 Longitudinal Health and Wealth – National Core Study. Fieldwork for the web survey was carried out by Ipsos MORI and for the telephone survey by Kantar.

More information at: https://www.understandingsociety.ac.uk/documentation/innovation-panel

Country/Countries: United Kingdom

Waves: Wave 1-Wave 13

Data collection period: 2008-2020

Dataset(s) version number/name: University of Essex, Institute for Social and Economic Research. (2023). Understanding Society: Innovation Panel, Waves 1-13, 2008-2020. [data collection]. 11th Edition. UK Data Service. SN: 6849, DOI: http://doi.org/10.5255/UKDA-SN-6849-14

Data access: Data can be requested and downloaded via the UK Data Service catalogue

Research involving human participants, their data, or biological material

Policy information about studies with human participants or human data. See also policy information about sex, gender (identity/presentation), and sexual orientation and race, ethnicity and racism.

Reporting on sex and gender	We collected the information on the gender of the respondents from the original datasets (some datasets labeled the variable gender and others sex). When processing the raw data, we computed separate effect sizes for each gender, and when analyzing the data we accounted for the effect of gender.
Reporting on race, ethnicity, or other socially relevant groupings	We did not include socially constructed or socially relevant variables directly in our analyses. As panels included in our analyses collected data in different countries, we computed effect sizes for each country separately (i.e., sample), and included "sample" as a random grouping variable in our analyses
Population characteristics	We collected the information on the age and gender of the respondents from the original datasets.
Recruitment	We did not recruit participants for this study. We used data from existing datasets (i.e., secondary data analysis).
Ethics oversight	The study represent secondary research of de-identified participants an therefore doe not require ethical approval.

Note that full information on the approval of the study protocol must also be provided in the manuscript.

Field-specific reporting

Please select the one below that is the best fit for your research. If you are not sure, read the appropriate sections before making your selection.

☐ Life sciences ☒ Behavioural & social sciences ☐ Ecological, evolutionary & environmental sciences

Behavioural & social sciences study design

All studies must disclose on these points even when the disclosure is negative.

Study description	The study was an individual participant data meta-analysis. We used longitudinal data from 33 panels (57 samples). The data are quantitative.
Research sample	For this study we identified existing datasets that would allow us to perform test-retest and convergent validity analyses. For this purpose, we adopted a systematic method to identify longitudinal data sets including measures of risk preference and fulfilled a set of criteria (see Data collection section below). The descriptions and sources of the datasets used are available on our companion website (https://cdsbasel.github.io/temprisk/data_desc.html). While a few of the 57 samples aimed to be representative of specific populations (e.g., German Socio-economic panel aims to provide a representative sample of the German population), various samples included do not aim or achieve representativeness of the respective populations, so the same should be said of our data as a whole.
Sampling strategy	We aimed for a comprehensive data gathering procedure that would include all publicly available longitudinal data sets containing risk preference measures according to our criteria. Consequently, we did not conduct an a priori power calculation to determine a suitable sample size linked to a particular level of desired power. Rather, the sample size was the number of (unique) respondents across all the included samples whose data was used to compute test-retest correlations and/or inter-correlations. Our sample size exceeds by a few orders of magnitude similar efforts conducted in psychology to examine the test-retest stability of psychological constructs suggesting the sample size should be sufficient for these purposes.
Data collection	In this study we did not directly collect data from participants but, rather, analyzed data from existing datasets (i.e., secondary data analysis). We identified existing datasets by 1) performing searches on general-purpose search engines, survey listings, and data repositories using relevant terms, 2) consulting past literature for references to longitudinal panels or studies, and 3) informal requests to colleagues for suggestions concerning panels or specific studies. This search led to identifying 101 longitudinal panels (157 samples). We then conducted additional steps to determine suitability for our research purposes leading to a selection of 57 samples to be included in our analyses. Given our study relies on existing data, participants in the original studies were blinded to the research question in our study.
Timing	We included data that was available as of May 2023.
Data exclusions	From the list of identified samples (157 samples), we excluded samples that 1) were not publicly available, 2) did not include data on at least one consistently formatted propensity or behavioural measure of risk preference with responses from the same respondents across at least two time points, or that 3) did not record data on the gender and age of the respondents. This criteria led to the exclusion of 100 samples, leaving 57 samples for analysis.<br><br>In each of the 57 samples that were included for analysis, we excluded respondents whose age and/or gender had not been reported or was inconsistently reported across data collection points (i.e., waves). For the computation of test-retest correlations, only respondents who provided (valid) responses to same question in at least two waves were included. For the computation of inter-correlations, only respondents who provided (valid) responses to at least two questions within the same wave were included.
Non-participation	In this study we did not directly collect data from participants, we analyzed data from existing datasets (i.e., secondary data analysis).
Randomization	Participants were not allocated to experimental groups.

Reporting for specific materials, systems and methods

We require information from authors about some types of materials, experimental systems and methods used in many studies. Here, indicate whether each material, system or method listed is relevant to your study. If you are not sure if a list item applies to your research, read the appropriate section before selecting a response.

Materials & experimental systems

n/a	Involved in the study
☒	☐ Antibodies
☒	☐ Eukaryotic cell lines
☒	☐ Palaeontology and archaeology
☒	☐ Animals and other organisms
☒	☐ Clinical data
☒	☐ Dual use research of concern
☒	☐ Plants

Methods

n/a	Involved in the study
☒	☐ ChIP-seq
☒	☐ Flow cytometry
☒	☐ MRI-based neuroimaging
